# Novel Isoxazolidine and γ-Lactam Analogues of Homonucleosides

**DOI:** 10.3390/molecules24224014

**Published:** 2019-11-06

**Authors:** Dorota G. Piotrowska, Iwona E. Głowacka, Dominique Schols, Robert Snoeck, Graciela Andrei, Joanna Gotkowska

**Affiliations:** 1 Bioorganic Chemistry Laboratory, Faculty of Pharmacy, Medical University of Lodz, Muszynskiego 1, 90-151 Lodz, Poland; iwona.glowacka@umed.lodz.pl (I.E.G.); joanna.gotkowska@umed.lodz.pl (J.G.); 2 Rega Institute for Medical Research, KU Leuven, Herestraat 49, B-3000 Leuven, Belgium

**Keywords:** isoxazolidines, γ-lactams, nucleoside analogues, cytostatic activity

## Abstract

Homonucleoside analogues *cis*-**16** and *trans*-**17** having a (5-methoxycarbonyl)isoxazolidine framework were synthesized via the 1,3-dipolar cycloaddition of nucleobase-derived nitrones with methyl acrylate. Hydrogenolysis of the isoxazolidines containing thymine, dihydrouracil, theophylline and adenine moieties efficiently led to the formation of the respective γ-lactam analogues. γ-Lactam analogues having 5-bromouracil and 5-chlorouracil fragments were synthesized by treatment of uracil-containing γ-lactams with NBS and NCS. Isoxazolidine and γ-lactam analogues of homonucleosides obtained herein were evaluated for activity against a broad range of DNA and RNA viruses. None of the compounds that were tested exhibited antiviral or cytotoxic activity at concentrations up to 100 µM. The cytostatic activities of all compounds toward nine cancerous cell lines was tested. γ-Lactams *trans*-**15e** (Cl-Ura) and *cis*-**15h** (Theo) appeared the most active toward pancreatic adenocarcinoma cells (Capan-1), showing IC_50_ values 21.5 and 18.2 µM, respectively. Isoxazolidine *cis*-**15e** (Cl-Ura) inhibited the proliferation of colorectal carcinoma (HCT-116).

## 1. Introduction

**Figure 1:**
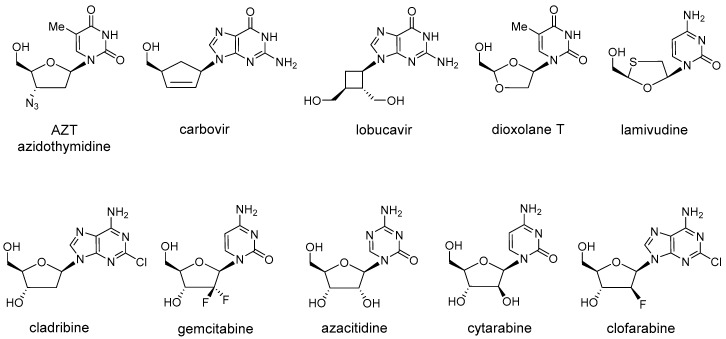
Examples of biologically-active nucleoside analogues.

Nucleoside analogues belong to a group of important antiviral and antitumor drugs [1,2,3,4]. However, resistance to drugs and their toxicity are considered major factors limiting the effectiveness of therapies. Structural modifications of available drugs from a class of nucleoside analogues, including sugar and/or nucleobase residues, resulted in the discovery of a variety of therapeutically-useful antiviral [5] (e.g., azidothymidine (AZT) [6], carbovir [7], lobucavir [8], dioxolane T [9] and lamivudine [10]) and anticancer [11,12] (e.g., cladribine [13], gemcitabine [14], azacitidine [15] and cytarabine [14,16], clofarabine [17]) agents (Figure 1).

**Figure 2:**
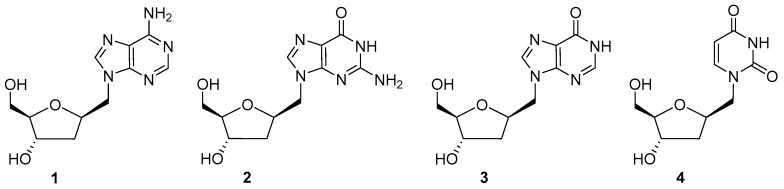
Examples of biologically active 1′-homonucleosides.

On the other hand, the introduction of a methylene bridge between a nucleobase and the sugar partly led to the formation of homonucleosides, which are known for their resistance to hydrolytic or enzymatic cleavage, as well as for the rotational freedom when compared with the natural nucleosides. Furthermore, it was shown that homonucleosides are substrates for cellular kinases and are able to pair with other nucleosides by Watson–Crick interactions without appreciable modifications of the structure of DNA (or RNA) molecules [18]. 1′-Homonucleosides containing adenine **1** or guanine **2** were found to be active against herpes simplex virus (minimum inhibitory concentration (MIC) = 5–20 µg/mL) and vaccinia virus [19,20,21], while compound **3** (IC_50_ = 25.2 µg/mL) showed activity against the influenza type virus AH1N1 (Figure 2) [22]. Homonucleoside analogue of 2′-deoxyuridine **4** has been described as a selective inhibitor of viral uracil-DNA glycosylase (UDG), while exerting only small effect on the human enzymes [23].

**Figure 3:**
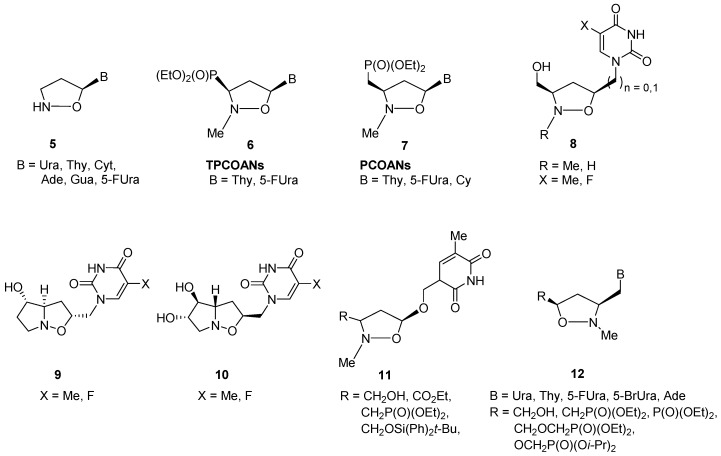
Examples of isoxazolidine nucleoside/nucleotide analogues.

Incorporation of the isoxazolidine ring into a nucleoside framework as a sugar part replacer was first proposed by Tronchet and co-workers [24,25] with the intention to study antiviral [20,26,27,28,29,30,31], antibacterial [32] and antifungal [33] activities of the compounds. Other research groups then intensively explored this field further. Thus, a compound **5** [34] and phosphonylated derivatives **6** [35] and **7** [36,37,38] inhibited the reverse transcriptase activity of avian Moloney virus (AMV) and human immunodeficiency virus (HIV) at a level comparable with that of tenofovir (1 nM) and 10-fold higher than that of AZT (10 nM). Moreover, their cytotoxicity was significantly lower (50% cytotoxic concentration (CC_50_) > 500 µM) in comparison with that of AZT (CC_50_ = 12.14 µM) (Figure 3). In order to obtain compounds with a sufficient stability for hydrolytic or enzymatic cleavage, analogues of 1′-homonucleosides **8**–**11** (Figure 3) were also synthesized [39,40,41,42,43].

**Figure 4:**
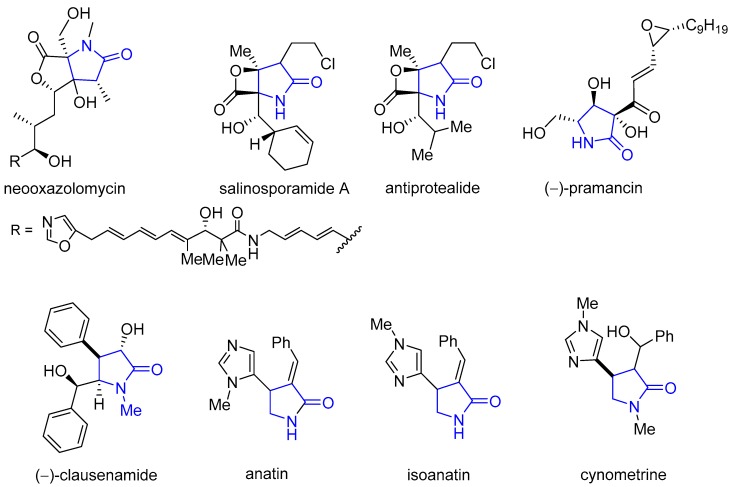
Examples of some bioactive natural molecules containing a γ-lactam core.

**Scheme 1:**
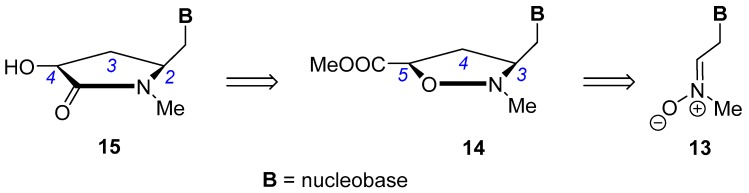
Retrosynthesis of γ-lactam analogues of 1′-homonucleosides **15**.

In the isoxazolidine nucleoside/nucleotide analogues described so far, a nucleobase is attached to C5; therefore, the 1,3-dipolar cycloaddition of nitrones and *N*-vinyl or *N*-allyl nucleobases has been found a convenient method for their preparation [44]. Recently, we designed isoxazolidine analogues of 1′-homonucleos(t)ides **12** (Figure 3), in which nucleobases were attached to C3 to underscore the fundamental difference to the already-described ones [45,46]. In the synthesis of homonucleosides **12**, the 1,3-dipolar cycloaddition was also employed, but in that case, nucleobase-derived nitrones and selected alkenes were applied [45,46]. Since no significant activity of compounds **12** toward the viruses tested was noticed, we wondered whether the replacement of an isoxazolidine moiety with a γ-lactam ring would lead to an improvement of antiviral properties of newly-designed compounds **15**. The idea of incorporation of the γ-lactam moiety has been supported by the presence of this subunit in many natural products (Figure 4), which showed antiviral [47,48], cytotoxic [49,50,51,52,53], anti-inflammatory [53,54,55] and antimicrobial [56,57] properties, among others. For this reason the assumption that homonucleoside analogues **15** (Scheme 1) having the γ-lactam moiety could possess interesting biological properties as antiviral or cytotoxic is justified [58].

## 2. Results and Discussion

### 2.1. Chemistry

The synthetic strategy for γ-lactam homonucleosides **15** relies on the 1,3-dipolar cycloaddition of nucleobase-derived nitrones **13** with methyl acrylate, followed by the hydrogenolysis of the N–O bond in an isoxazolidine scaffold, which was accompanied by the intramolecular cyclization to transform cycloadducts **14** into compounds **15** (Scheme 1).

**Scheme 2:**
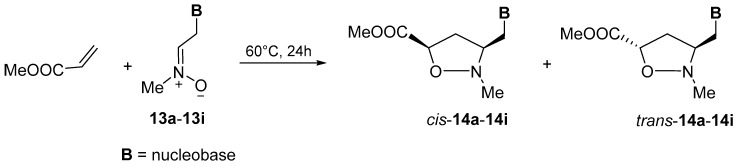
Synthesis of isoxazolidine analogues of 1′-homonucleosides *trans*-**14** and *cis*-**14**.

Nucleobase-derived nitrones **13** were synthesized from N-(2-oxoethyl)nucleobases and *N*-methylhydroxylamine, as described previously [45,46]. The 1,3-dipolar cycloadditions of the nucleobase-derived nitrones **13** to methyl acrylate were carried out at 60 °C in methanol for 24 h (Scheme 2, Table 1) and afforded mixtures of diastereoisomeric isoxazolidines *cis*-**14** and *trans*-**14**. The *cis*/*trans* ratios of the isoxazolidines were calculated from the ^1^H-NMR spectra of the crude reaction mixtures by the comparison of diagnostic resonances of the H_2_C-4 protons in the isoxazolidine ring, as well as the signals of the respective protons of nucleobase moieties. In some cases, differences in resonances of the CH_3_-N protons for diastereoisomeric isoxazolidines *cis*-**14** and *trans*-**14** were also diagnostic. The crude reaction mixtures of the diastereoisomers were subjected to column chromatography; however, only pure isomeric isoxazolidines *cis*-**14a** and *trans*-**14a** were isolated (Table 1, entry a). In the other cases the respective fractions enriched in the isomers *cis*-**14b**–**14i** or *trans*-**14b**–**14i** were collected. The subsequent separation of diastereoisomeric mixture of isoxazolidines *cis*-**14b**–**14h** or *trans*-**14b**–**14h** on an HPLC column allowed us to obtain pure isomers *cis*-**14b**–**14h** and *trans*-**14b**–**14h** (Table 1, entry b–h), and the amounts, however small, were sufficient to perform a full characterization of the newly-synthesized compounds and their further biological screening. Unfortunately, the attempts at separating diastereoisomeric isoxazolidines *cis*-**14i** and *trans*-**14i** appeared fruitless, even with HPLC.

**Scheme 3:**
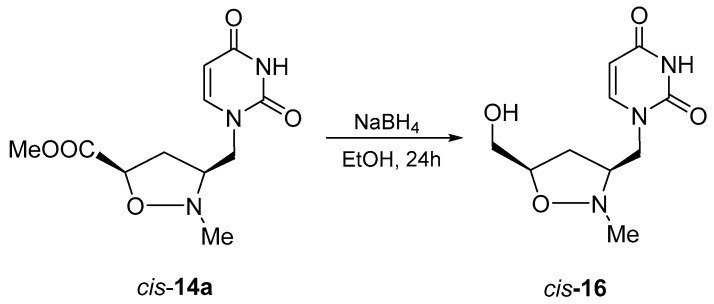
Synthesis of the isoxazolidine *cis*-**16** [45].

The relative configurations of isoxazolidines *cis*-**14a** and *trans*-**14a** have already been established [45]. As we have recently described, (5-methoxycarbonyl)isoxazolidine *cis*-**14a** was reduced with sodium borohydride in ethanol to the known (5-hydroxymethyl)isoxazolidine *cis*-**16** [45] (Scheme 3). During this transformation, the structure of the isoxazolidine skeleton remains unchanged, and therefore, a relative configuration of the isoxazolidine *cis*-**14a** was unambiguously established. Consequently, the isomeric (5-methoxycarbonyl)isoxazolidine was identified as *trans*-**14a**. Since similar spectral patterns for the respective isoxazolidine protons (H3, H4ɑ, H4β and H5) were observed in series of *cis*-**14b**–**14i** and *trans*-**14b**–**14i** when compared to those observed for cycloadducts *cis*-**14a** and *trans*-**14a**, their configurations were analogously assigned.

**Figure 5:**
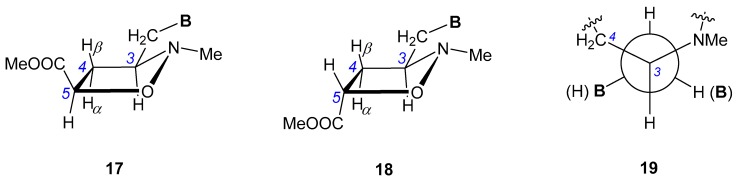
Preferred conformations **17** and **18** of isoxazolidines *cis*-**14a**–**14i** and *trans*-**14a**–**14i**, respectively, and the Newman projection **19**.

A detailed conformational analysis for isoxazolidines *cis*-**14a**–**14i** and *trans*-**14a**–**14i** was performed based on HCCH vicinal coupling constants extracted from the ^1^H-NMR spectra. The vicinal couplings for *cis*-**14a**–**14f** and *cis*-**14h**–**14i** (*J*(H3-H4ɑ) = 7.6–8.4 Hz, *J*(H3-H4β) = 2.0–3.3 Hz, *J*(H4ɑ-H5) = 9.7–9.9 Hz, *J*(H4β-H5) = 5.0–5.3 Hz) suggest the ^2^*E* conformation of the isoxazolidine ring in which the sterically bulky nucleobase-CH_2_ units are located pseudoequatorially (Figure 5, structure **17**). The coupling constant values extracted for *trans*-**14a**–**14f** and *trans*-**14h**–**14i** (*J*(H3-H4ɑ) = 7.4–7.8 Hz, *J*(H3-H4β) = 2.0–3.4 Hz, *J*(H4ɑ-H5) = 7.4–7.5 Hz, *J*(H4β-H5) = 8.7–9.0 Hz) bring to mind the ^2^*E* conformation of the isoxazolidine ring in which the nucleobase-CH_2_ substituents again adopt pseudoequatorial positions (Figure 5, structure **18**). Surprisingly, the preferred conformations for dihydrouacil-containing isoxazolidines *cis*-**14g** and *trans*-**14g** could not be assigned, and sets of couplings indicate the existence of conformational equilibrium of isoxazolidine ring.

For the either cis or trans isoxazolidines containing uracil or substituted uracil residues **14a**–**14f**, we also noticed that diastereotopic hydrogen atoms in CH_2_–base units which resonate at a lower field (4.05–3.89 ppm) showed significantly smaller vicinal coupling constants to H–C3 (3.3–3.9 Hz) than those associated with higher field signals (3.59–3.32 ppm) when values of 9.1–9.7 Hz were observed. One may conclude that high-field hydrogens are antiperiplanar to H–C3, while low-field ones are oriented gauche, as depicted by the Newman projection **19** (Figure 5). Although real reasons for the restricted rotation around C3–CH_2_Base bond remain obscure to us at the moment, they may simply reflect repulsive interactions of sterically bulky CH_2_–base groups with Me–N and Hβ–C4–Hα fragments.

**Scheme 4:**
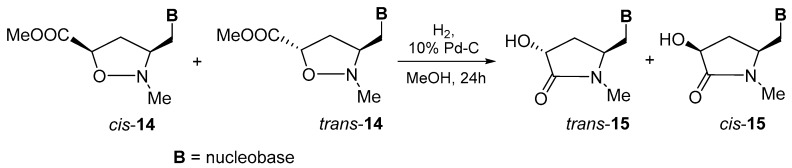
Synthesis of γ-lactam analogues of 1′-homonucleosides *trans*-**15** and *cis*-**15**.

Finally, attempts at transformation of isoxazolidine cycloadducts *cis*-**14**/*trans*-**14** into γ-lactam derivatives *trans*-**15**/*cis*-**15** were made, following the procedure applied previously for transformation of *cis*-**14a** into *trans*-**15g** [45]. It was expected that since hydrogenation of the N–O bond in the isoxazolidine scaffold releases the amino group, the subsequent intramolecular cyclization to γ-lactams *trans*-**15**/*cis*-**15** should occur spontaneously (Scheme 4). Consequently, the isoxazolidine *cis*-**14** would provide *trans*-**15**, while isoxazolidine *trans*-**14** should be a substrate for the synthesis of *cis*-configured γ-lactam *cis*-**15**. With that in mind, we concluded that not only can pure diastereoisomers *cis*-**14** and *trans*-**14** be used for the synthesis of *trans*-**15**/*cis*-**15**, but so can mixtures of the respective isomers *cis*-**14**/*trans*-**14**, although significantly enriched in one diastereoisomer to maintain control on the stereochemistry of the products formed.

As we recently reported [45], the hydrogenation of uracil-containing isoxazolidine homonucleoside *cis*-**14a** (B = Ura) proceeded smoothly and the intramolecular cyclization of intermediary γ-aminocarboxylic ester to a γ-lactam was accompanied by hydrogenation of the uracil residue to produce the lactam *trans*-**15g** (B = diH-Ura) in a good yield (80%) instead of the expected compound *trans*-**15a** (B = Ura) (Table 2, entry a). Similarly, *cis*-**15g** (B = diH-Ura) was obtained from *trans*-**14a** (B = Ura) (85%).

On the other hand, when a 80:20 mixture of thymine-containing isoxazolidines *cis*-**14b** and *trans*-**14b** (B = Thy) was subjected to hydrogenation, the respective 80:20 mixture of γ-lactam *trans*-**15b** and *cis*-**15b** (B = Thy) was obtained. From this mixture pure diastereoisomers were isolated after column chromatography followed by HPLC (Table 2, entry b).

During the hydrogenation of 5-halogenated uracil derivatives *cis*-**14**/*trans*-**14** (B = X-Ura), we faced the same problem as in case of uracil derivatives. Moreover, halogen atoms were removed from nucleobase skeletons. For example, hydrogenation of a 60:40 mixture of *cis*-**14d** and *trans*-**14d** isoxazolidines (B = Br-Ura) led to the formation of an inseparable mixture of *trans*-**15a**, *cis*-**15a** and *trans*-**15g** and *cis*-**15g** (53:35:8:2) (Table 2, entry d). Analogously, a mixture of *trans*-**15a**, *cis*-**15a**, *trans*-**15g** and *cis*-**15g** (4:2:74:20) was obtained from the 73:27 mixture of isoxazolidines *cis*-**14e** and *trans*-**14e** (B = Cl-Ura) (Table 2, entry e). Similarly, hydrogenation of a *cis*-**14c** (B = F-Ura) gave dihydrouracil-containing γ-lactam *trans*-**15g** (B = diH-Ura) (Table 2, entry c).

Furthermore, the respective mixtures of isoxazolidines *cis*-**14g**/*trans*-**14g**, *cis*-**14h**/*trans*-**14h** and *cis*-**14i**/*trans*-**14i** were successfully hydrogenated to provide γ-lactams containing dihydrouracil (*trans*-**15g**/*cis*-**15g**), theophylline (*trans*-**15h**/*cis*-**15h**) and adenine (*trans*-**15i**/*cis*-**15i**) as nucleobases (Table 2, entry g–i).

**Scheme 5:**
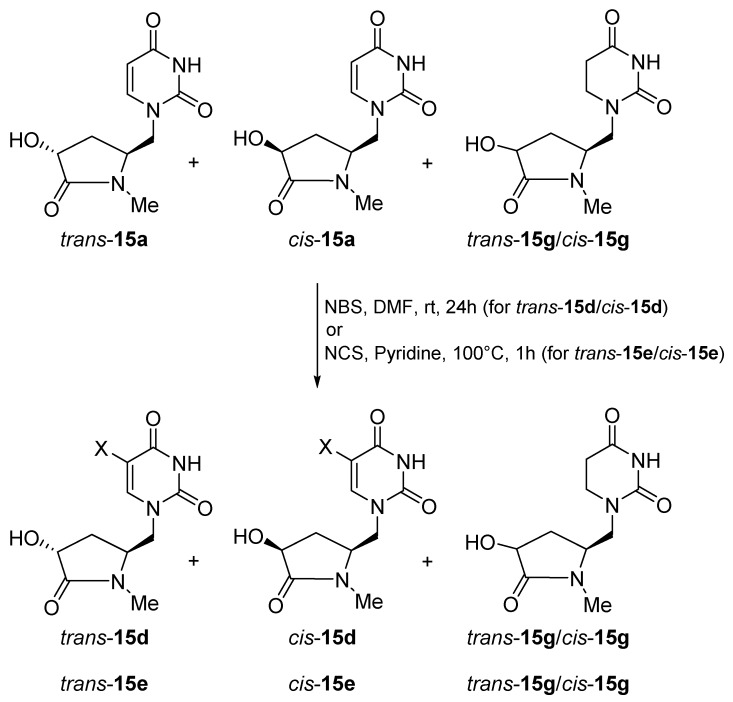
Synthesis of γ-lactams *trans*-**15d** and *cis*-**15d** (X = Br), and *trans*-**15e** and *cis*-**15e** (X = Cl).

To supplement a library of designed γ-lactam homonucleosides *trans*-**15**/*cis*-**15** with analogues having bromine (*trans*-**15d**/*cis*-**15d**) and chlorine (*trans*-**15e**/*cis*-**15e**) atoms, the halogenation of uracil derivatives was proposed [59,60,61]. Treatment of the above-mentioned (Table 1, entry d), inseparable mixture of γ-lactams *trans*-**15a**, *cis*-**15a**, *trans*-**15g** and *trans*-**15g** (53:35:8:2) with *N*-bromosuccinimide (NBS) [59] led to the formation of the mixture of γ-lactams *trans*-**15d**, *cis*-**15d**, together with unreacted *trans*-**15g** and *cis*-**15g** (50:37:9:4) (Scheme 5). The purification of this mixture on a silica gel column followed by HPLC allowed us to isolate *trans*-**15d** and *cis*-**15d** in 30% and 9.3% yields, respectively. Similarly, the reaction of an analogous mixture of *trans*-**15a**, *cis*-**15a**, *trans*-**15g** and *cis*-**15g** with *N*-chlorosuccinimide (NCS) [60] gave the respective mixture of compounds *trans*-**15e**, *cis*-**15e**, *trans*-**15g** and *cis*-**15g** (55:34:8:3), from which *trans*-**15e** and *cis*-**15e** were obtained in 6% and 4.2% yields, respectively (Scheme 5).

### 2.2. Antiviral and Cytostatic Evaluation

#### 2.1.1. Antiviral Activity

All isoxazolidines **14** and γ-lactams **15** obtained were evaluated for their activities against a wide variety of DNA and RNA viruses using the following cell-based assays: (a) human embryonic lung (HEL) cell cultures: herpes simplex virus-1 (KOS strain), herpes simplex virus-2 (G strain), vaccinia virus, thymidine kinase deficient (acyclovir-resistant) herpes simplex virus-1 (TK^−^ KOS ACV^r^), adenovirus-2, human coronavirus (229E strain), cytomegalovirus (AD-169 and Davis strains) and varicella-zoster virus (TK^+^ VZV and TK^−^ VZV strains); (b) HeLa cell cultures: vesicular stomatitis virus, Coxsackie virus B4 and respiratory syncytial virus; (c) Vero cell cultures: para-influenza-3 virus, reovirus-1, Sindbis virus, Coxsackie virus B4, Punta Toro virus and yellow fever virus; and (d) MDCK cell cultures: influenza A virus (H1N1 and H3N2 subtypes) and influenza B virus. Ganciclovir, cidofovir, acyclovir, brivudin, zalcitabine, zanamivir, alovudine, mycophenolic acid, amantadine, rimantadine, ribavirin, dextran sulfate (molecular weight 10000, DS-10000) and Urtica dioica agglutinin (UDA) were used as the reference compounds. The antiviral activity was expressed as the EC_50_: the compound concentration required to reduce virus plaque formation (VZV) by 50% or to reduce virus-induced cytopathicity by 50% (other viruses). The cytotoxicity of the compounds tested toward the uninfected host cells was defined as the minimum cytotoxic concentration (MCC) that causes a microscopically detectable alteration of normal cell morphology. The 50% cytotoxic concentration (CC_50_), causing a 50% decrease in cell viability was determined using a colorimetric MTS assay system. None of the tested compounds showed appreciable antiviral activity toward any of the tested DNA and RNA viruses at the concentration up to 100 μM. At the same time, none of them affected the cell morphologies of used uninfected host cells.

#### 2.1.2. Cytostatic Activity

The 50% cytostatic inhibitory concentration (IC_50_) causing a 50% decrease in cell proliferation was determined for all isolated isoxazolidines **14** and γ-lactams **15** toward nine cancerous cell lines (Capan-1 (pancreatic adenocarcinoma), Hap1 (chronic myeloid leukemia), HCT-116 (colorectal carcinoma), NCI-H460 (lung carcinoma), DND-41 (acute lymphoblastic leukemia), HL-60 (acute myeloid leukemia), K-562 (chronic myeloid leukemia), MM.1S (multiple myeloma) and Z-138 (non-Hodgkin lymphoma)) and normal retina (non-cancerous) cells (hTERT RPE-1). Docetaxel, etoposide and stauroporine were used as the reference compounds.

All of the compounds tested were not toxic to non-cancerous retina cells (hTERT RPE-1) at concentrations up to 100 μM. They were also not active against DND-41, HL-60, K-562, MM.1S or Z-138 cancer cells. All isoxazolidines **14** and γ-lactams **15** we tested, except *cis*-**14c** (B = F-Ura), exhibited moderate activity against pancreatic adenocarcinoma cells (Capan-1) (IC_50_ = 18.2 to 95 µM), and among them, γ-lactams *trans*-**15e** (Cl-Ura) and *cis*-**15h** (Theo) were the most active, with IC_50_ values of 21.5 and 18.2 µM, respectively (Table 3). In most cases, both *cis*-configured isoxazolidines **14** and *cis*-configured γ-lactams **15** were slightly less active when compared to the respective *trans*-isomers (e.g., *cis*-**14b**–**f** versus *trans*-**14b**–**f**, *cis*-**14h**–**i** versus *trans*-**14h**–**I** and *cis*-**15b** versus *trans*-**15b**, *cis*-**15d** vs. *trans*-**15d**); however, that correlation did to apply for uracil, dihydrouracil and theophiline-derivatives **14a**, **15g** and **15h**. Moreover, several isoxazolidines **14** and γ-lactams **15** inhibited the proliferation of Hap1 (IC_50_ = 40.9 to 58.5 µM), HCT-116 (IC_50_ = 26.7 to 68,4 µM) and NCI-H460 cells (IC_50_ = 28.6 to 62.3 µM) (Table 3).

## 3. Experimental Section

### 3.1. General

^1^H-NMR spectra were taken in CDCl_3_, CD_3_OD, D_2_O and DMSO on the following spectrometers: Varian Gemini 2000BB (200 MHz), Varian Mercury-300 and Bruker Avance III spectrometers (600 MHz) with TMS as internal standard. ^13^C-NMR spectra were recorded in CDCl_3_, CD_3_OD, D_2_O and DMSO on the Bruker Avance III spectrometer (at 150 MHz, Bruker Instruments, Karlsruhe, Germany) and a Varian Mercury-300 machine (Varian NMR Instrument, Palo Alto, CA, USA) at 75 MHz. IR spectra were measured on a Bruker Alpha-T FT-IR spectrometer (Bruker Optik GmbH, Ettlingen, Germany). Melting points were determined on Boetius apparatus and are reported uncorrected. Elemental analyses were performed by the Microanalytical Laboratory of our faculty on a Perkin–Elmer PE 2400 CHNS analyzer (Perkin-Elmer Corp., Norwalk, CT, USA). The following adsorbents were used: column chromatography, Merck silica gel 60 (70–230 mesh), analytical TLC, Merck TLC plastic sheets and silica gel 60 F_254_. The preparative HPLC experiment was performed on a Waters chromatograph equipped with a Waters 2545 binary gradient module (Waters Corporation, Milford, MA, USA) and a Waters 2998 photodiode array detector (190–600 nm).

^1^H- and ^13^C-NMR spectra of all new synthesized compounds are provided in Supplementary Materials.

### 3.2. General Procedure for the Synthesis of Isoxazolidines cis-***14*** and trans-***14***

To a solution of a nitrone (1.00 mmol) in methanol (9 mL), methyl acrylate (10.0 mL) was added. The mixture was stirred at 60 °C for 24 h. The solvent was removed in vacuo and crude products were purified on silica gel columns using chloroform-methanol (100:1, 50:1, 20:1, 10:1, *v*/*v*) as eluents. The respective fractions were subjected to HPLC on a X Bridge Prep, C18, 5 µm, OBD, 19 × 100 mm column using water/methanol (90:10, 85:15, *v*/*v*) to afford pure isoxazolidines.

*Methyl cis-3-{[2,4-dioxo-3,4–dihydropyrimidin-1(2H)-yl]methyl}-2-methylisoxazolidine-5-carboxylate* (*cis*-**14a**). Yield: 26%; a colorless amorphous solid; m.p. = 107–108 °C (crystallized from chloroform–methanol). IR (KBr, cm^−1^) *ν*_max_: 3478, 3196, 3097, 3054, 2958, 1684, 1457, 1376, 1252, 1214, 1078, 811; ^1^H-NMR (200 MHz, CDCl_3_) δ: 9.84 (br s, 1H, NH), 7.37 (d, 1H, ^3^*J* = 7.8 Hz), 5.67 (d, 1H, ^3^*J* = 7.8 Hz), 4.74 (dd, 1H, ^3^*J*_(H5–H4α)_ = 9.9 Hz, ^3^*J*_(H5–H4β)_ = 5.1 Hz, HC5), 3.93 (dd, 1H, ^2^*J*_(HCH)_ = 12.9 Hz, ^3^*J*_(HCC–H3)_ = 3.3 Hz, *H*CH-Ura), 3.78 (s, 3H, C(O)OCH_3_), 3.55 (dd, 1H, ^2^*J*_(HCH)_ = 12.9 Hz, ^3^*J*_(HCC–H3)_ = 9.6 Hz, HC*H*-Ura), 3.47 (dddd, 1H, ^3^*J*_(H3–CCH)_ = 9.6 Hz, ^3^*J*_(H3–H4α)_ = 7.8 Hz, ^3^*J*_(H3–CCH)_ = 3.3 Hz, ^3^*J*_(H3–H4*β*)_ = 2.4 Hz, HC3), 2.86 (ddd, 1H, ^2^*J*_(H4α–H4β)_ = 13.5 Hz, ^3^*J*_(H4α–H5)_ = 9.9 Hz, ^3^*J*_(H4*α*–H3)_ = 7.8 Hz, H_α_C4), 2.62 (s, 3H, CH_3_N), 2.17 (ddd, 1H, ^2^*J*_(H4β–H4α)_ = 13.5 Hz, ^3^*J*_(H4β–H5)_ = 5.1 Hz, ^3^*J*_(H4*β*–H3)_ = 2.4 Hz, H_β_C4); ^13^C-NMR (75 MHz, CDCl_3_) δ: 171.45 (*C*(O)OCH_3_), 164.29 (C4′), 151.34 (C2′), 146.82 (C5′), 101.45 (C6′), 74.52 (C5), 65.48 (C3), 52.90 (C(O)O*C*H_3_), 50.82 (CH_2_-Ura), 44.59 (CH_3_N), 34.95 (C4). Anal. Calcd for C_11_H_15_N_3_O_5_: C, 49.07; H, 5.62; N, 15.61. Found: C, 48.99; H, 5.82; N, 15.80.

*Methyl trans-3-{[2,4-dioxo-3,4–dihydropyrimidin-1(2H)-yl]methyl}-2-methylisoxazolidine-5-carboxylate* (*trans*-**14a**). Yield: 19%; a colorless amorphous solid; m.p. = 151–152 °C (crystallized from chloroform–methanol). IR (KBr, cm^−1^) *ν*_max_: 3192, 3045, 2961, 2830, 1730, 1704, 1675, 1451, 1212, 1088, 1039, 914; ^1^H-NMR (600 MHz, CDCl_3_) δ: 9.47 (br s, 1H, NH), 7.30 (d, 1H, ^3^*J* = 7.9 Hz), 5.68 (d, 1H, ^3^*J* = 7.9 Hz), 4.54 (dd, 1H, ^3^*J*_(H5–H4β)_ = 9.0 Hz, ^3^*J*_(H5–H4α)_ = 7.4 Hz, *H*C5), 4.02 (dd, 1H, ^2^*J*_(HCH)_ = 13.9 Hz, ^3^*J*_(HCC–H3)_ = 3.8 Hz, *H*CH-Ura), 3.79 (s, 3H, C(O)OCH_3_), 3.49 (dddd, 1H, ^3^*J*_(H3–CCH)_ = 9.3 Hz, ^3^*J*_(H3–H4α)_ = 7.4 Hz, ^3^*J*_(H3–CCH)_ = 3.8 Hz, ^3^*J*_(H3–H4β)_ = 2.4 Hz HC3), 3.36 (dd, 1H, ^2^*J*_(HCH)_ = 13.9 Hz, ^3^*J*_(HCC–H3)_ = 9.3 Hz, HC*H*-Ura), 2.81 (ddd, 1H, ^2^*J*_(H4α–H4β)_ = 13.3 Hz, ^3^*J*_(H4α–H5)_ = 7.4 Hz, ^3^*J*_(H4α–H3)_ = 7.4 Hz, H_α_C4), 2.68 (s, 3H, CH_3_N), 2.38 (ddd, 1H, ^2^*J*_(H*β*4–H*α*4)_ = 13.3 Hz, ^3^*J*_(H4*β*–H5)_ = 9.0 Hz, ^3^*J*_(H4*β*–H3)_ = 2.4 Hz, H_β_C4); ^13^C-NMR (150 MHz, CDCl_3_) δ: 172.33 (*C*(O)OCH_3_), 163.84 (C4′), 151.14 (C2′), 146.27 (C5′), 101.50 (C6′), 76.28 (C5), 65.73 (C3), 52.58 (C(O)O*C*H_3_), 49.97 (CH_2_-Ura), 45.66 (CH_3_N), 34.64 (C4). Anal. Calcd for C_11_H_15_N_3_O_5_ × 0.25 H_2_O: C, 48.26; H, 5.71; N, 15.35. Found: C, 48.10; H, 5.53; N, 15.02.

*Methyl cis-2-methyl-3-{[5-methyl-2,4-dioxo-3,4–dihydropyrimidin-1(2H)-yl]methyl}isoxazolidine-5-carboxylate* (*cis*-**14b**). Yield: 7.4%; a colorless amorphous solid; m.p. = 73–74 °C (crystallized from chloroform–methanol). IR (KBr, cm^−1^) *ν*_max_: 3168, 3037, 2955, 2893, 2830, 1746, 1701, 1469, 1437, 1214, 1074, 891; ^1^H-NMR (200 MHz, CDCl_3_) δ: 8.63 (br s, 1H, NH), 7.15 (q, 1H, ^4^*J* = 1.1 Hz), 3.82 (dd, 1H, ^3^*J*_(H5–H4α)_ = 9.8 Hz, ^3^*J*_(H5–H4β)_ = 5.2 Hz, *H*C5), 3.82 (dd, 1H, ^2^*J*_(HCH)_ = 12.9 Hz, ^3^*J*_(HCC–H3)_ = 3.3 Hz, *H*CH–Thy), 3.79 (s, 3H, C(O)OCH_3_), 3.55–3.45 (m, 2H, HC*H*–Thy, HC3), 2.86 (ddd, 1H, ^2^*J*_(H4α–H4β)_ = 13.6 Hz, ^3^*J*_(H4α–H5)_ = 9.8 Hz, ^3^*J*_(H4α–H3)_ = 7.6 Hz, H_α_C4), 2.61 (s, 3H, CH_3_N), 2.17 (ddd, 1H, ^2^*J*_(H4β–H4α)_ = 13.6 Hz, ^3^*J*_(H4β–H5)_ = 5.2 Hz, ^3^*J*_(H4β–H3)_ = 2.1 Hz, H_β_C4), 1.91 (d, 3H, ^4^*J* = 1.1 Hz, CH_3_); ^13^C-NMR (150 MHz, CDCl_3_) δ: 171.45 (*C*(O)OCH_3_), 164.16 (C4′), 150.97 (C2′), 142.62 (C5′), 109.69 (C6′), 74.37 (C5), 65.47 (C3), 52.63 (C(O)O*C*H_3_), 50.76 (CH_2_–Thy), 44.49 (CH_3_N), 34.83 (C4), 12.83 (CH_3_). Anal. Calcd for C_12_H_17_N_3_O_5_: C, 50.88; H, 6.05; N, 14.83. Found: C, 50.83; H, 5.86; N, 14.86.

*Methyl trans-2-methyl-3-{[5-methyl-2,4-dioxo-3,4–dihydropyrimidin-1(2H)-yl]methyl}isoxazolidine-5-carboxylate* (*trans*-**14b**). Yield: 17%; a colorless amorphous solid; m.p. = 139–140 °C (crystallized from chloroform–methanol). IR (KBr, cm^−1^) *ν*_max_: 3472, 3175, 3038, 2958, 2929, 2855, 2822, 1745, 1688, 1468, 1436, 1202, 1058; ^1^H-NMR (600 MHz, CDCl_3_) δ: 8.59 (br s, 1H, N*H*), 7.13 (s, 1H), 4.56 (dd, 1H, ^3^*J*_(H5–H4β)_ = 8.9 Hz, ^3^*J*_(H5–H4α)_ = 7.4 Hz, HC5), 3.98 (dd, 1H, ^2^*J*_(HCH)_ = 13.8 Hz, ^3^*J*_(HCC–H3)_ = 3.9 Hz, *H*CH–Thy), 3.81 (s, 3H, C(O)OC*H*_3_), 3.50 (dddd, 1H, ^3^*J*_(H3–CCH)_ = 9.1 Hz, ^3^*J*_(H3–H4α)_ = 7.4 Hz, ^3^*J*_(H3–CCH)_ = 3.9 Hz, ^3^*J*_(H3–H4β)_ = 2.6 Hz, HC3), 3.36 (dd, 1H, ^2^*J*_(HCH)_ = 13.8 Hz, ^3^*J*_(HCC–H3)_ = 9.1 Hz, HC*H*–Thy), 2.82 (ddd, 1H, ^2^*J*_(H4α–H4β)_ = 13.5 Hz, ^3^*J*_(H4α–H5)_ = 7.4 Hz, ^3^*J*_(H4α–H3)_ = 7.4 Hz, H_α_C4), 2.70 (s, 3H, CH_3_N), 2.39 (ddd, 1H, ^2^*J*_(H4β–H4α)_ = 13.5 Hz, ^3^*J*_(H4β–H5)_ = 8.9 Hz, ^3^*J*_(H4β–H3)_ = 2.6 Hz, H_β_C4), 1.94 (s, 3H, CH_3_); ^13^C-NMR (150 MHz, CDCl_3_) δ: 172.32 (*C*(O)OCH_3_), 164.01 (C4′), 150.92 (C2′), 142.15 (C5′), 109.93 (C6′), 76.24 (C5), 65.81 (C3), 52.59 (C(O)O*C*H_3_), 50.00 (CH_2_–Thy), 45.72 (CH_3_N), 34.73 (C4), 12.21 (CH_3_). Anal. Calcd for C_12_H_17_N_3_O_5_: C, 50.88; H, 6.05; N, 14.83. Found: C, 50.94; H, 6.03; N, 14.95.

*Methyl cis-3-{[5-fluoro-2,4-dioxo-3,4–dihydropyrimidin-1(2H)-yl]metyl}-2-methylisoxazolidine-5-carboxylate* (*cis*-**14c**). Yield: 38%; a colorless amorphous solid; m.p. = 139–140 °C (crystallized from chloroform–methanol). IR (KBr, cm^−1^) *ν*_max_: 3441, 3166, 3055, 3028, 2958, 2923, 2850, 1725, 1691, 1658, 1470, 1436, 1242, 1074; ^1^H-NMR (600 MHz, CDCl_3_) δ: 9.81 (br s, 1H, NH), 7.43 (d, 1H, ^3^*J* = 5.8 Hz), 4.75 (dd, 1H, ^3^*J*_(H5–H4α)_ = 9.9 Hz, ^3^*J*_(H5–H4β)_ = 5.1 Hz, HC5), 3.93 (dd, 1H, ^2^*J*_(HCH)_ = 13.5 Hz, ^3^*J*_(HCC–H3)_ = 3.3 Hz, *H*CH–FUra), 3.80 (s, 3H, C(O)OCH_3_), 3.55 (dd, 1H, ^2^*J*_(HCH)_ = 13.5 Hz, ^3^*J*_(HCC–H3)_ = 9.5 Hz, HC*H*–FUra), 3.48 (dddd, 1H, ^3^*J*_(H3–CCH)_ = 9.5 Hz, ^3^*J*_(H3–H4α)_ = 7.9 Hz, ^3^*J*_(H3–CCH)_ = 3.3 Hz, ^3^*J*_(H3–H4β)_ = 2.0 Hz, HC3), 2.87 (ddd, 1H, ^2^*J*_(H4α–H4β)_ = 13.4 Hz, ^3^*J*_(H4α–H5)_ = 9.9 Hz, ^3^*J*_(H4α–H3)_ = 7.9 Hz, H_α_C4), 2.64 (s, 3H, CH_3_N), 2.19 (ddd, 1H, ^2^*J*_(H4β–H4α)_ = 13.4 Hz, ^3^*J*_(H4β–H5)_ = 5.1 Hz, ^3^*J*_(H4β–H3)_ = 2.0 Hz, H_β_C4); ^13^C-NMR (150 MHz, CDCl_3_) δ: 171.42 (*C*(O)OCH_3_), 157.48 (d, ^2^*J*_(CCF)_ = 25.8 Hz, C4′), 149.96 (C2′), 139.83 (d, ^1^*J*_(CF)_ = 234.2 Hz, C5′), 131.13 (d, ^2^*J*_(CCF)_ = 32.8 Hz, C6′), 74.41 (C5), 65.37 (C3), 52.68 (C(O)O*C*H_3_), 50.57 (CH_2_–FUra), 44.27 (CH_3_N), 34.61 (C4). Anal. Calcd for C_11_H_14_FN_3_O_5_: C, 46.00; H, 4.91; N, 14.63. Found: C, 46.36; H, 4.62; N, 14.55.

*Methyl trans-3-{[5-fluoro-2,4-dioxo-3,4–dihydropyrimidin-1(2H)-yl]metyl}-2-methylisoxazolidine-5-carboxylate* (*trans*-**14c**). Yield: 31%; a colorless amorphous solid; m.p. = 179–180 °C (crystallized from chloroform–methanol). IR (KBr, cm^−1^) *ν*_max_: 3470, 3167, 3029, 2960, 2920, 2836, 1744, 1722, 1693, 1658, 1382, 1240, 1206, 1101, 907; ^1^H-NMR (600 MHz, CDCl_3_) δ: 8.29 (br s, 1H, NH), 7.47 (d, 1H, ^3^*J* = 5.7 Hz), 4.56 (dd, 1H, ^3^*J*_(H5–H4β)_ = 9.0 Hz, ^3^*J*_(H5–H4α)_ = 7.5 Hz, HC5), 4.03 (dd, 1H, ^2^*J*_(HCH)_ = 13.9 Hz, ^3^*J*_(HCC–H3)_ = 3.6 Hz, *H*CH–FUra), 3.83 (s, 3H, C(O)OCH_3_), 3.52 (dddd, 1H, ^3^*J*_(H3–CCH)_ = 9.5 Hz, ^3^*J*_(H3–H4α)_ = 7.5 Hz, ^3^*J*_(H3–CC*H*)_ = 3.6 Hz, ^3^*J*_(H3–H4β)_ = 2.2 Hz, HC3), 3.31 (dd, 1H, ^2^*J*_(HCH)_ = 13.9 Hz, ^3^*J*_(HCC–H3)_ = 9.5 Hz, HC*H*–FUra), 2.86 (ddd, 1H, ^2^*J*_(H4α–H4β)_ = 13.4 Hz, ^3^*J*_(H4α–H5)_ = 7.5 Hz, ^3^*J*_(H4α–H3)_ = 7.5 Hz, H_α_C4), 2.71 (s, 3H, CH_3_N), 2.39 (ddd, 1H, ^2^*J*_(H4β–H4α)_ = 13.4 Hz, ^3^*J*_(H4β–H5)_ = 9.0 Hz, ^3^*J*_(H4β–H3)_ = 2.2 Hz, H_β_C4); signals of *trans-***14c** were extracted from the ^13^C-NMR spectrum of a 60:40 mixture of *trans-***14c** and *cis*-**14c**); ^13^C-NMR (150 MHz, CDCl_3_) δ: 172.29 (*C*(O)OCH_3_), 157.09 (d, ^2^*J*_(CCF)_ = 26.2 Hz, C4′), 149.58 (C2′), 139.84 (d, ^1^*J*_(CF)_ = 235.6 Hz, C5′), 130.71 (d, ^2^*J*_(CCF)_ = 33.0 Hz, C6′), 76.35 (C5), 65.75 (C3), 52.69 (C(O)O*C*H_3_), 49.96 (CH_2_–FUra), 45.61 (CH_3_N), 34.42 (C4). Anal. Calcd for C_11_H_14_FN_3_O_5_ × 0.25 H_2_O: C, 45.29; H, 5.01; N, 14.40. Found: C, 45.55; H, 4.99; N, 14.12.

*Methyl cis-3-{[5-bromo-2,4-dioxo-3,4–dihydropyrimidin-1(2H)-yl]metyl}-2-methylisoxazolidine-5-carboxylate* (*cis*-**14d**). Yield: 3.5%; a colorless amorphous solid; m.p. = 62–64 °C (crystallized from chloroform–methanol). IR (KBr, cm^−1^) *ν*_max_: 3156, 3094, 3007, 2959, 2922, 2876, 2831, 1759, 1710, 1679, 1461, 1285, 1044, 924; ^1^H-NMR (600 MHz, CDCl_3_) δ: 8.67 (br s, 1H, NH), 7.74 (s, 1H), 4.76 (dd, 1H, ^3^*J*_(H5–H4α)_ = 9.9 Hz, ^3^*J*_(H5–H4β)_ = 5.0 Hz, HC5), 3.95 (dd, 1H, ^2^*J*_(HCH)_ = 13.9 Hz, ^3^*J*_(HCC–H3)_ = 3.8 Hz, *H*CH-BrUra), 3.83 (s, 3H, C(O)OCH_3_), 3.59 (dd, 1H, ^2^*J*_(HCH)_ = 13.9 Hz, ^3^*J*_(HCC–H3)_ = 9.6 Hz, HC*H*–BrUra), 3.47 (dddd, 1H, ^3^*J*_(H3–CCH)_ = 9.6 Hz, ^3^*J*_(H3–H4α)_ = 8.2 Hz, ^3^*J*_(H3–CCH)_ = 3.8 Hz, ^3^*J*_(H3–H4β)_ = 2.1 Hz, HC3), 2.88 (ddd, 1H, ^2^*J*_(H4α–H4β)_ = 13.4 Hz, ^3^*J*_(H4α–H5)_ = 9.9 Hz, ^3^*J*_(H4α–H3)_ = 8.2 Hz, H_α_C4), 2.66 (s, 3H, CH_3_N), 2.20 (ddd, 1H, ^2^*J*_(H4__β__–H4α__)_ = 13.4 Hz, ^3^*J*_(H4__β__–H5)_ = 5.0 Hz, ^3^*J*_(H4__β__–H3)_ = 2.1 Hz, H_β_C4); ^13^C-NMR (150 MHz, CDCl_3_) δ: 171.33 (*C*(O)OCH_3_), 159.27 (C4′), 150.21 (C2′), 146.01 (C5′), 95.52 (C6′), 74.44 (C5), 65.29 (C3), 52.74 (C(O)O*C*H_3_), 50.73 (CH_2_–BrUra), 44.33 (CH_3_N), 34.52 (C4). Anal. Calcd for C_11_H_14_BrN_3_O_5_: C, 37.95; H, 4.05; N, 12.07. Found: C, 37.95; H, 3.97; N, 11.90.

*Methyl trans-3-{[5-bromo-2,4-dioxo-3,4–dihydropyrimidin-1(2H)-yl]metyl}-2-methylisoxazolidine-5-carboxylate* (*trans*-**14d**). Yield: 15%; a colorless amorphous solid; m.p. = 185–186 °C (crystallized from chloroform–methanol). IR (KBr, cm^−1^) *ν*_max_: 3416, 3156, 3093, 3004, 2959, 2924, 2878, 2833, 1711, 1681, 1614, 1511, 1463, 1285, 1255, 1032; ^1^H-NMR (600 MHz, CDCl_3_) δ: 8.32 (br s, 1H, NH), 7.67 (s, 1H), 4.57 (dd, 1H, ^3^*J*_(H4β–H5)_ = 9.0 Hz, ^3^*J*_(H4α–H5)_ = 7.4 Hz, HC5), 4.04 (dd, 1H, ^2^*J*_(HCH)_ = 13.9 Hz, ^3^*J*_(HCC–H3)_ = 3.7 Hz, *H*CH–BrUra), 3.83 (s, 3H, C(O)OCH_3_), 3.50 (dddd, 1H, ^3^*J*_(HCC–H3)_ = 9.7 Hz, ^3^*J*_(H4α–H3)_ = 7.4 Hz, ^3^*J*_(HCC–H3)_ = 3.7 Hz, ^3^*J*_(H4β–H3)_ = 2.0 Hz, HC3), 3.33 (dd, 1H, ^2^*J*_(HCH)_ = 13.9 Hz, ^3^*J*_(HCC–H3)_ = 9.7 Hz, HC*H*–BrUra), 2.87 (ddd, 1H, ^2^*J*_(H4α–H4β)_ = 13.5 Hz, ^3^*J*_(H4__α__–H5)_ = 7.4 Hz, ^3^*J*_(H4__α__–H3)_ = 7.4 Hz, H_α_C4), 2.70 (s, 3H, CH_3_N), 2.38 (ddd, 1H, ^2^*J*_(H4β–H4α)_ = 13.5 Hz, ^3^*J*_(H4__β__–H5)_ = 9.0 Hz, ^3^*J*_(H4__β__–H3)_ = 2.0 Hz, H_β_C4); ^13^C-NMR (150 MHz, CDCl_3_) δ: 172.28 (*C*(O)OCH_3_), 159.10 (C4′), 150.07 (C2′), 145.66 (C5′), 95.68 (C6′), 76.34 (C5), 65.61 (C3), 52.67 (C(O)O*C*H_3_), 50.10 (CH_2_–BrUra), 45.65 (CH_3_N), 34.33 (C4). Anal. Calcd for C_11_H_14_BrN_3_O_5_: C, 37.95; H, 4.05; N, 12.07. Found: C, 38.03; H, 3.91; N, 11.78.

*Methyl cis-3-{[5-chloro-2,4-dioxo-3,4–dihydropyrimidin-1(2H)-yl]metyl}-2-methylisoxazolidine-5-carboxylate* (*cis*-**14e**). Yield: 5.7%; a colorless amorphous solid; m.p. = 129–131 °C (crystallized from chloroform–methanol). IR (KBr, cm^−1^) *ν*_max_: 3473, 3161, 3036, 2926, 2824, 1748, 1717, 1666, 1627, 1442, 1424, 1342, 1215, 1179, 1049, 1007, 902, 719; ^1^H-NMR (600 MHz, CDCl_3_) δ: 9.32 (br s, 1H, NH), 7.63 (s, 1H), 4.76 (dd, 1H, ^3^*J*_(H5–H4α)_ = 9.9 Hz, ^3^*J*_(H5–H4β)_ = 5.0 Hz, HC5), 3.95 (dd, 1H, ^2^*J*_(HCH)_ = 13.9 Hz, ^3^*J*_(HCC–H3)_ = 3.8 Hz, *H*CH–ClUra), 3.82 (s, 3H, C(O)OCH_3_), 3.58 (dd, 1H, ^2^*J*_(HCH)_ = 13.9 Hz, ^3^*J*_(HCC–H3)_ = 9.5 Hz, HC*H*–ClUra), 3.48 (dddd, 1H, ^3^*J*_(H3–CCH)_ = 9.5 Hz, ^3^*J*_(H3–H4α)_ = 8.2 Hz, ^3^*J*_(H3–CCH)_ = 3.8 Hz, ^3^*J*_(H3–H4β)_ = 2.2 Hz, HC3), 2.88 (ddd, 1H, ^2^*J*_(H4α–H4β)_ = 13.5 Hz, ^3^*J*_(H4α–H5)_ = 9.9 Hz, ^3^*J*_(H4α–H3)_ = 8.2 Hz, H_α_C4), 2.65 (s, 3H, CH_3_N), 2.20 (ddd, 1H, ^2^*J*_(H4β–H4α)_ = 13.5 Hz, ^3^*J*_(H4β–H5)_ = 5.0 Hz, ^3^*J*_(H4β–H3)_ = 2.2 Hz, H_β_C4); ^13^C-NMR (150 MHz, CDCl_3_) δ: 171.37 (*C*(O)OCH_3_), 159.55 (C4′), 150.34 (C2′), 143.53 (C5′), 107.94 (C6′), 74.43 (C5), 65.28 (C3), 52.69 (C(O)O*C*H_3_), 50.71 (CH_2_–ClUra), 44.30 (CH_3_N), 34.57 (C4). Anal. Calcd for C_11_H_14_ClN_3_O_5_: C, 43.50; H, 4.65; N, 13.84; Found: C, 43.67; H, 4.75; N, 14.00.

*Methyl trans-3-{[5-chloro-2,4-dioxo-3,4–dihydropyrimidin-1(2H)-yl]metyl}-2-methylisoxazolidine-5-carboxylate* (*trans*-**14e**). Yield: 5.0%; a colorless amorphous solid; m.p. = 165–167 °C (crystallized from chloroform–methanol). IR (KBr, cm^−1^) *ν*_max_: 3453, 3165, 3029, 2952, 2843, 1756, 1697, 1678, 1628, 1468, 1432, 1365, 1209, 1181, 1056, 1028, 903, 752; ^1^H-NMR (600 MHz, CDCl_3_) δ: 8.34 (br s, 1H, NH), 7.57 (s, 1H), 4.56 (dd, 1H, ^3^*J*_(H5–H4β)_ = 9.0 Hz, ^3^*J*_(H5–H4α)_ = 7.5 Hz, HC5), 4.05 (dd, 1H, ^2^*J*_(HCH)_ = 13.9 Hz, ^3^*J*_(HCC–H3)_ = 3.7 Hz, *H*CH–ClUra), 3.83 (s, 3H, C(O)OCH_3_), 3.50 (dddd, 1H, ^3^*J*_(H3–CCH)_ = 9.7 Hz, ^3^*J*_(H3–H4α)_ = 7.5 Hz, ^3^*J*_(H3–CCH)_ = 3.7 Hz, ^3^*J*_(H3–H4β)_ = 2.0 Hz, HC3), 3.32 (dd, 1H, ^2^*J*_(HCH)_ = 13.9 Hz, ^3^*J*_(HCC–H3)_ = 9.7 Hz, HC*H*–ClUra), 2.87 (ddd, 1H, ^2^*J*_(H4α–H4β)_ = 13.5 Hz, ^3^*J*_(H4α–H5)_ = 7.5 Hz, ^3^*J*_(H4α–H3)_ = 7.5 Hz, H_α_C4), 2.70 (s, 3H, CH_3_N), 2.38 (ddd, 1H, ^2^*J*_(H4β–H4α)_ = 13.5 Hz, ^3^*J*_(H4β–H5)_ = 9.0 Hz, ^3^*J*_(H4β–H3)_ = 2.0 Hz, H_β_C4); ^13^C-NMR (150 MHz, CDCl_3_) δ: 172.26 (*C*(O)OCH_3_), 158.89 (C4′), 149.74 (C2′), 143.14 (C5′), 108.06 (C6′), 76.36 (C5), 65.62 (C3), 52.65 (C(O)O*C*H_3_), 50.11 (CH_2_–ClUra), 45.64 (CH_3_N), 34.34 (C4). Anal. Calcd for C_11_H_14_ClN_3_O_5_: C, 43.50; H, 4.65; N, 13.84; Found: C, 43.66; H, 4.80; N, 13.90.

*Methyl cis-3-{[5-iodo-2,4-dioxo-3,4–dihydropyrimidin-1(2H)-yl]metyl}-2-methylisoxazolidine-5-carboxylate* (*cis*-**14f**). Yield: 2.7%; a colorless amorphous solid; m.p. = 189–191 °C (crystallized from ethyl acetate–hexane). IR (KBr, cm^−1^) *ν*_max_: 3407, 3144, 3089, 3002, 2918, 2828, 1759, 1709, 1667, 1606, 1427, 1335, 1246, 1209, 1035, 886, 621; ^1^H-NMR (600 MHz, CDCl_3_) δ: 7.79 (s, 1H), 4.76 (dd, 1H, ^3^*J*_(H5–H4α)_ = 9.8 Hz, ^3^*J*_(H5–H4β)_ = 5.3 Hz, HC5), 3.92 (dd, 1H, ^2^*J*_(HCH)_ = 13.9 Hz, ^3^*J*_(HCC–H3)_ = 3.8 Hz, *H*CH–IUra), 3.83 (s, 3H, C(O)OC*H*_3_), 3.59 (dd, 1H, ^2^*J*_(HCH)_ = 13.9 Hz, ^3^*J*_(HCC–H3)_ = 9.6 Hz, HC*H*–IUra), 3.46 (dddd, 1H, ^3^*J*_(H3–CCH)_ = 9.6 Hz, ^3^*J*_(H3–H4α)_ = 8.4 Hz, ^3^*J*_(H3–CCH)_ = 3.8 Hz, ^3^*J*_(H3–H4β)_ = 2.2 Hz, HC3), 2.87 (ddd, 1H, ^2^*J*_(H4α–H4β)_ = 13.6 Hz, ^3^*J*_(H4α–H5)_ = 9.8 Hz, ^3^*J*_(H4α–H3)_ = 8.4 Hz, H_α_C4), 2.64 (s, 3H, CH_3_N), 2.19 (ddd, 1H, ^2^*J*_(H4β–H4α)_ = 13.6 Hz, ^3^*J*_(H4β–H5)_ = 5.3 Hz, ^3^*J*_(H4β–H3)_ = 2.2 Hz, H_β_C4); ^13^C-NMR (150 MHz, CDCl_3_) δ: 171.32 (*C*(O)OCH_3_), 160.27 (C4′), 150.94 (C2′), 150.51 (C6′), 74.43 (C5), 66.62 (C3), 65.33 (C5′), 52.76 (C(O)O*C*H_3_), 50.73 (CH_2_–IUra), 44.36 (CH_3_N), 34.53 (C4). Anal. Calcd for C_11_H_14_IN_3_O_5_: C, 33.44; H, 3.57; N, 10.63. Found: C, 33.34; H, 3.62; N, 10.51.

*Methyl trans-3-{[5-iodo-2,4-dioxo-3,4–dihydropyrimidin-1(2H)-yl]metyl}-2-methylisoxazolidine-5-carboxylate* (*tran*-**14f**). Yield: 5.3%; a colorless amorphous solid; m.p. = 214–216 °C (crystallized from chloroform–methanol). IR (KBr, cm^−1^) *ν*_max_: 3453, 3148, 3002, 2962, 2829, 1680, 1607, 1460, 1431, 1298, 1253, 1080, 895, 816, 721, 622; ^1^H-NMR (600 MHz, CDCl_3_) δ: 7.75 (s, 1H), 4.56 (dd, 1H, ^3^*J*_(H5–H4β)_ = 9.0 Hz, ^3^*J*_(H5–H4α)_ = 7.5 Hz, HC5), 4.03 (dd, 1H, ^2^*J*_(HCH)_ = 13.9 Hz, ^3^*J*_(HCC–H3)_ = 3.7 Hz, *H*CH–IUra), 3.83 (s, 3H, C(O)OC*H*_3_), 3.55 (dddd, 1H, ^3^*J*_(H3–CCH)_ = 9.7 Hz, ^3^*J*_(H3–H4α)_ = 7.5 Hz, ^3^*J*_(H3–CCH)_ = 3.7 Hz, ^3^*J*_(H3–H4β)_ = 2.0 Hz, HC3), 3.34 (dd, 1H, ^2^*J*_(HCH)_ = 13.9 Hz, ^3^*J*_(HCC–H3)_ = 9.7 Hz, HC*H*–IUra), 2.86 (ddd, 1H, ^2^*J*_(H4α–H4β)_ = 13.4 Hz, ^3^*J*_(H4α–H5)_ = 7.5 Hz, ^3^*J*_(H4α–H3)_ = 7.5 Hz, H_α_C4), 2.70 (s, 3H, CH_3_N), 2.37 (ddd, 1H, ^2^*J*_(H4β–H4α)_ = 13.4 Hz, ^3^*J*_(H4β–H5)_ = 9.0 Hz, ^3^*J*_(H4β–H3)_ = 2.0 Hz, H_β_C4); ^13^C-NMR (150 MHz, CDCl_3_) δ: 172.28 (*C*(O)OCH_3_), 160.10 (C4′), 150.62 (C2′), 150.38 (C6′), 76.32 (C5), 66.78 (C3), 65.63 (C5′), 52.67 (C(O)O*C*H_3_), 50.05 (CH_2_–IUra), 45.66 (CH_3_N), 34.34 (C4). Anal. Calcd for C_11_H_14_IN_3_O_5_: C, 33.44; H, 3.57; N, 10.63. Found: C, 33.57; H, 3.45; N, 10.76.

*Methyl cis-3-{[2,4-dioxotetrahydropyrimidin-1(2H)-yl]metyl}-2-methylisoxazolidine-5-carboxylate* (*cis*-**14g**). Yield: 6.4%; a colorless amorphous solid; m.p. = 102–104 °C (crystallized from ethyl acetate–hexane). IR (KBr, cm^−1^) *ν*_max_: 3366, 3243, 3110, 2957, 2921, 1721, 1675, 1486, 1440, 1384, 1331, 1228, 1205, 1152, 1060, 801; ^1^H-NMR (600 MHz, CDCl_3_) δ: 5.61 (br s, 1H, NH), 4.61 (dd, 1H, ^3^*J*_(H5–H4α)_ = 9.5 Hz, ^3^*J*_(H5–H4β)_ = 5.4 Hz, HC5), 3.96 (dd, 1H, ^2^*J*_(HCH)_ = 13.7 Hz, ^3^*J*_(HCC–H3)_ = 4.8 Hz, *H*CH–dihydroUra), 3.87 (dd, 1H, ^2^*J*_(HCH)_ = 13.7 Hz, ^3^*J*_(HCC–H3)_ = 6.9 Hz, HC*H*–dihydroUra), 3.80 (s, 3H, C(O)OCH_3_), 3.41 (td, 2H, ^3^*J* = 7.0 Hz, ^2^*J* = 2.7 Hz, CH_2_C*H*_2_N), 3.10–3.00 (br m, 1H, HC3), 2.76–2.73 (m, 5H, C*H*_3_N and C(O)C*H*_2_CH_2_N), 2.67 (ddd, 1H, ^2^*J*_(H4α–H4β)_ = 12.9 Hz, ^3^*J*_(H4α–H5)_ = 9.5 Hz, ^3^*J*_(H4α–H3)_ = 7.8 Hz, H_α_C4), 2.45 (ddd, 1H, ^2^*J*_(H4β–H4α)_ = 12.9 Hz, ^3^*J*_(H4β–H3)_ = 6.6 Hz, ^3^*J*_(H4β–H5)_ = 5.4 Hz, H_β_C4); ^13^C-NMR (150 MHz, CDCl_3_) δ: 172.25 (*C*(O)OCH_3_), 169.61 (C4′), 154.65 (C2′), 74.19 (C5), 66.42 (C3), 52.36 (C(O)O*C*H_3_), 44.31 (CH_2_–dihydroUra), 40.97 (CH_3_N), 36.71 (C6′), 35.33 (C4), 31.74 (C5′). Anal. Calcd for C_11_H_17_N_3_O_5_: C, 48.70; H, 6.32; N, 15.49. Found: C, 48.91; H, 6.29; N, 15.33.

*Methyl trans-3-{[2,4-dioxotetrahydropyrimidin-1(2H)-yl]metyl}-2-methylisoxazolidine-5-carboxylate* (*trans*-**14g**). Yield: 8.1%; a colorless amorphous solid; m.p. = 73–75 °C (crystallized from ethyl acetate–hexane). IR (KBr, cm^−1^) *ν*_max_: 3369, 3241, 3113, 2959, 2923, 1724, 1679, 1486, 1440, 1387, 1228, 1154, 1098, 1010, 763; ^1^H-NMR (600 MHz, CDCl_3_) δ: 5.71 (br s, 1H, NH), 4.62 (dd, 1H, ^3^*J*_(H5–H4β)_ = 8.6 Hz, ^3^*J*_(H5–H4α)_ = 6.9 Hz, HC5), 3.91 (dd, 1H, ^2^*J*_(HCH)_ = 13.5 Hz, ^3^*J*_(HCC–H3)_ = 5.9 Hz, *H*CH–dihydroUra), 3.89 (dd, 1H, ^2^*J*_(HCH)_ = 13.5 Hz, ^3^*J*_(HCC–H3)_ = 7.3 Hz, HC*H*–dihydroUra), 3.78 (s, 3H, C(O)OCH_3_), 3.44 (td, 2H, ^3^*J* = 6.8 Hz, ^2^*J* = 2.8 Hz), 3.32 (dddd, 1H, ^3^*J*_(H3–CCH)_ = 7.3 Hz, ^3^*J*_(H3–H4α)_ = 6.9 Hz, ^3^*J*_(H3–CCH)_ = 5.9 Hz, ^3^*J*_(H3–H4β)_ = 4.9 Hz, HC3), 2.80 (s, 3H, CH_3_N), 2.77 (t, 2H, ^3^*J* = 6.8 Hz), 2.55 (ddd, 1H, ^2^*J*_(H4α–H4β)_ = 12.9 Hz, ^3^*J*_(H4α–H5)_ = 6.9 Hz, ^3^*J*_(H4α–H3)_ = 6.9 Hz, H_α_C4), 2.45 (ddd, 1H, ^2^*J*_(H4β–H4α)_ = 12.9 Hz, ^3^*J*_(H4β–H5)_ = 8.6 Hz, ^3^*J*_(H4β–H3)_ = 4.9 Hz, H_β_C4); ^13^C-NMR (150 MHz, CDCl_3_) δ: 172.54 (*C*(O)OCH_3_), 169.65 (C4′), 154.71 (C2′), 75.71 (C5), 65.62 (C3), 52.31 (C(O)O*C*H_3_), 45.31 (CH_2_–dihydroUra), 41.19 (CH_3_N), 36.14 (C6′), 35.39 (C4), 31.64 (C5′). Anal. Calcd for C_11_H_17_N_3_O_5_: C, 48.70; H, 6.32; N, 15.49. Found: C, 49.05; H, 6.46; N, 15.58.

*Methyl cis-3-[(1,3-dimethyl-2,6-dioxo-1,2,3,6-tetrahydro-9H-purin-9-yl)methyl]-2-methylisoxazolidine-5-carboxylate* (*cis*-**14h**). Yield: 23%; a colorless amorphous solid; m.p. = 139–141 °C (crystallized from chloroform–diethyl ether). IR (KBr, cm^−1^) *ν*_max_: 2999, 2941, 1738, 1702, 1660,1546, 1455, 1409, 1373, 1286, 1184, 1091, 724; ^1^H-NMR (600 MHz, CDCl_3_) δ: 7.71 (s, 1H, HC8′), 4.75 (dd, 1H, ^3^*J*_(H5–H4α)_ = 9.7 Hz, ^3^*J*_(H5–H4β)_ = 5.1 Hz, HC5), 4.38 (dd, 1H, ^2^*J*_(HCH)_ = 13.7 Hz, ^3^*J*_(HCC–H3)_ = 4.7 Hz, *H*CH–The), 4.29 (dd, 1H, ^2^*J*_(HCH)_ = 13.7 Hz, ^3^*J*_(HCC–H3)_ = 8.8 Hz, HC*H*–The), 3.83 (s, 3H, NCH_3_), 3.62 (s, 3H, C(O)OCH_3_), 3.60 (dddd, 1H, ^3^*J*_(H3–CCH)_ = 8.8 Hz, ^3^*J*_(H3–H4α)_ = 8.3 Hz, ^3^*J*_(H3–CCH)_ = 4.7 Hz, ^3^*J*_(H3–H4β)_ = 3.0 Hz, HC3), 3.44 (s, 3H, NCH_3_), 2.86 (ddd, 1H, ^2^*J*_(H4α–H4β)_ = 13.3 Hz, ^3^*J*_(H4α–H5)_ = 9.7 Hz, ^3^*J*_(H4α–H3)_ = 8.3 Hz, H_α_C4), 2.59 (s, 3H, CH_3_N), 2.25 (ddd, 1H, ^2^*J*_(H4β–H4α)_ = 13.3 Hz, ^3^*J*_(H4β–H5)_ = 5.1 Hz, ^3^*J*_(H4β–H3)_ = 3.0 Hz, H_β_C4); ^13^C-NMR (150 MHz, CDCl_3_) δ: 171.47 (*C*(O)OCH_3_), 155.44 (C2′), 151.64 (C6′), 149.16 (C5′), 142.72 (C8′), 106.43 (C4′), 74.46 (C5), 66.68 (C3), 52.65 (C(O)O*C*H_3_), 49.11 (CH_2_–The), 44.52 (CH_3_N), 34.77 (C4), 29.81 (NCH_3_), 27.96 (NCH_3_). Anal. Calcd for C_14_H_19_N_5_O_5_: C, 49.85; H, 5.68; N, 20.76. Found: C, 49.75; H, 5.58; N, 20.64.

*Methyl trans-3-[(1,3-dimethyl-2,6-dioxo-1,2,3,6-tetrahydro-9H-purin-9-yl)methyl]-2-methylisoxazolidine-5-carboxylate* (*trans*-**14h**). Yield: 13%; a colorless amorphous solid; m.p. = 91–92 °C (crystallized from chloroform–diethyl ether). IR (KBr, cm^−1^) *ν*_max_: 2953, 2884, 1738, 1697, 1649, 1545, 1430, 1406, 1286, 1203, 1024, 973, 745; ^1^H-NMR (200 MHz, CDCl_3_) δ: 7.66 (s, 1H, HC8′), 4.54 (dd, 1H, ^3^*J*_(H5–H4β)_ = 8.9 Hz, ^3^*J*_(H5–H4α)_ = 7.5 Hz, HC5), 4.43 (dd, 1H, ^2^*J*_(HCH)_ = 13.7 Hz, ^3^*J*_(HCC–H3)_ = 4.3 Hz, *H*CH–The), 4.12 (dd, 1H, ^2^*J*_(HCH)_ = 13.7 Hz, ^3^*J*_(HCC–H3)_ = 8.7 Hz, HC*H*–The), 3.78 (s, 3H, NCH_3_), 3.60 (s, 3H, C(O)OCH_3_), 3.55 (dddd, 1H, ^3^*J*_(H3–CCH)_ = 8.7 Hz, ^3^*J*_(H3–H4α)_ = 7.5 Hz, ^3^*J*_(H3–CCH)_ = 4.3 Hz, ^3^*J*_(H3–H4β)_ = 3.2 Hz, HC3), 3.42 (s, 3H, NCH_3_), 2.80 (ddd, 1H, ^2^*J*_(H4α–H4β__)_ = 13.3 Hz, ^3^*J*_(H4__α__–H5)_ = 7.5 Hz, ^3^*J*_(H4__α__–H3)_ = 7.5 Hz, H_α_C4), 2.62 (s, 3H, CH_3_N), 2.39 (ddd, 1H, ^2^*J*_(H4__β__–H4α__)_ = 13.3 Hz, ^3^*J*_(H4__β__–H5)_ = 8.9 Hz, ^3^*J*_(H4__β__–H3)_ = 3.2 Hz, H_β_C4); ^13^C-NMR (150 MHz, CDCl_3_) δ: 172.11 (*C*(O)OCH_3_), 155.44 (C2′), 151.60 (C6′), 149.02 (C5′), 142.51 (C8′), 106.46 (C4′), 76.08 (C5), 66.70 (C3), 52.55 (C(O)O*C*H_3_), 48.55 (CH_2_–The), 45.65 (CH_3_N), 34.99 (C4), 29.80 (NCH_3_), 27.96 (NCH_3_). Anal. Calcd for C_14_H_19_N_5_O_5_: C, 49.85; H, 5.68; N, 20.76. Found: C, 49.60; H, 5.71; N, 20.65.

*Methyl cis-3-[(6-amino-9H-purin-9-yl)methyl]-2-methylisoxazolidine-5-carboxylate* (*cis*-**14i**). NMR signals of *cis*-**14i** were extracted from the spectra of a 67:33 mixture of *cis*-**14i** and *trans*-**14i**. ^1^H-NMR (300 MHz, CDCl_3_) δ: 8.34 (s, 1H, HC2′), 7.97 (s, 1H, HC8′), 5.96 (br s, 2H, NH_2_), 4.72 (dd, 1H, ^3^*J*_(H5–H4α)_ = 9.7 Hz, ^3^*J*_(H5–H4β)_ = 5.1 Hz, HC5), 4.26–4.23 (m, 2H, CH_2_–Ade), 3.78 (s, 3H, C(O)OC*H*_3_), 3.54–3.47 (m, 1H, HC3), 2.83 (ddd, 1H, ^2^*J*_(H4α–H4β)_ = 13.4 Hz, ^3^*J*_(H4α–H5)_ = 9.7 Hz, ^3^*J*_(H4α–H3)_ = 8.2 Hz, H_α_C4), 2.58 (s, 3H, CH_3_N), 2.25 (ddd, 1H, ^2^*J*_(H4β–H4α)_ = 13.4 Hz, ^3^*J*_(H4β–H5)_ = 5.1 Hz, ^3^*J*_(H4β–H3)_ = 3.3 Hz, H_β_C4); ^13^C-NMR (150 MHz, CDCl_3_) δ: 172.09 (*C*(O)OCH_3_), 155.49 (C2′), 152.83 (C6′), 150.05 (C4′), 141.72 (C8′), 119.33 (C5′), 76.04 (C5), 66.34 (C3), 52.52 (C(O)O*C*H_3_), 45.54 (CH_2_–Ade), 45.36 (CH_3_N), 35.16 (C4). Anal. Calcd for C_12_H_16_N_6_O_3_: C, 49.31; H, 5.52; N, 28.75. Found: C, 49.22; H, 5.71; N, 28.52 (obtained on a 67:33 mixture of *cis*-**14i** and *trans*-**14i**).

*Methyl trans-3-[(6-amino-9H-purin-9-yl)methyl]-2-methylisoxazolidine-5-carboxylate* (*trans*-**14i**). NMR signals of *trans*-**14i** were extracted from the spectra of a 68:32 mixture of *cis*-**14i** and *trans*-**14i** ^1^H-NMR (300 MHz, CDCl_3_) δ: 8.35 (s, 1H, HC2′), 7.95 (s, 1H, HC8′), 5.94 (br s, 2H, NH_2_), 4.49 (dd, 1H, ^3^*J*_(H4β–H5)_ = 8.7 Hz, ^3^*J*_(H4α–H5)_ = 7.5 Hz, HC5), 4.28 (dd, 1H, ^2^*J*_(HCH)_ = 14.1 Hz, ^3^*J*_(HCC–H3)_ = 4.5 Hz, *H*CH–Ade), 4.14 (dd, 1H, ^2^*J*_(HCH)_ = 14.1 Hz, ^3^J_(HCC–H3)_ = 8.0 Hz, HC*H*–Ade), 3.77 (s, 3H, C(O)OCH_3_), 3.50 (dddd, 1H, ^3^*J*_(HCC–H3)_ = 8.0 Hz, ^3^*J*_(H4α–H3)_ = 7.8 Hz, ^3^*J*_(HCC–H3)_ = 4.5 Hz, ^3^*J*_(H4β–H3)_ = 3.4 Hz, HC3), 2.77 (ddd, 1H, ^2^*J*_(H4α–H4β)_ = 13.2 Hz, ^3^*J*_(H4α–H5)_ = 7.5 Hz, ^3^*J*_(H4α–H3)_ = 7.8 Hz, H_α_C4), 2.64 (s, 3H, CH_3_N), 2.30 (ddd, 1H, ^2^*J*_(H4β–H4α)_ = 13.2 Hz, ^3^*J*_(H4β–H5)_ = 8.7 Hz, ^3^*J*_(H4β–H3)_ = 3.4 Hz, H_β_C4); ^13^C-NMR (150 MHz, CDCl_3_) δ: 171.39 (*C*(O)OCH_3_), 155.42 (C2′), 152.65 (C6′), 149.98 (C4′), 142.01 (C8′), 119.44 (C5′), 74.48 (C5), 66.11 (C3), 52.62 (C(O)O*C*H_3_), 45.88 (CH_2_–Ade), 44.41 (CH_3_N), 34.99 (C4). Anal. Calcd for C_12_H_16_N_6_O_3_: C, 49.31; H, 5.52; N, 28.75. Found: C, 49.17; H, 5.24; N, 28.39 (obtained on a 67:33 mixture of *cis*-**14i** and *trans*-**14i**).

### 3.3. General Procedure for the Synthesis of γ-Lactams trans-***15*** and cis-***15***

A solution of the respective isoxazolidines *cis*-**14** and *trans*-**14** (1.00 mmol) in methanol (3 mL) was stirred under atmospheric pressure of hydrogen over Pd/C (0.05 mmol) with Pd(OH)_2_/C (0.05 mmol) at room temperature for 1–2 days. The suspension was filtered through a layer of Celite. The solvent was removed in vacuo and crude products were chromatographed on silica gel columns with chloroform:methanol mixtures (50:1, 20:1 and 10:1, *v*/*v*) and then on HPLC using a X Bridge Prep, C8, 5 µm, OBD, 19 × 100 mm column and a water:methanol mixture (98:2, *v*/*v*) as an eluent to give γ-lactams *trans*-**15** and *cis*-**15**.

*trans-1-[(4-Hydroxy-1-methyl-5-oxopyrrolidin-2-yl)methyl]-5-methylpyrimidine-2,4(1H,3H)-dione* (*trans*-**15b**). Yield: 29%; a colorless amorphous solid; m.p. > 230 °C with decomposition (crystallized from methanol–chloroform). IR (KBr, cm^−1^) *ν*_max_: 3391, 3171, 3042, 2938, 2813, 2518, 1686, 1472, 1323, 1093, 944; ^1^H-NMR (600 MHz, CD_3_OD) δ: 7.38 (q, 1H, ^4^*J* = 1.0 Hz), 4.29 (dd, 1H, ^3^*J*_(H4–H3β)_ = 9.0 Hz, ^3^*J*_(H4–H3α)_ = 8.1 Hz, HC4), 4.01 (dd, 1H, ^2^*J*_(HCH)_ = 13.9 Hz, ^3^J_(HCC–H2)_ = 4.6 Hz, *H*CH–Thy), 3.92 (dddd, 1H, ^3^*J*_(H2–H3β)_ = 9.0 Hz, ^3^*J*_(H2–CCH)_ = 5.9 Hz, ^3^*J*_(H2–CCH)_ = 4.6 Hz, ^3^*J*_(H2–H3α)_ = 1.0 Hz, HC2), 3.84 (dd, 1H, ^2^*J*_(HCH)_ = 13.9 Hz, ^3^*J*_(HCC–H2)_ = 5.9 Hz, HC*H*–Thy), 2.92 (s, 3H, CH_3_N), 2.46 (ddd, 1H, ^2^*J*_(H3α–H3β)_ = 13.2 Hz, ^3^*J*_(H3α–H4)_ = 8.1 Hz, ^3^*J*_(H3α–H2)_ = 1.0 Hz, H_α_C3), 2.02 (ddd, 1H, ^2^*J*_(H3β–H3α)_ = 13.2 Hz, ^3^*J*_(H3β–H4)_ = 9.0 Hz, ^3^*J*_(H3β–H2)_ = 9.0 Hz, H_β_C3), 1.90 (d, 3H, ^4^*J* = 1.0 Hz, CH_3_); ^13^C-NMR (150 MHz, D_2_O) δ: 176.25 (*C*(O)), 166.91 (C4′), 152.55 (C2′), 142.71 (C5′), 111.53 (C6′), 68.26 (C4), 56.84 (CH_2_–Thy), 49.78 (C2), 31.81 (C3), 29.19 (CH_3_N), 11.32 (CH_3_). Anal. Calcd for C_11_H_15_N_3_O_4_: C, 52.17; H, 5.97; N, 16.59. Found: C, 52.25; H, 5.67; N, 16.59.

*cis-1-[(4-Hydroxy-1-methyl-5-oxopyrrolidin-2-yl)methyl]-5-methylpyrimidine-2,4(1H,3H)-dione* (*cis*-**15b**). Yield: 6.0%; a colorless amorphous solid; m.p. > 230 °C (crystallized from methanol–chloroform). IR (KBr, cm^−1^) *ν*_max_: 3552, 3454, 3389, 3315, 3175, 3042, 2952, 2930, 2832, 1692, 1661, 1469, 1127; ^1^H-NMR (600 MHz, D_2_O) δ: 7.36 (q, 1H, ^4^*J* = 1.1 Hz), 4.35 (dd, 1H, ^3^*J*_(H4–H3α)_ = 8.3 Hz, ^3^*J*_(H4–H3β)_ = 6.1 Hz, HC4), 4.19 (dd, 1H, ^2^*J*_(HCH)_ = 14.4 Hz, ^3^J_(HCC–H2)_ = 4.9 Hz, *H*CH–Thy), 3.94 (dddd, 1H, ^3^*J*_(H2–H3α)_ = 7.6 Hz, ^3^*J*_(H2–CCH)_ = 5.9 Hz, ^3^*J*_(H2–H3β)_ = 5.8 Hz, ^3^*J*_(H2–CCH)_ = 4.9 Hz, HC2), 3.90 (dd, 1H, ^2^*J*_(HCH)_ = 14.4 Hz, ^3^*J*_(HCC–H2)_ = 5.9 Hz, HC*H*–Thy), 2.88 (s, 3H, CH_3_N), 2.56 (ddd, 1H, ^2^*J*_(H3α–H3β)_ = 13.7 Hz, ^3^*J*_(H3α–H4)_ = 8.3 Hz, ^3^*J*_(H3α–H2)_ = 7.6 Hz, H_α_C3), 1.86 (q, 3H, ^4^*J* = 1.1 Hz, CH_3_), 1.68 (ddd, 1H, ^2^*J*_(H3β–H3α)_ = 13.7 Hz, ^3^J_(H3β__–H4)_ = 6.1 Hz, ^3^*J*_(H3__β__–H2)_ = 5.8 Hz, H_β_C3); ^13^C-NMR (150 MHz, D_2_O) δ: 176.48 (*C*(O)), 167.35 (C4′), 152.94 (C2′), 143.01 (C5′), 111.23 (C6′), 69.11 (C4), 56.82 (CH_2_–Thy), 50.26 (C2), 31.06 (C3), 28.82 (CH_3_N), 11.38 (CH_3_). Anal. Calcd for C_11_H_15_N_3_O_4_: C, 52.17; H, 5.97; N, 16.59. Found: C, 52.39; H, 5.55; N, 16.60.

*trans-1-[(4-Hydroxy-1-methyl-5-oxopyrrolidin-2-yl)metyl]dihydropyrimidine-2,4(1H,3H)-dione* (*trans*-**15g**). Yield: 4.0%; a colorless amorphous solid; m.p. > 230 °C with decomposition (crystallized from methanol–chloroform). IR (KBr, cm^−1^) *ν*_max_: 3226, 3097, 3048, 2959, 2873, 1708, 1670, 1488, 1462, 1255, 1140, 1048. ^1^H-NMR (600 MHz, CD_3_OD) δ: 4.54 (br s, 1H, NH), 4.42 (dd, 1H, ^3^*J*_(H4–H3β)_ = 8.7 Hz, ^3^*J*_(H4–H3α)_ = 8.1 Hz, HC4), 3.85 (dddd, 1H, ^3^*J*_(H2–H3β)_ = 8.7 Hz, ^3^*J*_(H2–CCH)_ = 6.5 Hz, ^3^*J*_(H2–CCH)_ = 4.8 Hz, ^3^*J*_(H2–H3α)_ = 1.6 Hz, HC2), 3.71 (dd, 1H, ^2^*J*_(HCH)_ = 14.1 Hz, ^3^*J*_(HCC–H2)_ = 4.8 Hz, *H*CH–dihydroUra), 3.58–3.49 (m, 2H), 3.46 (dd, 1H, ^2^*J*_(HCH)_ = 14.1 Hz, ^3^*J*_(HCC–H2)_ = 6.5 Hz, HC*H*–dihydroUra), 2.94 (s, 3H, CH_3_N), 2.72–2.63 (m, 2H), 2.43 (ddd, 1H, ^2^*J*_(H3__α__–H3β__)_ = 13.1 Hz, ^3^*J*_(H3__α__–H4)_ = 8.1 Hz, ^3^*J*_(H3__α__–H2)_ = 1.6 Hz, H_α_C3), 2.02 (ddd, 1H, ^2^*J*_(H3__β__–H3α__)_ = 13.1 Hz, ^3^*J*_(H3__β__–H4)_ = 8.7 Hz, ^3^*J*_(H3__β__–H2)_ = 8.7 Hz, H_β_C3); ^13^C-NMR (150 MHz, CD_3_OD) δ: 175.44 (*C*(O)), 171.24 (C4′), 154.34 (C2′), 68.17 (C4), 56.28 (CH_2_–dihydroUra), 48.60 (C2), 43.19 (C6′), 32.44 (C5′), 30.50 (CH_3_N), 27.81 (C3). Anal. Calcd for C_10_H_15_N_3_O_4_: C, 49.79; H, 6.27; N, 17.42. Found: C, 49.80; H, 6.26; N, 17.40.

*cis-1-[(4-Hydroxy-1-methyl-5-oxopyrrolidin-2-yl)metyl]dihydropyrimidine-2,4(1H,3H)-dione* (*cis*-**15g**). Yield: 23%; a colorless amorphous solid; m.p. > 230 °C with decomposition (crystallized from methanol–chloroform). IR (KBr, cm^−1^) *ν*_max_: 3298, 3243, 3047, 2959, 2822, 1674, 1461, 1239, 1062. ^1^H-NMR (600 MHz, CD_3_OD) δ: 4.63 (br s, 1H, NH), 4.26 (dd, 1H, ^3^*J*_(H4–H3α)_ = 8.2 Hz, ^3^*J*_(H4–H3β)_ = 7.0 Hz, HC4), 3.93 (dd, 1H, ^2^*J*_(HCH)_ = 14.3 Hz, ^3^*J*_(HCC–H2)_ = 4.6 Hz, *H*CH–dihydroUra), 3.79–3.75 (m, 1H, HC2), 3.56–3.53 (m, 2H), 3.45 (dd, 1H, ^2^*J*_(HCH)_ = 14.3 Hz, ^3^*J*_(HCC–H2)_ = 5.9 Hz, HC*H*–dihydroUra), 2.93 (s, 3H, CH_3_N), 2.70 (td, 2H, ^3^*J* = 6.6 Hz, ^2^*J* = 2.9 Hz), 2.56 (ddd, 1H, ^2^*J*_(H3α–H3β)_ = 13.4 Hz, ^3^*J*_(H3α–H4)_ = 8.2 Hz, ^3^*J*_(H3α–H2)_ = 7.5 Hz, H_α_C3), 1.73 (ddd, 1H, ^2^*J*_(H3β–H3α)_ = 13.4 Hz, ^3^*J*_(H3β–H4)_ = 7.0 Hz, ^3^*J*_(H3β–H2)_ = 6.5 Hz, H_β_C3); ^13^C-NMR (150 MHz, CD_3_OD) δ: 175.79 (*C*(O)), 171.29 (C4′), 154.26 (C2′), 68.99 (C4), 55.97 (*C*H_2_–dihydroUra), 49.14 (C2), 43.16 (C6′), 31.93 (C5′), 30.38 (CH_3_N), 27.50 (C3). Anal. Calcd for C_10_H_15_N_3_O_4_: C, 49.79; H, 6.27; N, 17.42. Found: C, 49.68; H, 6.17; N, 17.30.

*trans-9-[(4-Hydroxy-1-methyl-5-oxopyrrolidin-2-yl)methyl]-1,3-dimethyl-3,9-dihydro-1H-purine-2,6-dione* (*trans*-**15h**). Yield: 34%; a colorless amorphous solid; m.p. > 230 °C (crystallized from methanol–chloroform). IR (KBr, cm^−1^) *ν*_max_: 3345, 3103, 2923, 2853, 1709, 1680, 1665, 1546, 1469, 1407,1316, 1237, 1085, 761. ^1^H-NMR (600 MHz, CDCl_3_) δ: 7.52 (s, 1H), 4.67 (dd, 1H, ^2^*J*_(HCH)_ = 13.9 Hz, ^3^*J*_(HCC–H2)_ = 4.8 Hz, *H*CH–The), 4.21 (dd, 1H, ^2^*J*_(HCH)_ = 13.9 Hz, ^3^*J*_(HCC–H2)_ = 6.4 Hz, HC*H*–The), 4.16 (dd, 1H, ^3^*J*_(H4–H3α)_ = 8.6 Hz, ^3^*J*_(H4–H3β)_ = 8.6 Hz, HC4), 4.07–4.04 (m, 1H, HC2), 3.62 (s, 3H, CH_3_), 3.45 (s, 3H, CH_3_), 2.94 (s, 3H, CH_3_N), 2.38 (ddd, 1H, ^2^*J*_(H3α–H3β)_ = 13.4 Hz, ^3^*J*_(H3α–H4)_ = 8.6 Hz, ^3^*J*_(H3α–H2)_ = 8.0 Hz, H_α_C3), 2.14 (ddd, 1H, ^2^*J*_(H3β–H3α)_ = 13.4 Hz, ^3^*J*_(H3β–H2)_ = 9.0 Hz, ^3^*J*_(H3β–H4)_ = 8.6 Hz, H_β_C3); ^13^C-NMR (150 MHz, CDCl_3_) δ: 174.89 (*C*(O)), 155.35 (C2′), 151.48 (C6′), 149.23 (C4′), 141.20 (C8′), 106.92 (C5′), 67.79 (C4), 57.33 (CH_2_–The), 48.56 (C2), 31.70 (CH_3_), 29.89 (CH_3_N), 29.07 (C3), 28.09 (CH_3_). Anal. Calcd for C_13_H_17_N_5_O_4_ × 0.2 H_2_O: C, 50.22; H, 5.64; N, 22.53. Found: C, 50.06; H, 5.46; N, 22.40.

*cis-9-[(4-Hydroxy-1-methyl-5-oxopyrrolidin-2-yl)methyl]-1,3-dimethyl-3,9–dihydro-1H-purine-2,6-dione* (*cis*-**15h**). Yield: 26%; a colorless amorphous solid; m.p. > 230 °C (crystallized from methanol–chloroform). IR (KBr, cm^−1^) *ν*_max_: 3284, 3112, 2999, 2961, 2920, 1694, 1679, 1654, 1457, 1296, 1238, 1085, 749. ^1^H-NMR (600 MHz, CDCl_3_) δ: 7.65 (s, 1H), 4.83 (dd, 1H, ^2^*J*_(HCH)_ = 13.4 Hz, ^3^*J*_(HCC–H2)_ = 4.8 Hz, *H*CH–The), 4.31 (dd, 1H, ^3^*J*_(H4–H3α)_ = 7.6 Hz, ^3^*J*_(H4–H3β)_ = 4.0 Hz, HC4), 4.23 (dd, 1H, ^2^*J*_(HCH)_ = 13.4 Hz, ^3^*J*_(HCC–H2)_ = 7.9 Hz, HC*H*–The), 3.94 (dddd, 1H, ^3^*J*_(H2–CCH)_ = 7.9 Hz, ^3^*J*_(H2–H3α)_ = 7.6 Hz, ^3^*J*_(H2–H3β)_ = 4.0 Hz, ^3^*J*_(H2–CCH)_ = 4.8 Hz, HC2), 3.62 (s, 3H, CH_3_), 3.44 (s, 3H, CH_3_), 2.96 (s, 3H, CH_3_N), 2.38 (ddd, 1H, ^2^*J*_(H3α–H3β)_ = 13.8 Hz, ^3^*J*_(H3α–H4)_ = 7.6 Hz, ^3^*J*_(H3α–H2)_ = 7.6 Hz, H_α_C3), 1.76 (ddd, 1H, ^2^*J*_(H3β–H3α)_ = 13.8 Hz, ^3^*J*_(H3β–H4)_ = 4.0 Hz, ^3^*J*_(H3β–H2)_ = 4.0 Hz, H_β_C3); ^13^C-NMR (150 MHz, CDCl_3_) δ: 175.09 (*C*(O)), 155.33 (C2′), 151.55 (C6′), 149.36 (C4′), 141.63 (C8′), 106.83 (C5′), 69.26 (C4), 57.75 (CH_2_–The), 50.21 (C2), 31.31 (CH_3_), 29.89 (CH_3_N), 28.82 (C3), 28.06 (CH_3_). Anal. Calcd for C_13_H_17_N_5_O_4_: C, 50.81; H, 5.58; N, 22.79. Found: C, 50.73; H, 5.67; N, 22.96.

*trans-5-[(6-Amino-9H-purin-9-yl)methyl]-3-hydroxy-1-methylpyrrolidin-2-one* (*trans*-**15i**). Yield: 28%; a colorless amorphous solid; m.p. > 230 °C with decomposition (crystallized from water). IR (KBr, cm^−1^) *ν*_max_: 3319, 2955, 2923, 2853, 1687, 1603, 1483, 1326, 1244, 1132, 1000, 797. ^1^H-NMR (300 MHz, DMSO) δ: 8.17 (s, 1H, HC2′), 8.04 (s, 1H, HC8′), 7.23 (br s, 2H, NH_2_), 5.35 (d, 1H, *J* = 5.6 Hz, OH), 4.37 (dAB, 1H, ^2^*J*_AB_ = 14.6 Hz, ^3^*J*_(HCC–H2)_ = 3.8 Hz, *H*CH–Ade), 4.32 (dAB, 1H, ^2^*J*_AB_ = 14.6 Hz, ^3^*J*_(HCC–H2)_ = 4.5 Hz, HC*H*–Ade), 3.96–3.94 (m, 1H), 3.26–3.22 (m, 1H), 2.82 (s, 3H, CH_3_N), 2.30–2.23 (dd, 1H, ^2^*J*_(H3β–H3α)_ = 13.1 Hz, ^3^*J* = 8.3 Hz, H_α_C3), 1.82 (ddd, 1H, ^2^*J*_(H3β–H3α)_ = 13.1 Hz, ^3^*J* = 9.1 Hz, ^3^*J* = 9.0 Hz, H_β_C3); ^13^C-NMR (150 MHz, DMSO) δ: 174.34 (*C*(O)), 156.52 (C2′), 153.15 (C6′), 150.41 (C4′), 141.54 (C8′), 118.89 (C5′), 67.09 (C4), 56.52 (CH_2_–Ade), 44.56 (C2), 32.58 (C3), 28.52 (CH_3_N). Anal. Calcd for C_11_H_14_N_6_O_2_: C, 50.38; H, 5.38; N, 32.04. Found: C, 50.30; H, 5.20; N, 32.34.

*cis-5-[(6-Amino-9H-purin-9-yl)methyl]-3-hydroxy-1-methylpyrrolidin-2-one* (*cis*-**15i**). A colorless oil. IR (KBr, cm^−1^) *ν*_max_: 3469, 3341, 3173, 3071, 2959, 2922, 2852, 1745, 1601, 1511, 1473, 1303, 1270, 1038. NMR signals of *cis*-**15i** were extracted from the spectra of a 32:78 mixture of *trans*-**15i** and *cis*-**15i** ^1^H-NMR (300 MHz, DMSO-*d6*) δ: 8.14 (s, 1H, *H*C2′), 8.11 (s, 1H, HC8′), 7.20 (br s, 2H, NH_2_), 5.51 (d, 1H, *J* = 5.3 Hz, OH), 4.50 (dd, 1H, ^2^*J*_(HCH)_ = 14.3 Hz, ^3^*J*_(HCC–H2)_ = 4.7 Hz, *H*CH–Ade), 4.29 (dd, 1H, ^2^*J*_(HCH)_ = 14.3 Hz, ^3^*J*_(HCC–H2)_ = 6.1 Hz, HC*H*–Ade), 4.05–4.01 (m, 1H), 3.90–3.85 (m, 1H), 2.77 (s, 3H, CH_3_N), 2.27–2.23 (m, 1H, H_α_C3), 1.45 (dt, 1H ^2^*J* = 14.2 Hz, ^3^*J* = 7.0 Hz, H_β_C3); ^13^C-NMR (150 MHz, DMSO) δ: 175.09 (*C*(O)), 156.47 (C2′), 153.04 (C6′), 150.42 (C4′), 141.53 (C8′), 119.02 (C5′), 68.61 (C4), 55.78 (CH_2_–Ade), 45.84 (C2), 32.34 (C3), 28.57 (CH_3_N). Anal. Calcd for C_11_H_14_N_6_O_2_: C, 50.38; H, 5.38; N, 32.04. Found: C, 50.23; H, 5.08; N, 31.95 (obtained on a 40:60 mixture of *trans*-**15i** and *cis*-**15i**).

### 3.4. Synthesis of γ-Lactams cis-***15d*** and trans-***15d***

A solution of a 53:35:8:2 mixture of γ-lactams *trans*-**15a**, *cis*-**15a**, *trans*-**15g** and *trans*-**15g** (0.130 g, 0.54 mmol) and *N*-bromosuccinimide (0.106 g, 0.594 mmol) in DMF (4 mL) was stirred at room temperature for 24 h. The solvent was removed in vacuo and the crude product was purified on a silica gel column using chloroform-methanol (100:1, *v*/*v*) as an eluent. The respective fractions were subjected to chromatography on a X Bridge Prep, C8, 5 µm, OBD, 19 × 100 mm column using water/methanol (98:2, *v*/*v*) to give pure γ-lactams *trans*-**15d** (0.051 g, 30%) and *cis*-**15d** (0.016 g, 9.3%).

*trans-5-Bromo-1-[(4-hydroxy-1-methyl-5-oxopyrrolidin-2-yl)methyl]pyrimidine-2,4(1H,3H)-dione* (*trans*-**15d**). Yield: 30%; a colorless amorphous solid; m.p. = 199–200 °C with decomposition (crystallized from methanol–chloroform). IR (KBr, cm^−1^) *ν*_max_: 3381, 3123, 3022, 2908, 2818, 1681, 1502, 1461, 1212 1017, 939. ^1^H-NMR (600 MHz, D_2_O) δ: 8.00 (s, 1H), 4.41 (dd, 1H, ^3^*J*_(H4–H3β)_ = 8.9 Hz, ^3^*J*_(H4–H3α)_ = 8.7 Hz, HC4), 4.03 (dd, 1H, ^2^*J*_(HCH)_ = 13.4 Hz, ^3^*J*_(HCC–H2)_ = 4.9 Hz, *H*CH–BrUra), 4.00 (dddd, 1H, ^3^*J*_(H2–H3β)_ = 8.9 Hz, ^3^*J*_(H2–CCH)_ = 5.0 Hz, ^3^*J*_(H2–CCH)_ = 4.9Hz, ^3^*J*_(H2–H3α)_ = 5.7 Hz, HC2), 3.92(dd, 1H, ^2^*J*_(HCH)_ = 13.4 Hz, ^3^*J*_(HCC–H2)_ = 5.0 Hz, HC*H*–BrUra), 2.88 (s, 3H, CH_3_N), 2.46 (ddd, 1H, ^2^*J*_(H3α–H3β)_ = 13.5 Hz, ^3^*J*_(H3α–H4)_ = 8.7 Hz, ^3^*J*_(H3α–H2)_ = 5.7 Hz, H_α_C3), 2.07 (ddd, 1H, ^2^*J*_(H3β–H3α)_ = 13.5 Hz, ^3^J_(H3β–H4)_ = 8.9 Hz, ^3^*J*_(H3β–H2)_ = 8.9 Hz, H_β_C3); ^13^C-NMR (150 MHz, CD_3_OD) δ: 175.66 (C(O)), 160.46 (C4′), 151.08 (C2′), 145.19 (C5′), 95.73 (C6′), 68.04 (C4), 56.46 (CH_2_–BrUra), 49.47 (C2), 32.13 (C3), 28.26 (CH_3_N). Anal. Calcd for C_10_H_12_BrN_3_O_4_: C, 37.76; H, 3.80; N, 13.21. Found: C, 37.99; H, 3.85; N, 13.42.

*cis-5-Bromo-1-[(4-hydroxy-1-methyl-5-oxopyrrolidin-2-yl)methyl]pyrimidine-2,4(1H,3H)-dione* (*cis*-**15d**). Yield: 9.3%; a colorless amorphous solid; m.p. = 209–210 °C with decomposition (crystallized from methanol–chloroform). IR (KBr, cm^−1^) *ν*_max_: 3550, 3452, 3170, 3040, 2960, 2853, 2780, 1678, 1629, 1460, 1344, 1133. ^1^H-NMR (600 MHz, D_2_O) δ: 7.90 (s, 1H), 4.34 (dd, 1H, ^3^*J*_(H4–H3α)_ = 8.1 Hz, ^3^*J*_(H4–H3β)_ = 6.1 Hz, HC4), 4.21 (dd, 1H, ^2^*J*_(HCH)_ = 14.3 Hz, ^3^*J*_(HCC–H2)_ = 4.9 Hz, *H*CH–BrUra), 3.94 (dddd, 1H, ^3^*J*_(H2–CCH)_ = 9.8 Hz, ^3^*J*_(H2–H3α)_ = 7.8 Hz, ^3^*J*_(H2–H3β)_ = 7.1 Hz, ^3^*J*_(H2–CCH)_ = 4.9 Hz, HC2), 3.87 (dd, 1H, ^2^*J*_(HCH)_ = 14.3 Hz, ^3^*J*_(HCC–H2)_ = 9.8 Hz, HC*H*–BrUra), 2.88 (s, 3H, CH_3_N), 2.56 (ddd, 1H, ^2^*J*_(H3α–H3β)_ = 13.7 Hz, ^3^*J*_(H3α–H4)_ = 8.1 Hz, ^3^*J*_(H3α–H2)_ = 7.8 Hz, H_α_C3), 1.68 (ddd, 1H, ^2^*J*_(H3β–H3α)_ = 13.7 Hz, ^3^*J*_(H3β–H2)_ = 7.1 Hz, ^3^*J*_(H3β–H4)_ = 6.1 Hz, H_β_C3). ^13^C-NMR signals of *cis*-**15d** were extracted from the spectra of a 53:47 mixture of *cis*-**15d** and *trans*-**15d** ^13^C-NMR (150 MHz, CD_3_OD) δ: 175.44 (C(O)), 160.57 (C4′), 151.16 (C2′), 145.36 (C5′), 95.52 (C6′), 67.79 (C4), 56.14 (CH_2_–BrUra), 50.47 (C2), 31.48 (C3), 27.71 (CH_3_N). Anal. Calcd for C_10_H_12_BrN_3_O_4_: C, 37.76; H, 3.80; N, 13.21. Found: C, 38.00; H, 3.98; N, 13.50.

### 3.5. Synthesis of γ-Lactams cis-***15e*** and trans-***15e***

A solution of a 53:35:8:2 mixture of γ-lactams *trans*-**15a**, *cis*-**15a**, *trans*-**15g** and *trans*-**15g** (0.136 g, 0.61 mmol) and *N*-chlorosuccinimide (0.163 g, 1.22 mmol) in freshly distilled pyridine (12 mL) was stirred at 100 °C for 1 h. The solvent was removed in vacuo and the crude product was purified on a silica gel column using chloroform-methanol (100:1, *v*/*v*) as an eluent. The respective fractions were subjected to chromatography on an X Bridge Prep, C8, 5 µm, OBD, 19 × 100 mm column using water/methanol (98:2, *v*/*v*) to give pure γ-lactams *trans*-**15e** (0.01 g, 6%) and *cis*-**15d** (0.007 g, 4.2%).

*trans-5-Chloro-1-[(4-hydroxy-1-methyl-5-oxopyrrolidin-2-yl)methyl]pyrimidine-2,4(1H,3H)-dione* (*trans*-**15e**). Yield: 4.2%; a colorless amorphous solid; m.p. > 230 °C with decomposition (crystallized from methanol–chloroform). IR (KBr, cm^−1^) *ν*_max_: 3404, 3160, 3035, 2957, 2824, 1749, 1716, 1665, 1627, 1442, 1342, 1214, 1069, 878, 750. ^1^H-NMR (600 MHz, CD_3_OD) δ: 7.90 (s, 1H), 4.33 (dd, 1H, ^3^*J*_(H4–H3β)_ = 8.9 Hz, ^3^*J*_(H4–H3α)_ = 8.1 Hz, HC4), 4.03 (dd, 1H, ^2^*J*_(HCH)_ = 13.9 Hz, ^3^*J*_(HCC–H2)_ = 4.6 Hz, *H*CH–ClUra), 4.00 (dddd, 1H, ^3^*J*_(H2–H3β)_ = 8.9 Hz, ^3^*J*_(H2–CCH)_ = 6.2 Hz, ^3^*J*_(H2–CCH)_ = 4.6 Hz, ^3^*J*_(H2–H3α)_ = 0.8 Hz, HC2), 3.86 (dd, 1H, ^2^*J*_(HCH)_ = 13.9 Hz, ^3^*J*_(HCC–H2)_ = 6.2 Hz, HC*H*–ClUra), 2.93 (s, 3H, CH_3_N), 2.41 (ddd, 1H, ^2^*J*_(H3α–H3β)_ = 13.3 Hz, ^3^*J*_(H3α–H4)_ = 8.1 Hz, ^3^*J*_(H3α–H2)_ = 0.8 Hz, H_α_C3), 2.02 (ddd, 1H, ^2^*J*_(H3β–H3α)_ = 13.3 Hz, ^3^*J*_(H3β–H4)_ = 8.9 Hz, ^3^*J*_(H3β–H2)_ = 8.9 Hz, H_β_C3). ^13^C-NMR (150 MHz, CD_3_OD) δ: 175.47 (C(O)), 163.12 (C4′), 153.08 (C2′), 142.11 (C5′), 108.39 (C6′), 67.83 (C4), 56.23 (CH_2_–ClUra), 49.65 (C2), 32.02 (C3), 28.03 (CH_3_N). Anal. Calcd for C_10_H_12_ClN_3_O_4_: C, 43.89; H, 4.42; N, 15.35. Found: C, 43.78; H, 4.40; N, 15.37

*cis-5-Chloro-1-[(4-hydroxy-1-methyl-5-oxopyrrolidin-2-yl)methyl]pyrimidine-2,4(1H,3H)-dione* (*cis*-**15e**). Yield: 6.0%; a colorless amorphous solid; m.p. > 230 °C with decomposition (crystallized from methanol–chloroform). IR (KBr, cm^−1^) *ν*_max_: 3393, 3328, 3162, 3030, 2952, 2843, 1756, 1676, 1627, 1468, 1431, 1335, 1196, 903, 781. ^1^H-NMR (600 MHz, CD_3_OD) δ: 7.85 (s, 1H), 4.25–4.21 (m, 2H, HC4, *H*CH–ClUra), 3.90–3.86 (m, 1H, HC2), 3.84 (dd, 1H, ^2^*J*_(HCH)_ = 13.4Hz, ^3^*J*_(HCC–H2)_ = 6.5 Hz, HC*H*–ClUra), 2.92 (s, 3H, CH_3_N), 2.48 (ddd, 1H, ^2^*J*_(H3α–H3β)_ = 13.6 Hz, ^3^*J* = 7.8 Hz, ^3^*J* = 7.6 Hz, H_α_C3), 1.75 (ddd, 1H, ^2^*J*_(H3β–H3α)_ = 13.6 Hz, ^3^*J* = 5.6 Hz, ^3^*J* = 5.3 Hz, H_β_C3). ^13^C-NMR (150 MHz, CD_3_OD) δ: 175.69 (C(O)), 160.42 (C4′), 153.48 (C2′), 142.36 (C5′), 108.24 (C6′), 68.93 (C4), 56.27 (CH_2_–BrUra), 50.81 (C2), 31.58 (C3), 27.72 (CH_3_N). Anal. Calcd for C_10_H_12_ClN_3_O_4_: C, 43.89; H, 4.42; N, 15.35. Found: C, 43.80; H, 4.37; N, 15.30.

### 3.6. Antiviral Activity Assays

The compounds were evaluated against different herpesviruses, including herpes simplex virus type 1 (HSV-1) strain KOS, thymidine kinase-deficient (TK^−^) HSV-1 KOS strain resistant to ACV (ACV^r^), herpes simplex virus type 2 (HSV-2) strain G, adeno virus-2, human coronavirus, varicella-zoster virus (VZV) TK^+^ strain Oka, TK^–^ VZV strains 07-1, human cytomegalovirus (HCMV) strains AD-169 and Davis; para-influenza-3 virus, reovirus-1, Sindbis virus, Coxsackie virus B4, Punta Toro virus, respiratory syncytial virus (RSV), vesicular stomatitis virus, yellow fever virus, influenza A virus subtypes H1N1 (A/PR/8) and H3N2 (A/HK/7/87); and influenza B virus (B/HK/5/72). The antiviral assays were based on inhibition of virus-induced cytopathicity or plaque formation (for VZV) in human embryonic lung (HEL) fibroblasts, African green monkey kidney cells (Vero), human epithelial cervix carcinoma cells (HeLa), or Madin Darby canine kidney cells (MDCK). Confluent cell cultures in microtiter 96-well plates were inoculated with 100 CCID_50_ of virus (1 CCID_50_ being the virus dose to infect 50% of the cell cultures) or with 20 plaque forming units (PFU) (for VZV) and the cell cultures were incubated in the presence of varying concentrations of the test compounds. Viral cytopathicity or plaque formation (VZV) was recorded as soon as it reached completion in the control virus-infected cell cultures that were not treated with the test compounds. Antiviral activity was expressed as the EC_50_ or compound concentration required to reduce virus-induced cytopathicity or viral plaque formation by 50%. Cytotoxicity of the test compounds was expressed as the minimum cytotoxic concentration (MCC) or the compound concentration that caused a microscopically detectable alteration of cell morphology.

### 3.7. Cytostatic Activity against Immortalized Cell Lines

All assays were performed in 96-well microtiter plates. To each well was added (5−7.5) × 10^4^ tumor cells and a given amount of the test compound. The cells were allowed to proliferate at 37 °C in a humidified, CO_2_-controlled atmosphere. At the end of the incubation period, the cells were counted in a Coulter counter. The IC_50_ (50% inhibitory concentration) was defined as the concentration of the compound that inhibited cell proliferation by 50%.

## 4. Conclusions

A series of isoxazolidine analogues of homonucleosides *cis*-**14** and *trans*-**14** was synthesized by 1,3-dipolar cycloaddition of nucleobase-derived nitrones **13** and methyl acrylate. Based on ^1^H-NMR vicinal coupling constants the preferred ^2^*E* conformations were established for the isoxazolidine ring in both *cis*-**14** and *trans*-**14**. Hydrogenolytic transformation of isoxazolidines *cis*-**14** and *trans*-**14** into γ-lactams *trans*-**15** and *cis*-**15** was then performed to produce the respective γ-lactams containing thymine (*trans*-**15b**/*cis*-**15b**), dihydrouracil (*trans*-**15g**/*cis*-**15g**), theophylline (*trans*-**15h**/*cis*-**15h**) and adenine (*trans*-**15i**/*cis*-**15i**) as nucleobases by the application of the established protocol.

Since, during the hydrogenation of 5-halogenated uracil-containing isoxazolidine homonucleosides, the halogen atom was removed from a nucleobase skeleton, 5-bromouracil and 5-chlorouracil derivatives *trans*-**15d**/*cis*-**15d** (Br-Ura) and *trans*-**15e**/*cis*-**15e** (Cl-Ura) were obtained via the treatment of uracil-containing γ-lactams with NBS and NCS, respectively.

All obtained isoxazolidine and γ-lactam homonucleosides were evaluated against a broad-spectrum of DNA and RNA viruses and appeared inactive at concentrations up to 100 µM. Antiproliferative properties of the obtained isoxazolidines and γ-lactams were evaluated on nine cancerous cell lines and several of them exhibited moderate inhibitory activity, and at the same time they were inactive toward normal retina cells.

## Figures and Tables

**Table 1:** Isoxazolidines *trans*-**14** and *cis*-**14** obtained according to Scheme 2.

Entry	Nucleobase B	Ratio of *cis*-14:*trans*-14	Yield (%)
a	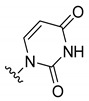	50:50 [45]	*cis*-**14a** − 26 ^a^;
			*cis*-**14a** + *trans*-**14a** − 50 ^b^;
			*trans*-**14a** − 19 ^a^
b	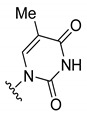	51:49	*cis*-**14b** − 7.4 ^a^;
			*cis*-**14b** + *trans*-**14b** − 66 ^b^;
			*trans*-**14b** − 17 ^a^
c	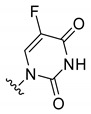	50:50	*cis*-**14c** − 38 ^a^;
			*cis*-**14c** + *trans*-**14c** − 4.4 ^b^;
			*trans*-**14c** − 31 ^a^
d	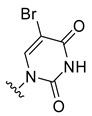	50:50	*cis*-**14d** − 3.5 ^a^;
			*cis*-**14d** + *trans*-**14d** − 63 ^b^;
			*trans*-**14d** − 15 ^a^
e	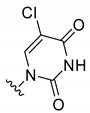	50:50	*cis*-**14e** − 5.7 ^a^;
			*cis*-**14e** + *trans*-**14e** − 75 ^b^;
			*trans*-**14e** − 5.0 ^a^
f	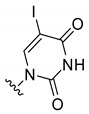	50:50	*cis*-**14f** − 2.7 ^a^;
			*cis*-**14f** + *trans*-**14f** − 75 ^b^;
			*trans*-**14f** − 5.3 ^a^
g	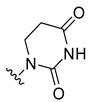	46:54	*cis*-**14g** − 6.4 ^a^;
			*cis*-**14g** + *trans*-**14g** − 57 ^b^;
			*trans*-**14g** − 8.1 ^a^
h	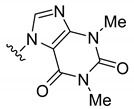	50:50	*cis*-**14h** − 23 ^a^;
			*cis*-**14h** + *trans*-**14h** − 62 ^b^;
			*trans*-**14h** − 13 ^a^
i	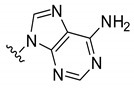	49:51	*cis*-**14i** + *trans*-**14i** − 64 ^b^

^a^ Yield of a pure diastereoisomer; ^b^ yield of a pure mixture of diastereoisomers;

**Table 2:** γ-Lactams *trans*-**15** and *cis*-**15** obtained according to Scheme 4.

Entry	Nucleobase (B)	Substrates	Products	Yields [%]
a	Ura	*cis*-**14a**	*trans*-**15g** [45]	80 ^a^
		*trans*-**14a**	*cis*-**15g**	85 ^a^
b	Thy	*cis*-**14b**:*trans*-**14b** (80:20)	*trans*-**15b**:*cis*-**15b** (80:20)	*trans*-**15b** − 29 ^a^ *cis*-**15b** + *trans*-**15b** – 24 ^b^ *cis-***15b** – 6.0 ^a^
c	F-Ura	*cis*-**14c**	*trans*-**15g**	70 ^a^
d	Br-Ura	*cis*-**14d**:*trans*-**14d** (60:40)	*trans*-**15a**:*cis*-**15a***:trans*-**15g**:*cis*-**15g** (53:35:8:2)	Inseparable (77%)
e	Cl-Ura	*cis*-**14e**:*trans*-**14e** (77:23)	*trans*-**15a**:*cis*-**15a***:trans*-**15g**:*cis*-**15g** (4:2:20:74 ^c^)	Inseparable (80%)
f	I-Ura	*cis*-**14f**:*trans*-**14f**	n.a.^c^	–
g	Dihydro-Ura	*cis*-**14g**:*trans*-**14g** (86:14)	*trans*-**15g**:*cis*-**15g** (81:19)	*trans-***15g** – 4.0 ^a^ *cis-***15g** + *trans-***15g** – 47 ^b^ *cis-***15g** – 23 ^a^
h	Theophylline	*cis*-**14h**	*trans*-**15h**	*trans-***15h** – 41 ^a^
		*cis*-**14h**:*trans*-**14h** (77:23)	*trans*-**15h**:*cis*-**15h** (80:20)	*trans-***15h** – 34 ^a^ *cis-***15h** + *trans-***15h** – 10 ^b^ *cis-***15h** – 26 ^a^
i	Ade	*cis*-**14i**:*trans*-**14i** (85:15)	*trans*-**15i**:*cis*-**15i** 87:13	*trans-***15i** – 28 ^a^ *cis-***15i** + *trans-***15i** – 7.3 ^b^
		*cis*-**14i**:*trans*-**14i** (42:58)	*trans*-**15i**:*cis*-**15i** 46:54	*cis-***15i** + *trans-***15i** – 55 ^b^

^a^ Yield of a pure diastereoisomer; ^b^ yield of a pure mixture of diastereoisomers; ^c^ not applied: since the dehalogenation of 5-halogenated uracil derivatives **14c**, **14d** and **14e** was observed, the hydrogenation of 5-iodouracil derivatives *cis*-**14f**/*trans*-**14f** was not performed.

**Table 3:** Inhibitory effect of isoxazolidines **14** and γ-lactams **15** against the proliferation of cancerous cells.

Base	Compound	IC_50_ (µM)				
		hTERT RPE-1	Capan-1	Hap1	HCT-116	NCI-H460
		Retina (Non Cancerous)	Pancreatic Adenocarcinoma	Chronic Myeloid Leukemia	Colorectal Carcinoma	Lung Carcinoma
Ura	*cis*-**14a**	>100	54.8	>100	>100	>100
Ura	*trans*-**14a**	>100	62.6	>100	>100	90.3
Thy	*cis*-**14b**	>100	55.2	63.0	>100	60.3
Thy	*trans*-**14b**	>100	51.3	>100	>100	>100
F-Ura	*cis*-**14c**	>100	>100	>100	>100	>100
F-Ura	*trans*-**14c**	>100	49.0	>100	>100	>100
Br-Ura	*cis*-**14d**	>100	95.0	>100	86.4	62.3
Br-Ura	*trans*-**14d**	>100	67.8	>100	>100	76.1
Cl-Ura	*cis*-**14e**	>100	51.2	44.2	26.7	48.8
Cl-Ura	*trans*-**14e**	>100	38.5	42.7	43.5	41.9
I-Ura	*cis*-**14f**	97.1	42.0	40.9	57.9	44.6
I-Ura	*trans*-**14f**	>100	41.4	47.0	53.5	41.1
dihydro-Ura	*trans*-**14g**	>100	38.3	58.1	61.0	48.6
Theo	*cis*-**14h**	>100	62.2	45.1	49.0	33.2
Theo	*trans*-**14h**	>100	43.2	53.6	60.9	51.2
Ade	*cis*-**14i**:*trans*-**14i** (99:1)	>100	73.5	>100	>100	>100
Ade	*cis*-**14i**:*trans*-**14i** (36:64)	>100	58.1	>100	68.4	85.7
Thy	*cis*-**15b**:*trans*-**15b** (64:36)	>100	58.0	>100	>100	50.0
Thy	*trans*-**15b**	>100	49.2	86.5	53.3	55.6
Br-Ura	*cis*-**15d**:*trans*-**15d** (53:47)	>100	52.1	49.9	90.0	49.4
Br-Ura	*trans*-**15d**	>100	24.8	58.5	>100	42.1
Cl-Ura	*cis*-**15e**	>100	28.0	51.5	58.5	28.6
Cl-Ura	*trans*-**15e**	>100	18.2	54.5	>100	47.7
dihydro-Ura	*cis*-**15g**	>100	41.3	56.3	>100	42.7
dihydro-Ura	*trans*-**15g**	>100	46.7	49.4	>100	69.3
Theo	*cis*-**15h**	>100	21.5	50.2	43.5	42.5
Theo	*trans*-**15h**	>100	47.2	>100	45.7	49.8
Ade	*trans*-**15i**	>100	53.7	47.8	65.7	54.7
Docetaxel	Docetaxel	25.0	0.95	1.19	0.25	0.89
Etoposide	Etoposide	0.23	0.15	0.04	1.03	1.35
Stauroporine	Stauroporine	0.25	0.66	3.55	0.09	11.50

## Supplementary Materials

The Supplementary Materials are available online.
