# Supplementary material for: Novel Isoxazolidine and γ-Lactam Analogues of Homonucleosides
Source: Molecules. 2019 Nov 6;24(22):4014. doi: 10.3390/molecules24224014 (PMC6891762; doi:10.3390/molecules24224014)
Supplement: Supplementary file 1 [file molecules-24-04014-s001.pdf]

SpinWorks 3: aw-il94-85-1H

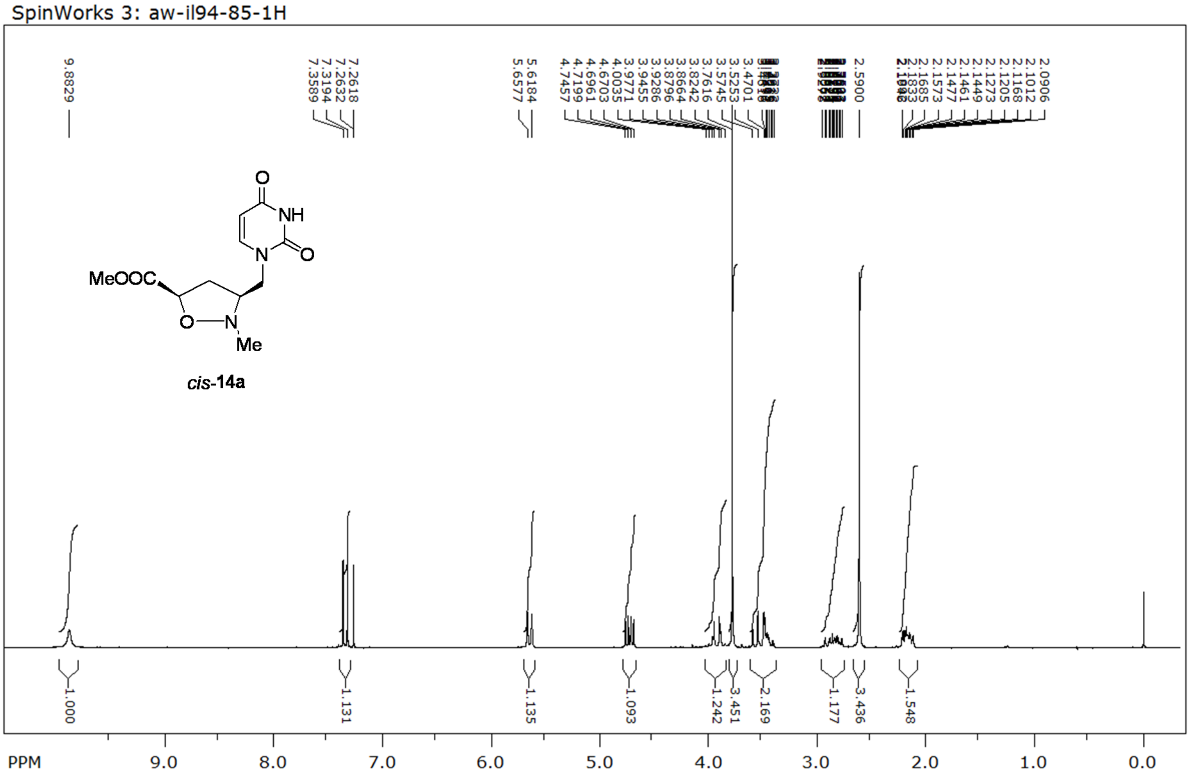

file: ...IZOKSAZOLIDYNY\jrg-1478-1H.fid\fid block# 1 expt: "s2pul"
transmitter freq.: 199.929728 MHz
time domain size: 32000 points
width: 4500.00 Hz = 22.5079 ppm = 0.140625 Hz/pt
number of scans: 16

freq. of 0 ppm: 199.928727 MHz
processed size: 65536 complex points
LB: 0.000 GF: 0.0000
Hz/cm: 86.879 ppm/cm: 0.43455

SpinWorks 3: jrg0497c

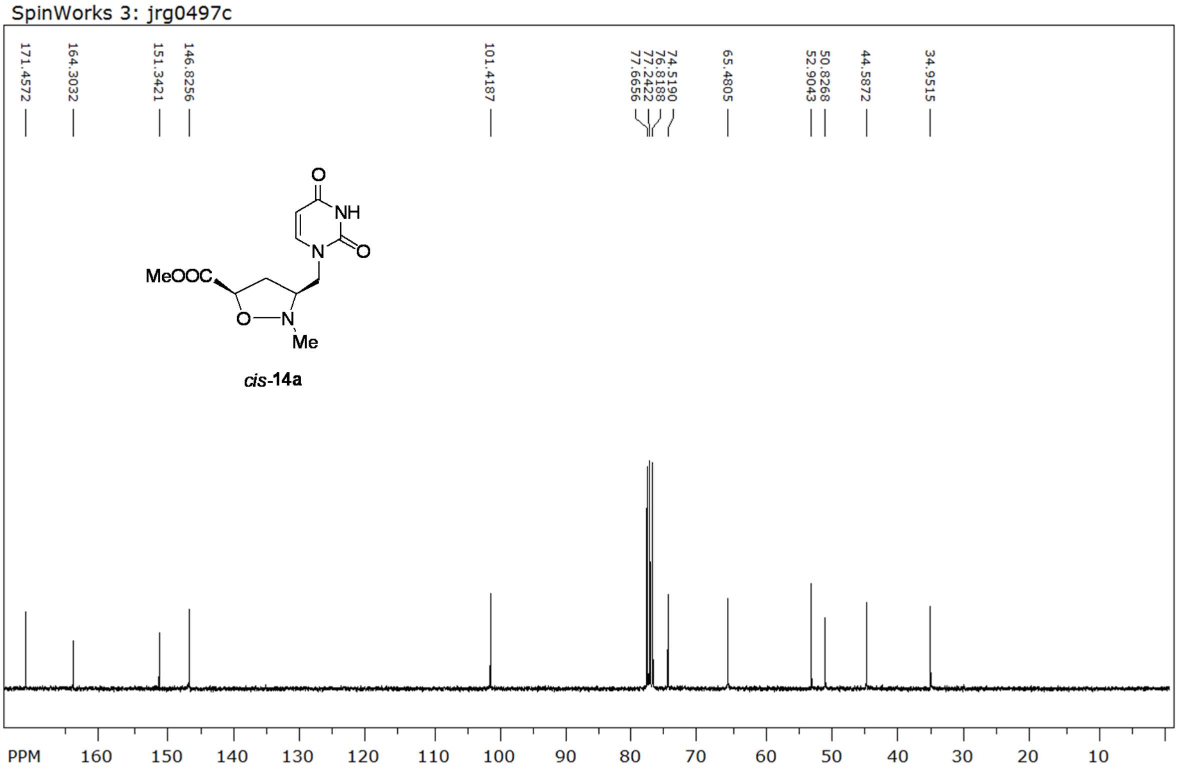

file: E:\IZOKSAZOLIDYNY\jrg0497c.fid\fid block# 1 expt: "s2pul"
transmitter freq.: 75.457729 MHz
time domain size: 68096 points
width: 18761.73 Hz = 248.6389 ppm = 0.275519 Hz/pt
number of scans: 1568

freq. of 0 ppm: 75.450188 MHz
processed size: 131072 complex points
LB: 0.000 GF: 0.0000
Hz/cm: 530.739 ppm/cm: 7.03359

SpinWorks 3: no title

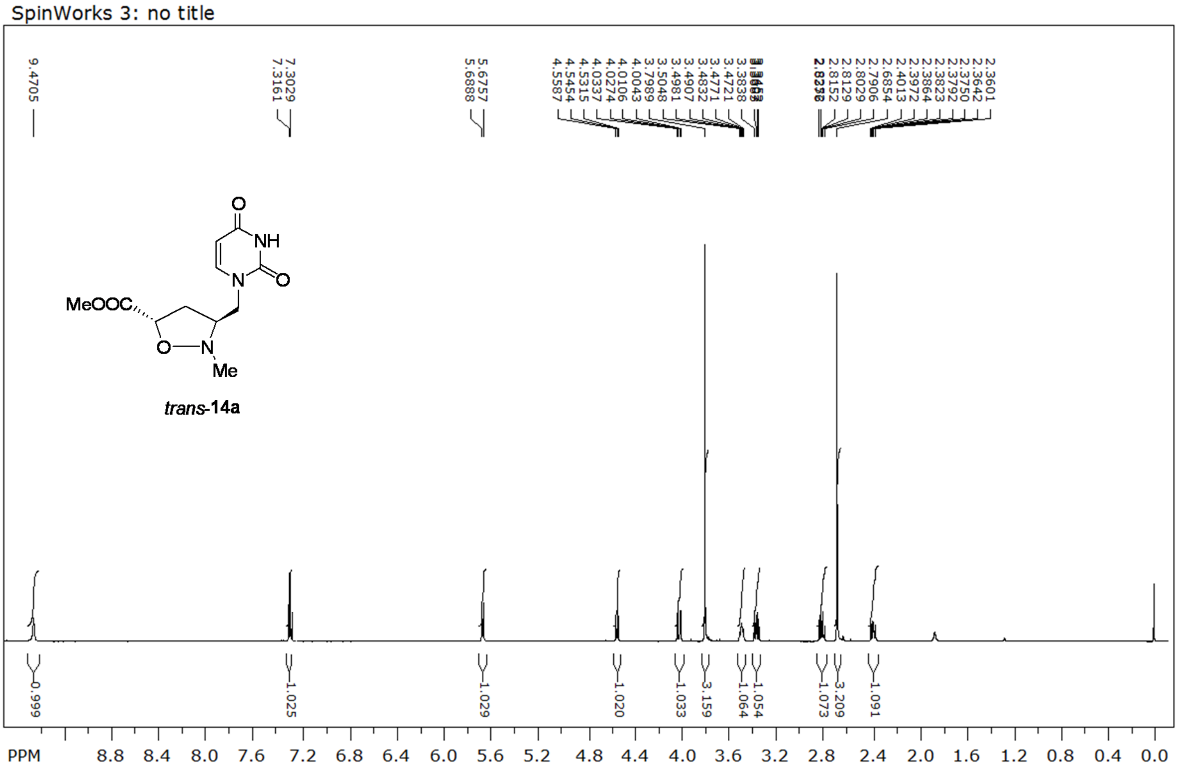

file: E:\IZOKSAZOLIDYNY\jrg-1490\10\fid expt: <zg30>
transmitter freq.: 600.263707 MHz
time domain size: 65536 points
width: 12335.53 Hz = 20.5502 ppm = 0.188225 Hz/pt
number of scans: 16

freq. of 0 ppm: 600.260000 MHz
processed size: 32768 complex points
LB: 0.000 GF: 0.0000
Hz/cm: 236.256 ppm/cm: 0.39359

SpinWorks 3: no title

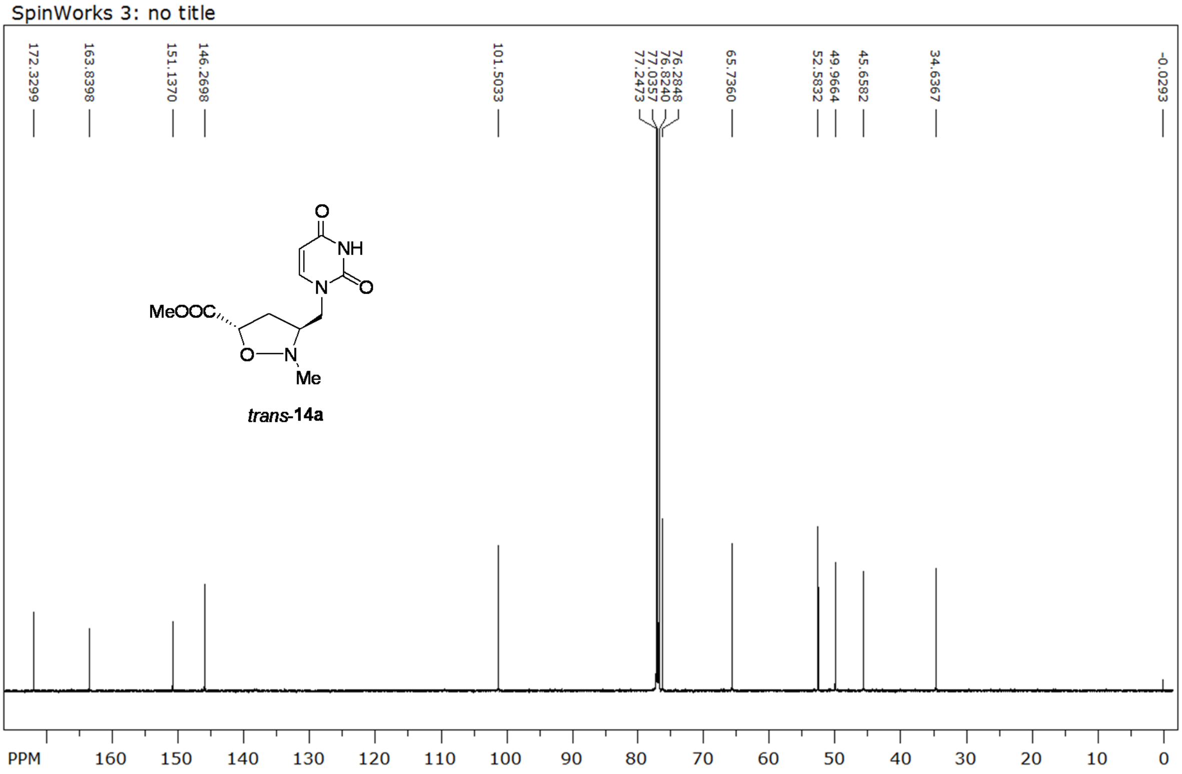

file: E:\IZOKSAZOLIDYNY\jrg-1490\11\fid expt: <zgpg30>
transmitter freq.: 150.950591 MHz
time domain size: 65536 points
width: 36057.69 Hz = 238.8708 ppm = 0.550197 Hz/pt
number of scans: 2048

freq. of 0 ppm: 150.935497 MHz
processed size: 32768 complex points
LB: 0.000 GF: 0.0000
Hz/cm: 1077.167 ppm/cm: 7.13589

SpinWorks 3: aw-il94-85-1H

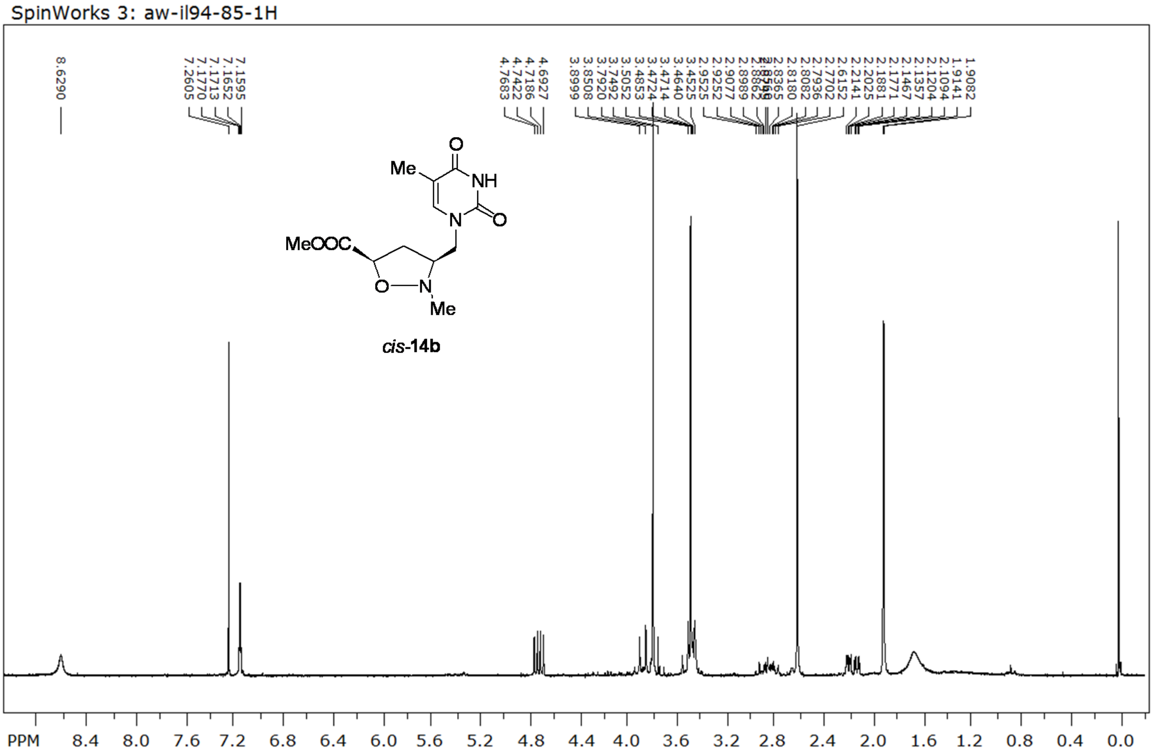

file: ...IZOKSAZOLIDYNY\jrg-1685-1H.fid\fid block# 1 expt: "s2pul"
transmitter freq.: 199.929728 MHz
time domain size: 32000 points
width: 4500.00 Hz = 22.5079 ppm = 0.140625 Hz/pt
number of scans: 16

freq. of 0 ppm: 199.928728 MHz
processed size: 65536 complex points
LB: 0.000 GF: 0.0000
Hz/cm: 74.496 ppm/cm: 0.37261

SpinWorks 3: no title

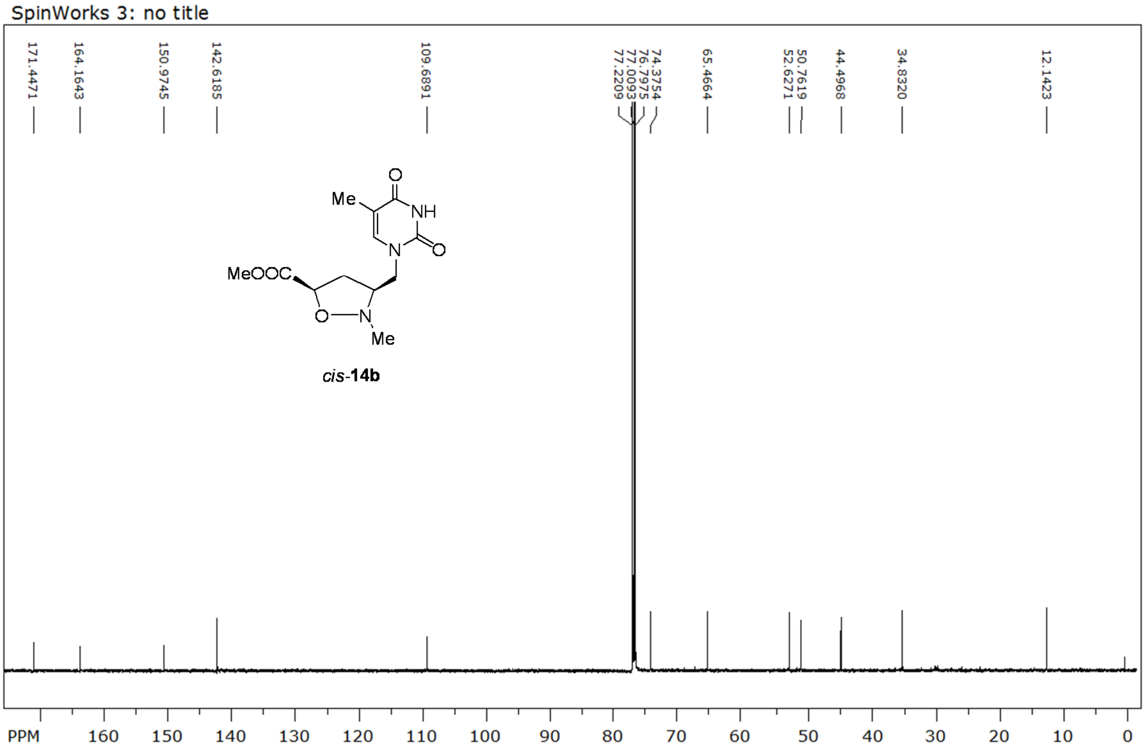

file: E:\IZOKSAZOLIDYNY\jrg-1692\11\fid expt: <zgpg30>
transmitter freq.: 150.950591 MHz
time domain size: 65536 points
width: 36057.69 Hz = 238.8708 ppm = 0.550197 Hz/pt
number of scans: 4000

freq. of 0 ppm: 150.935497 MHz
processed size: 32768 complex points
LB: 0.000 GF: 0.0000
Hz/cm: 1074.785 ppm/cm: 7.12011

SpinWorks 3: no title

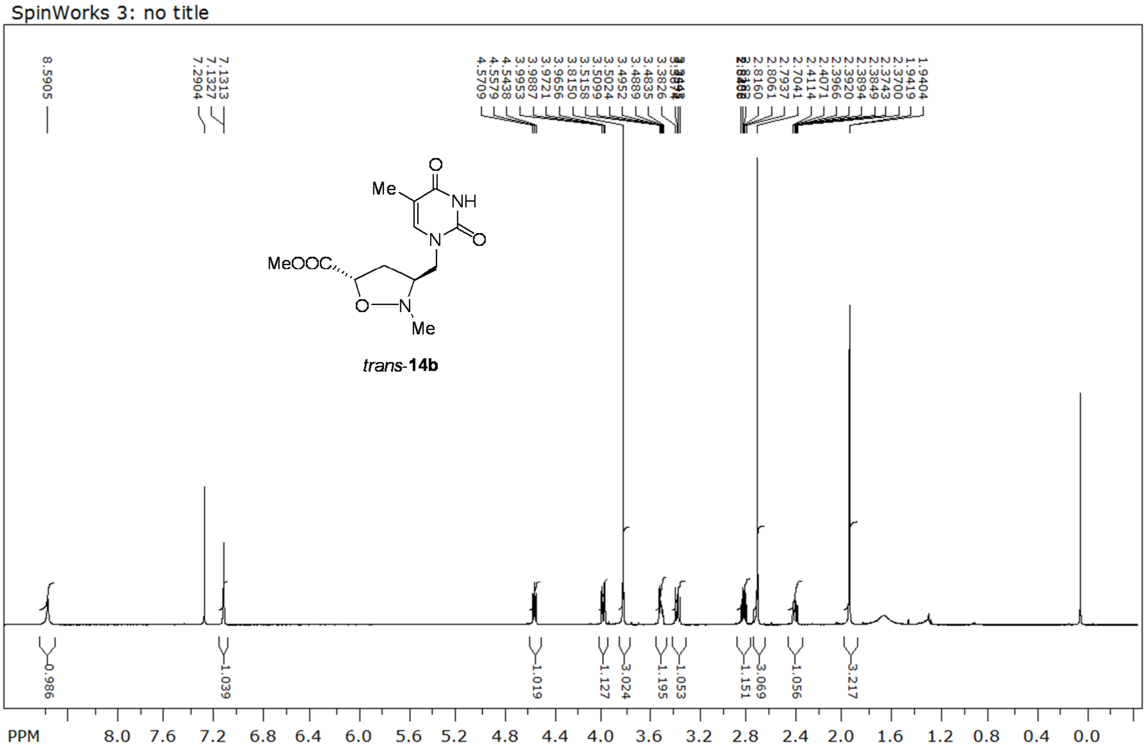

file: E:\IZOKSAZOLIDYNY\jrg-1691\10\fid expt: <zg30>
transmitter freq.: 600.263707 MHz
time domain size: 65536 points
width: 12335.53 Hz = 20.5502 ppm = 0.188225 Hz/pt
number of scans: 16

freq. of 0 ppm: 600.260000 MHz
processed size: 32768 complex points
LB: 0.000 GF: 0.0000
Hz/cm: 225.665 ppm/cm: 0.37594

SpinWorks 3: no title

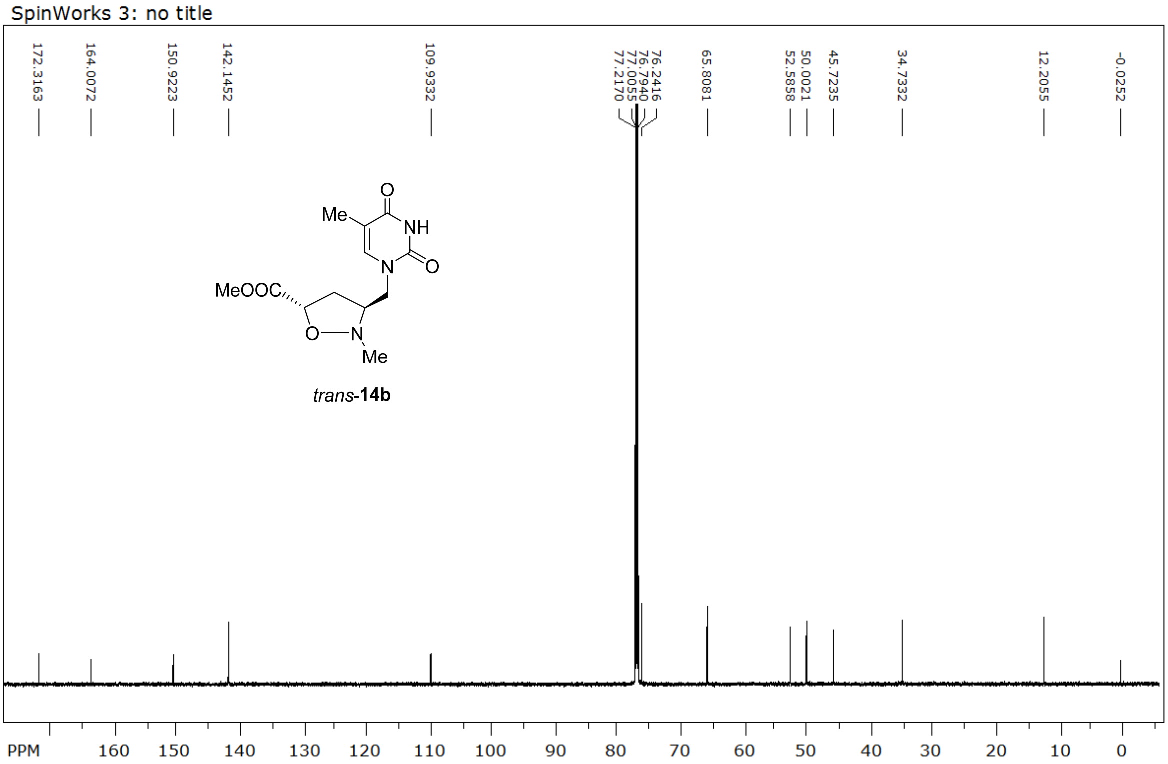

file: E:\IZOKSAZOLIDYNY\jrg-1691\11\fid expt: <zgpg30>
transmitter freq.: 150.950591 MHz
time domain size: 65536 points
width: 36057.69 Hz = 238.8708 ppm = 0.550197 Hz/pt
number of scans: 4000

freq. of 0 ppm: 150.935497 MHz
processed size: 32768 complex points
LB: 0.000 GF: 0.0000
Hz/cm: 1111.299 ppm/cm: 7.36201

SpinWorks 3: no title

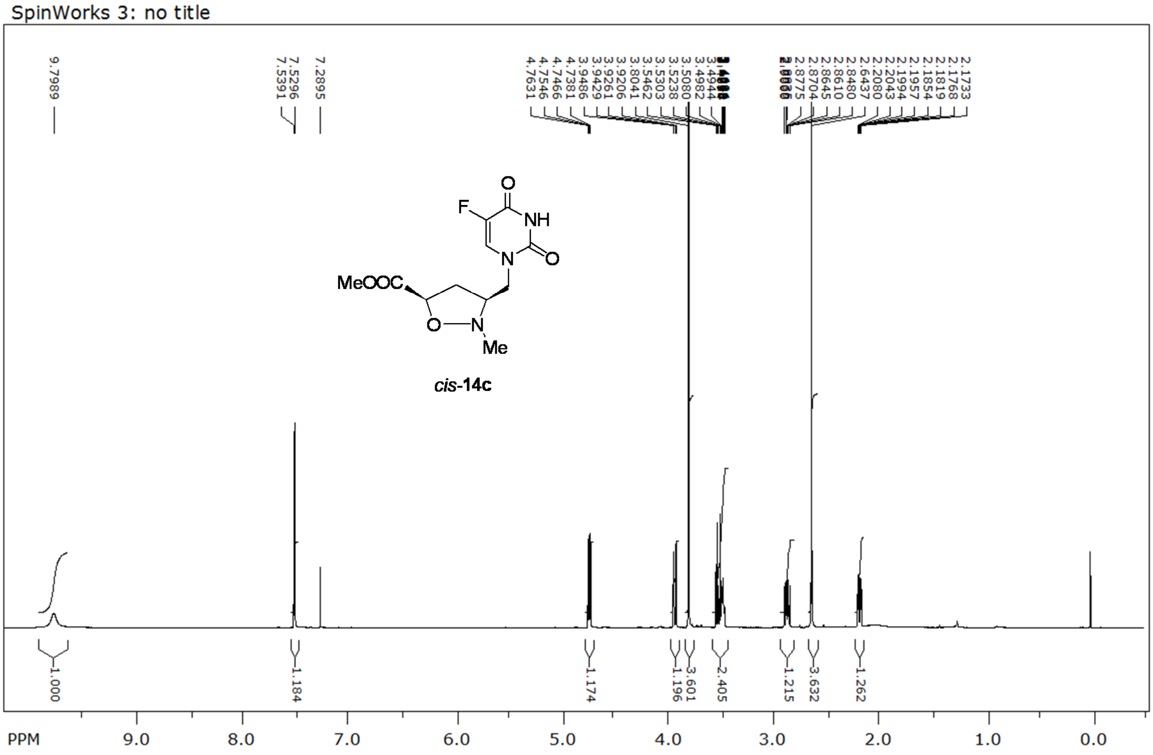

file: E:\IZOKSAZOLIDYNY\jrg-1708\10\fid expt: <zg30>
transmitter freq.: 600.263707 MHz
time domain size: 65536 points
width: 12335.53 Hz = 20.5502 ppm = 0.188225 Hz/pt
number of scans: 16

freq. of 0 ppm: 600.260000 MHz
processed size: 32768 complex points
LB: 0.000 GF: 0.0000
Hz/cm: 258.795 ppm/cm: 0.43114

SpinWorks 3: no title

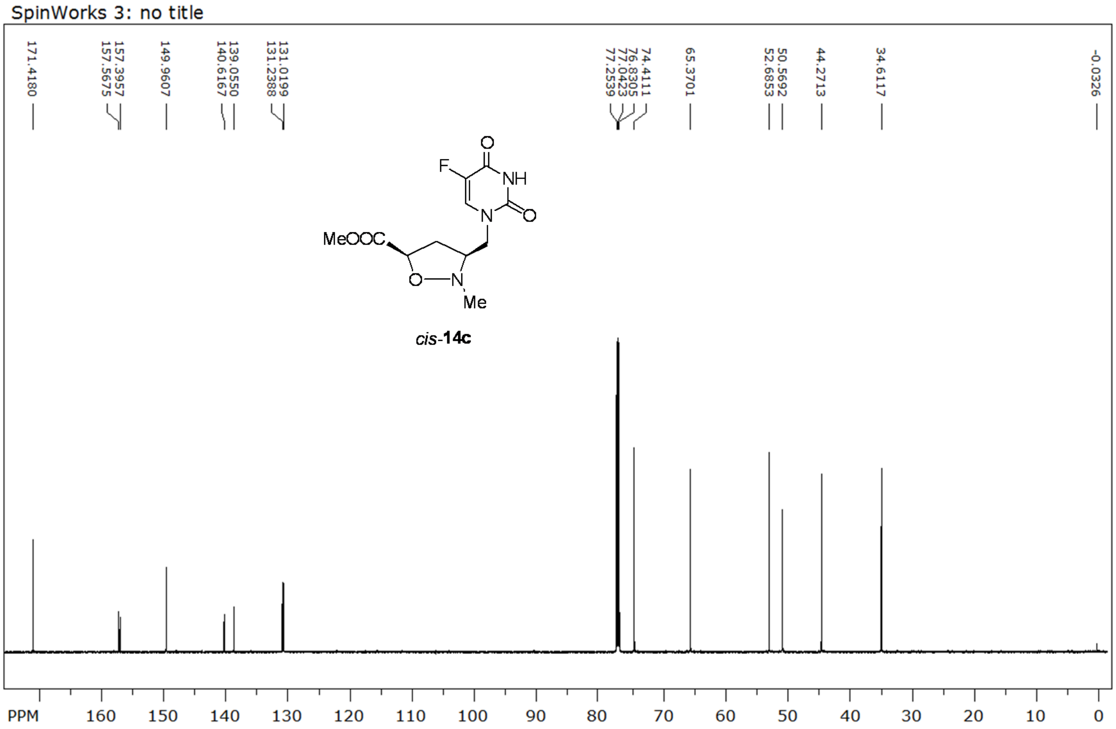

file: E:\IZOKSAZOLIDYNY\jrg-1708\11\fid expt: <zgpg30>
transmitter freq.: 150.950591 MHz
time domain size: 65536 points
width: 36057.69 Hz = 238.8708 ppm = 0.550197 Hz/pt
number of scans: 4000

freq. of 0 ppm: 150.935497 MHz
processed size: 32768 complex points
LB: 0.000 GF: 0.0000
Hz/cm: 1073.991 ppm/cm: 7.11485

SpinWorks 3: no title

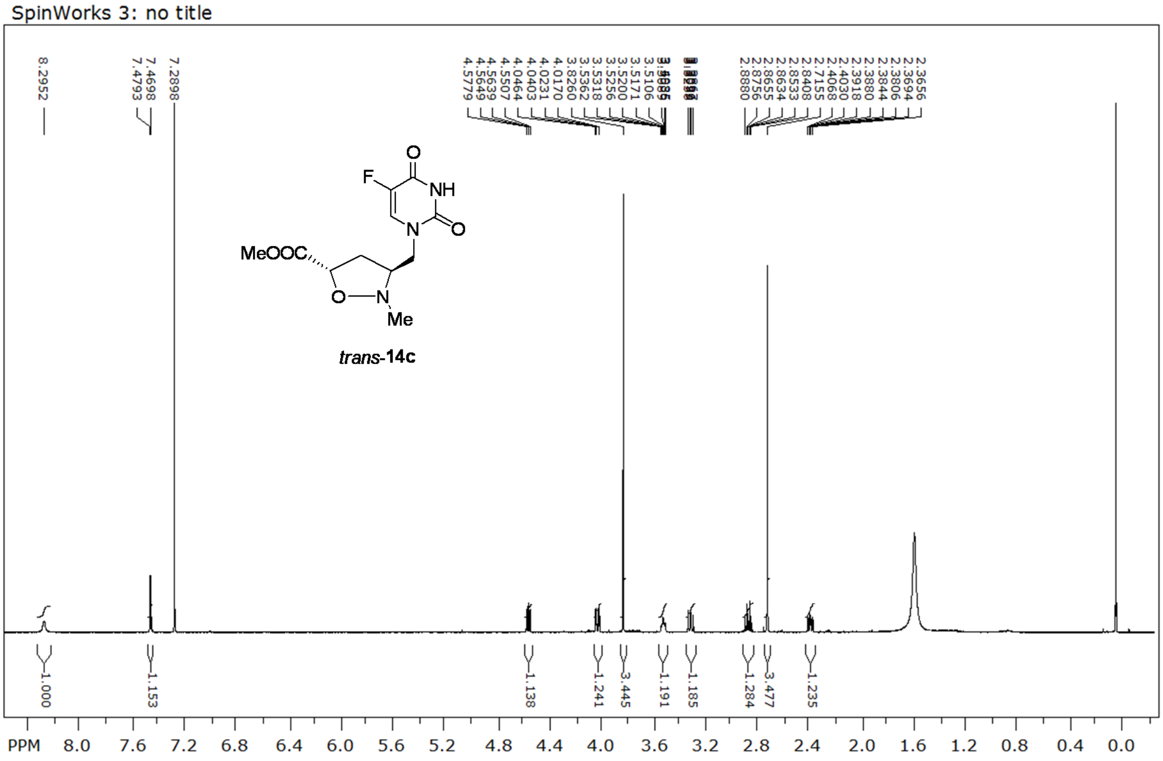

file: E:\IZOKSAZOLIDYNY\jrg-1707\10\fid expt: <zg30>
transmitter freq.: 600.263707 MHz
time domain size: 65536 points
width: 12335.53 Hz = 20.5502 ppm = 0.188225 Hz/pt
number of scans: 16

freq. of 0 ppm: 600.260000 MHz
processed size: 32768 complex points
LB: 0.000 GF: 0.0000
Hz/cm: 212.902 ppm/cm: 0.35468

SpinWorks 3: no title

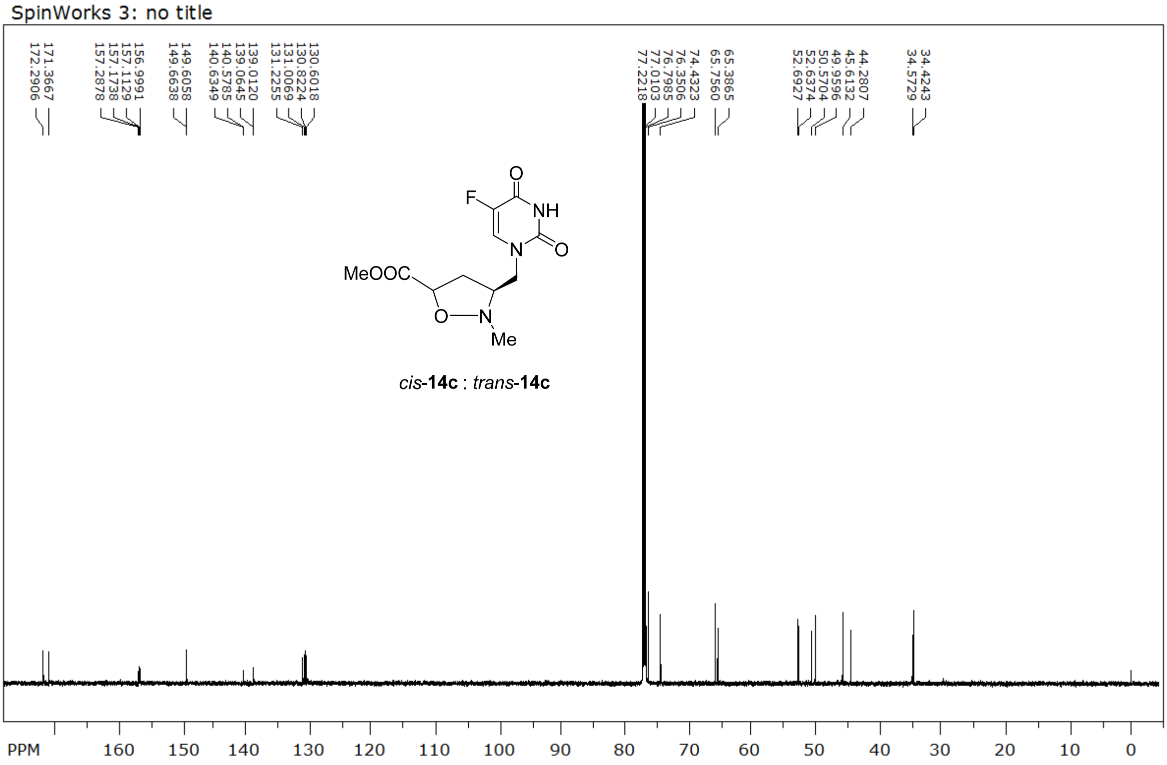

file: E:\IZOKSAZOLIDYNY\jrg-1419\11\fid expt: <zgpg30>
transmitter freq.: 150.950591 MHz
time domain size: 65536 points
width: 36057.69 Hz = 238.8708 ppm = 0.550197 Hz/pt
number of scans: 3000

freq. of 0 ppm: 150.935497 MHz
processed size: 32768 complex points
LB: 0.000 GF: 0.0000
Hz/cm: 1104.949 ppm/cm: 7.31994

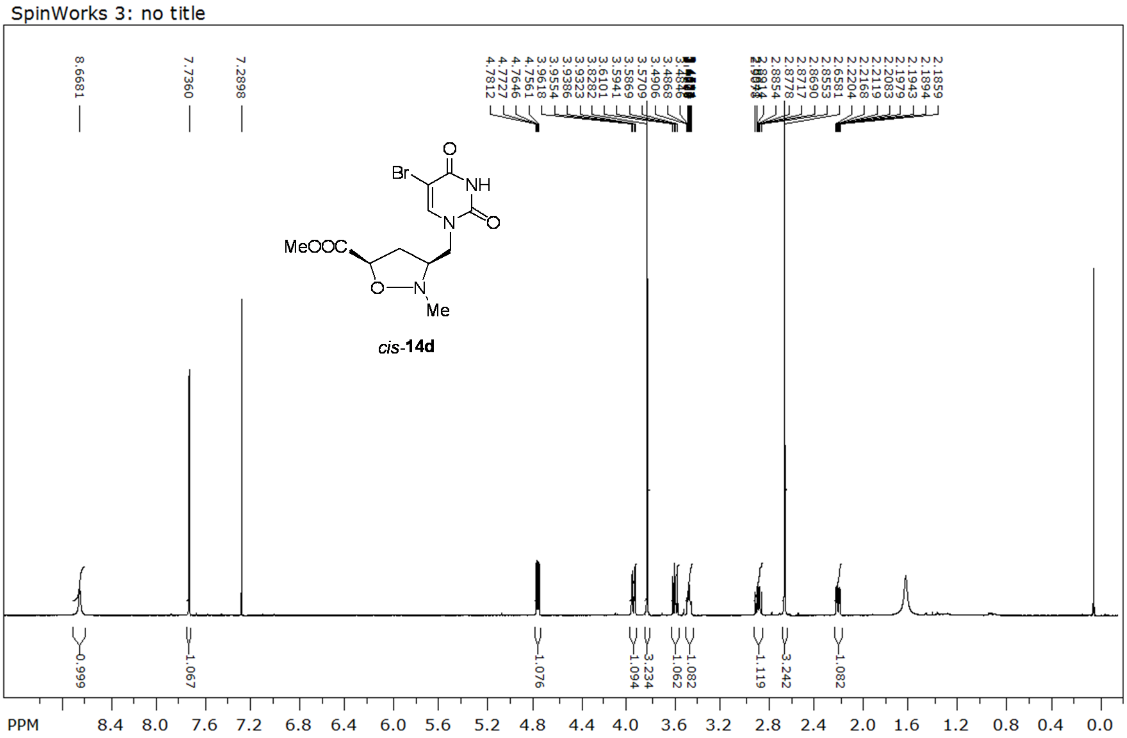

file: E:\IZOKSAZOLIDYNY\jrg-1719\10\fid expt: <zg30>
transmitter freq.: 600.263707 MHz
time domain size: 65536 points
width: 12335.53 Hz = 20.5502 ppm = 0.188225 Hz/pt
number of scans: 16

freq. of 0 ppm: 600.260000 MHz
processed size: 32768 complex points
LB: 0.000 GF: 0.0000
Hz/cm: 228.109 ppm/cm: 0.38001

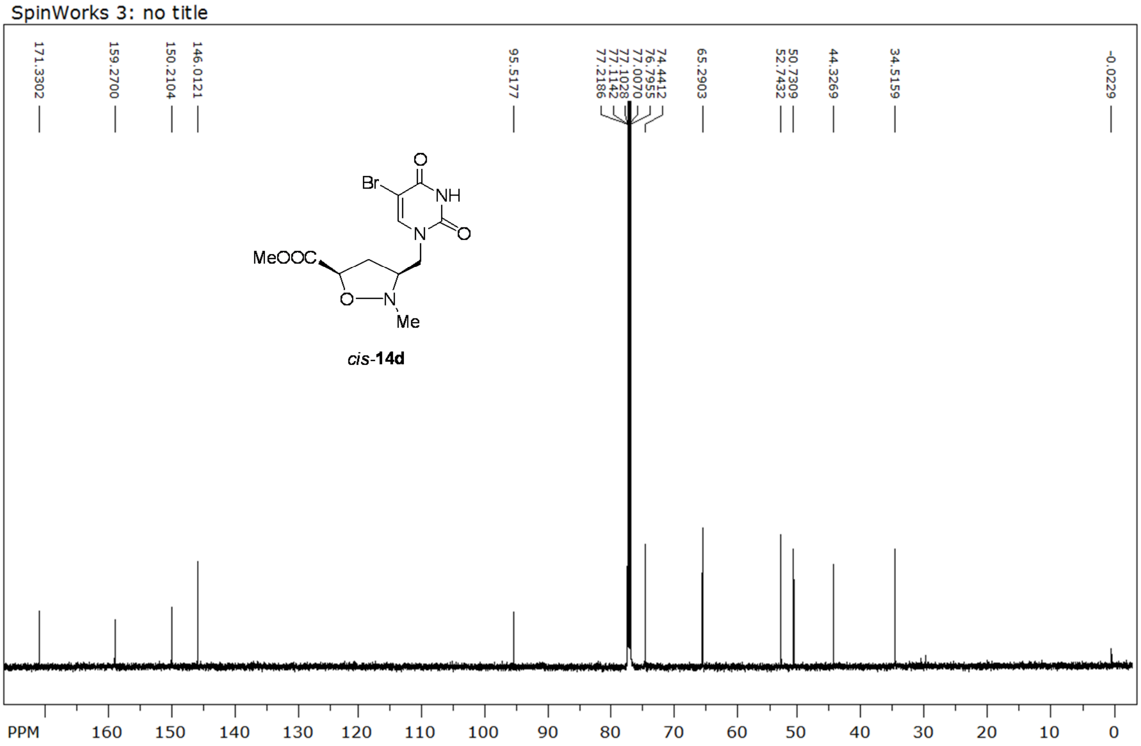

file: E:\IZOKSAZOLIDYNY\jrg-1725\10\fid expt: <zgpg30>
transmitter freq.: 150.950591 MHz
time domain size: 65536 points
width: 36057.69 Hz = 238.8708 ppm = 0.550197 Hz/pt
number of scans: 5000

freq. of 0 ppm: 150.935497 MHz
processed size: 32768 complex points
LB: 0.000 GF: 0.0000
Hz/cm: 1089.073 ppm/cm: 7.21477

SpinWorks 3: no title

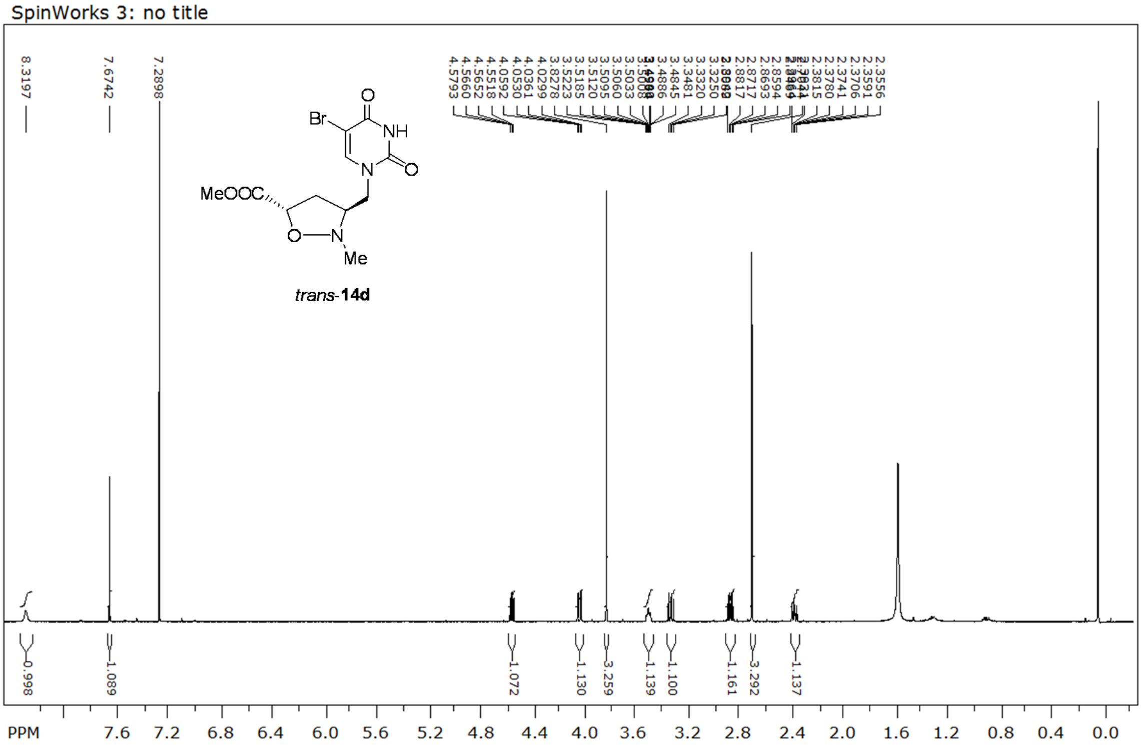

file: E:\IZOKSAZOLIDYNY\jrg-1716\10\fid expt: <zg30>
transmitter freq.: 600.263707 MHz
time domain size: 65536 points
width: 12335.53 Hz = 20.5502 ppm = 0.188225 Hz/pt
number of scans: 16

freq. of 0 ppm: 600.260000 MHz
processed size: 32768 complex points
LB: 0.000 GF: 0.0000
Hz/cm: 209.643 ppm/cm: 0.34925

SpinWorks 3: no title

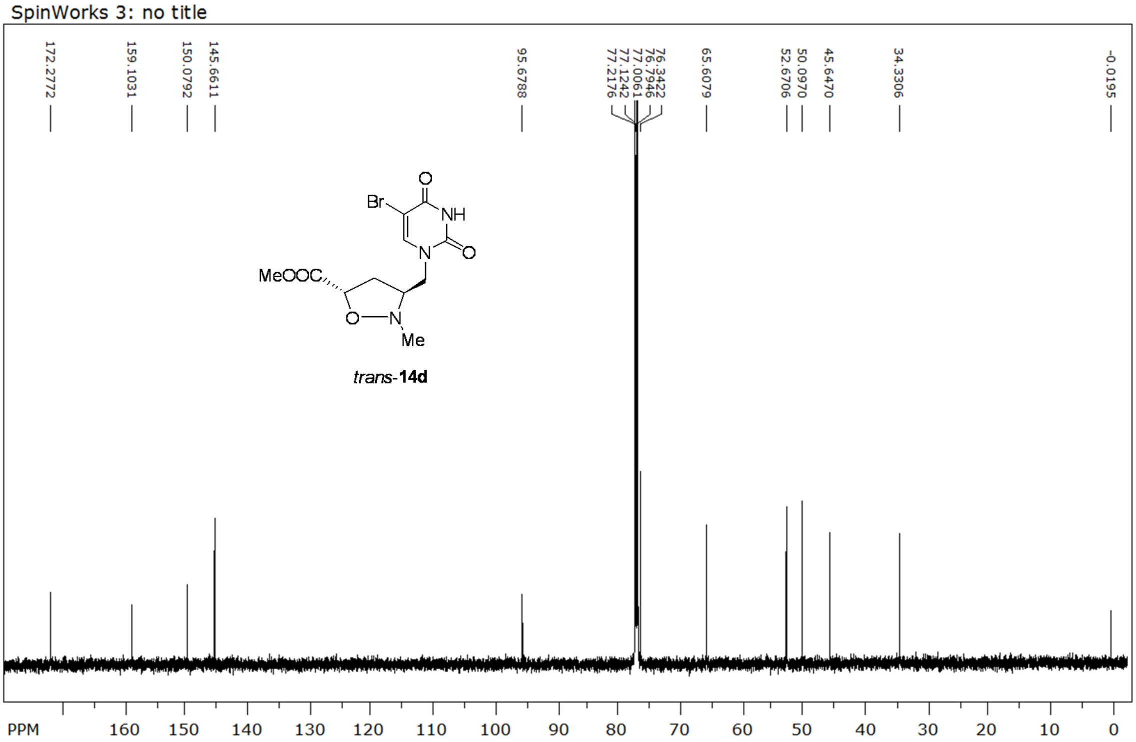

file: E:\IZOKSAZOLIDYNY\jrg-1724\10\fid expt: <zgpg30>
transmitter freq.: 150.950591 MHz
time domain size: 65536 points
width: 36057.69 Hz = 238.8708 ppm = 0.550197 Hz/pt
number of scans: 5000

freq. of 0 ppm: 150.935497 MHz
processed size: 32768 complex points
LB: 0.000 GF: 0.0000
Hz/cm: 1102.568 ppm/cm: 7.30416

SpinWorks 3: no title

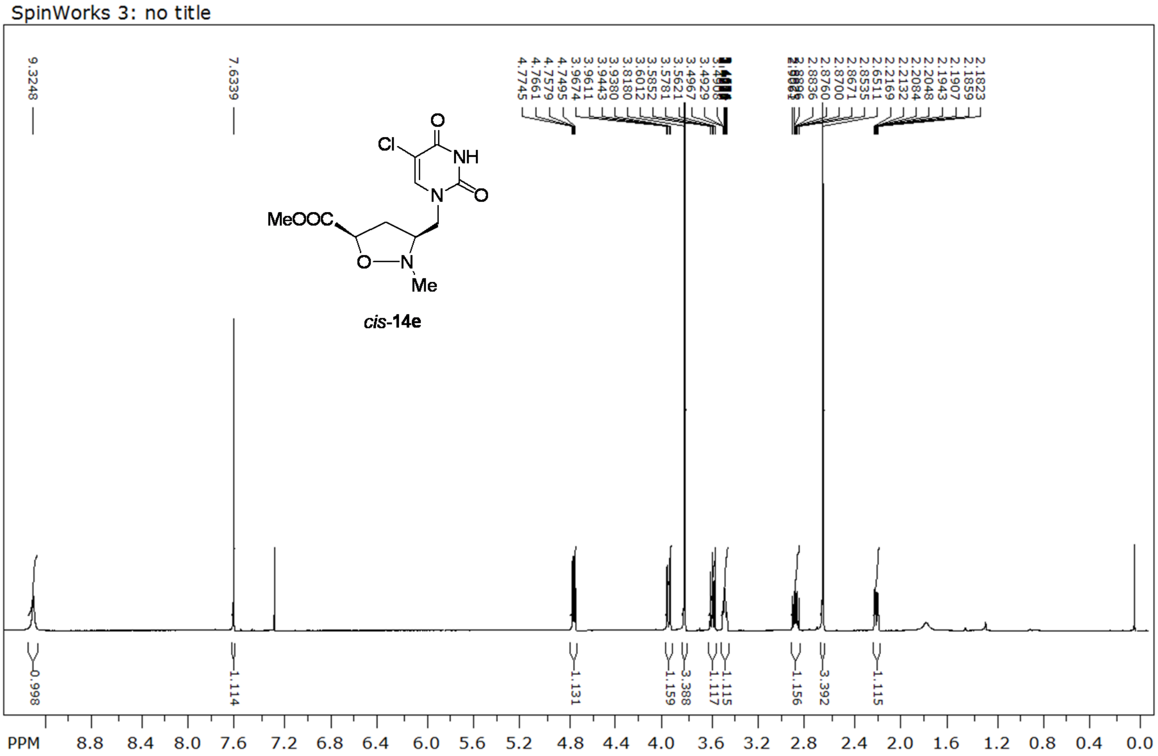

file: E:\IZOKSAZOLIDYNY\jrg-1723\10\fid expt: <zg30>
transmitter freq.: 600.263707 MHz
time domain size: 65536 points
width: 12335.53 Hz = 20.5502 ppm = 0.188225 Hz/pt
number of scans: 16

freq. of 0 ppm: 600.260000 MHz
processed size: 32768 complex points
LB: 0.000 GF: 0.0000
Hz/cm: 232.454 ppm/cm: 0.38725

SpinWorks 3: no title

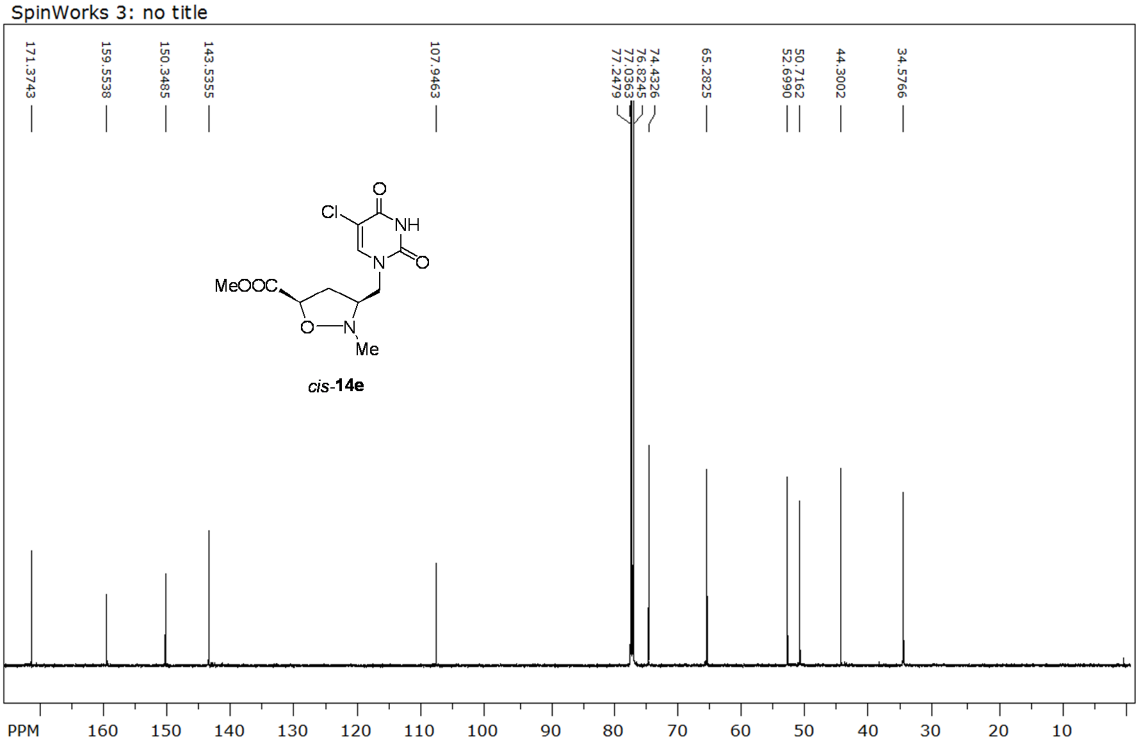

file: E:\IZOKSAZOLIDYNY\jrg-1344\10\fid expt: <zgpg30>
transmitter freq.: 150.950591 MHz
time domain size: 65536 points
width: 36057.69 Hz = 238.8708 ppm = 0.550197 Hz/pt
number of scans: 3072

freq. of 0 ppm: 150.935497 MHz
processed size: 32768 complex points
LB: 0.000 GF: 0.0000
Hz/cm: 1067.641 ppm/cm: 7.07279

SpinWorks 3: no title

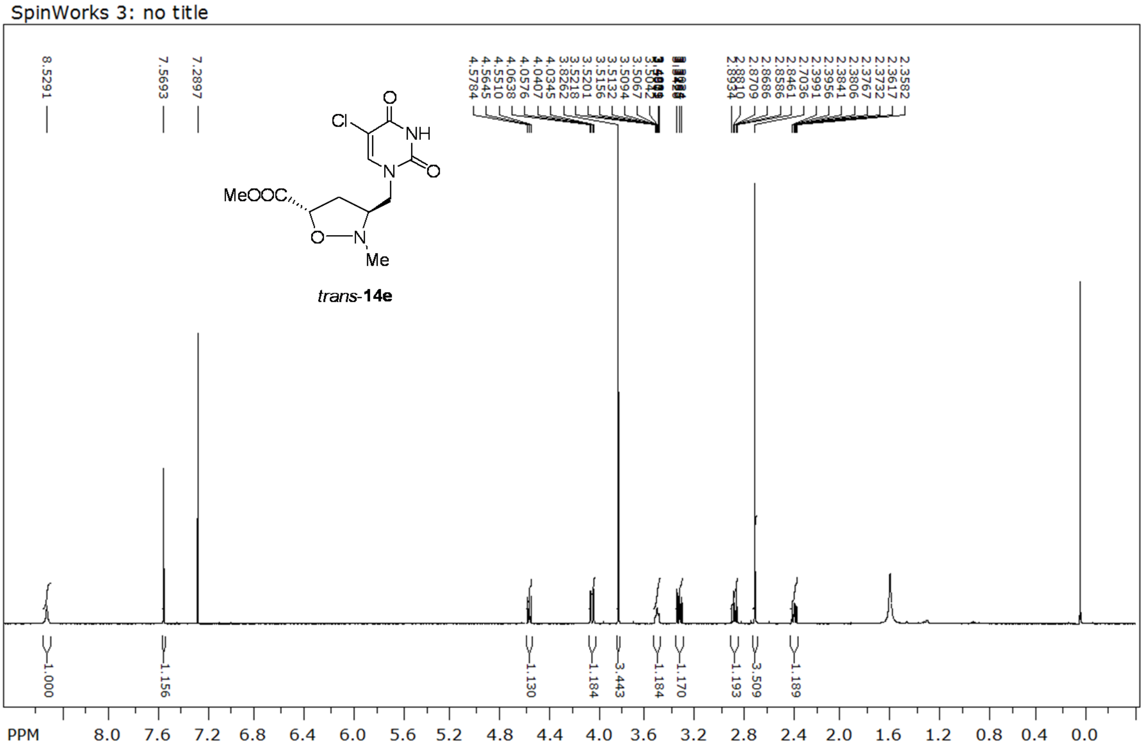

file: E:\IZOKSAZOLIDYNY\jrg-1720\10\fid expt: <zg30>
transmitter freq.: 600.263707 MHz
time domain size: 65536 points
width: 12335.53 Hz = 20.5502 ppm = 0.188225 Hz/pt
number of scans: 16

freq. of 0 ppm: 600.260000 MHz
processed size: 32768 complex points
LB: 0.000 GF: 0.0000
Hz/cm: 223.492 ppm/cm: 0.37232

SpinWorks 3: no title

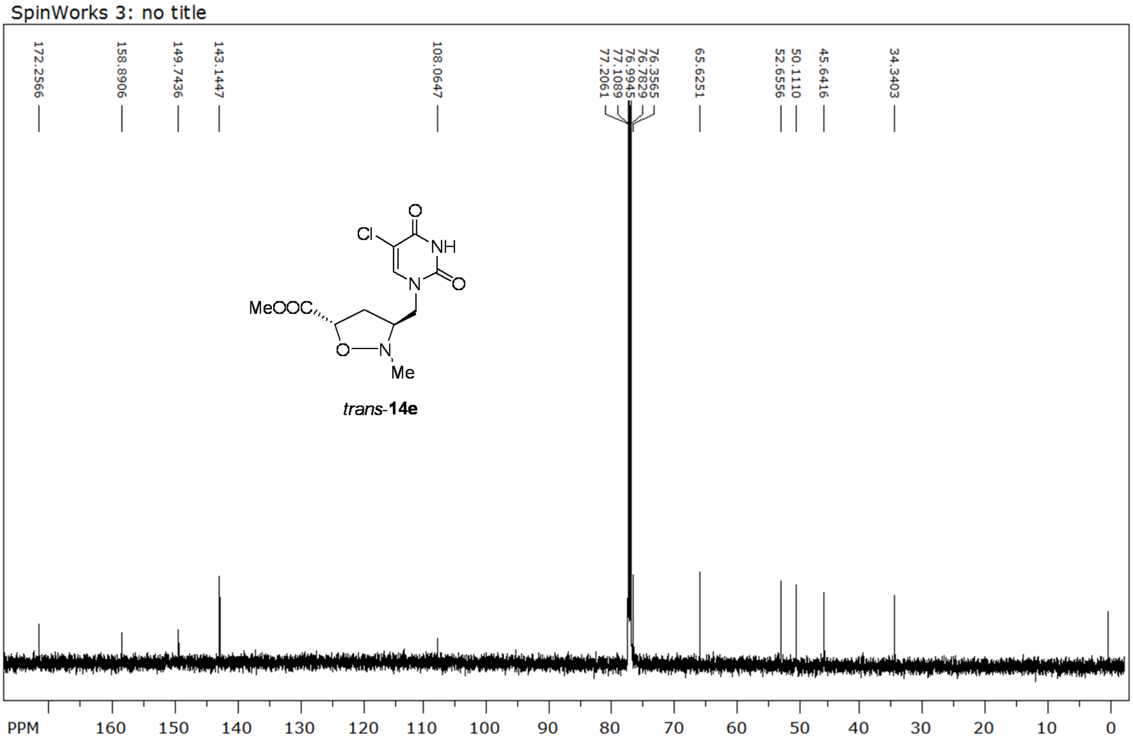

file: E:\IZOKSAZOLIDYNY\jrg-1729\10\fid expt: <zgpg30>
transmitter freq.: 150.950591 MHz
time domain size: 65536 points
width: 36057.69 Hz = 238.8708 ppm = 0.550197 Hz/pt
number of scans: 3000

freq. of 0 ppm: 150.935497 MHz
processed size: 32768 complex points
LB: 0.000 GF: 0.0000
Hz/cm: 1090.661 ppm/cm: 7.22528

SpinWorks 3: no title

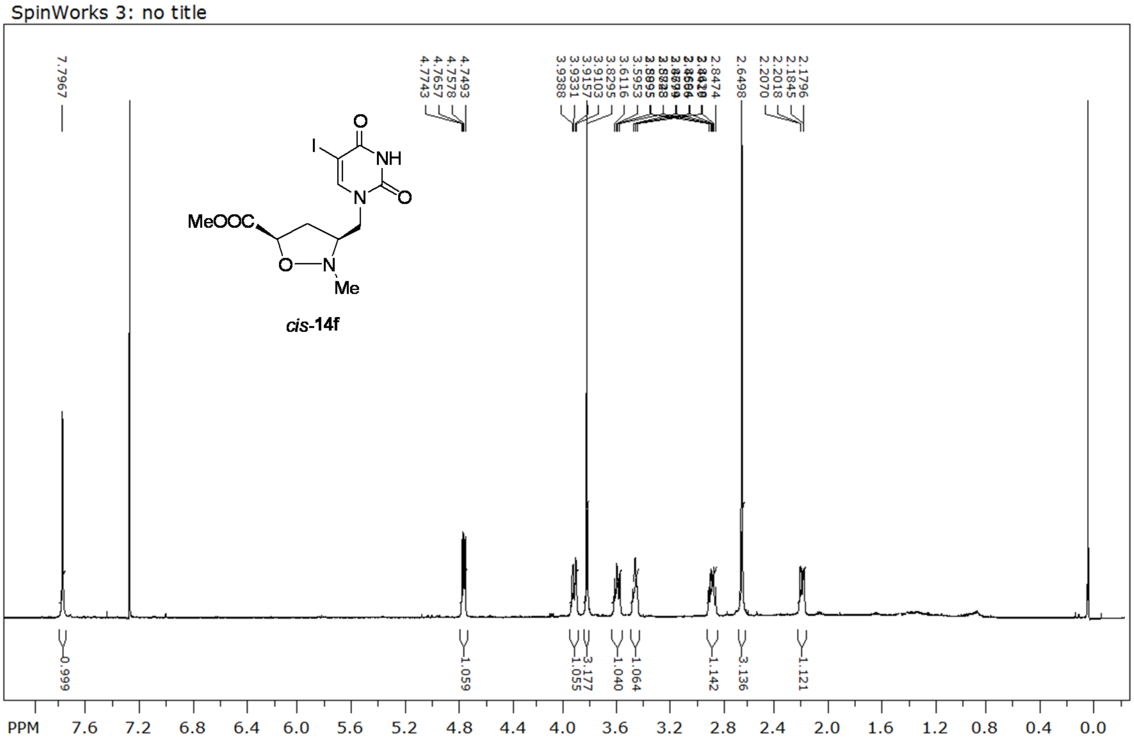

file: E:\IZOKSAZOLIDYNY\jrg-1728\10\fid expt: <zg30>
transmitter freq.: 600.263707 MHz
time domain size: 65536 points
width: 12335.53 Hz = 20.5502 ppm = 0.188225 Hz/pt
number of scans: 16

freq. of 0 ppm: 600.260000 MHz
processed size: 32768 complex points
LB: 0.000 GF: 0.0000
Hz/cm: 203.940 ppm/cm: 0.33975

SpinWorks 3: no title

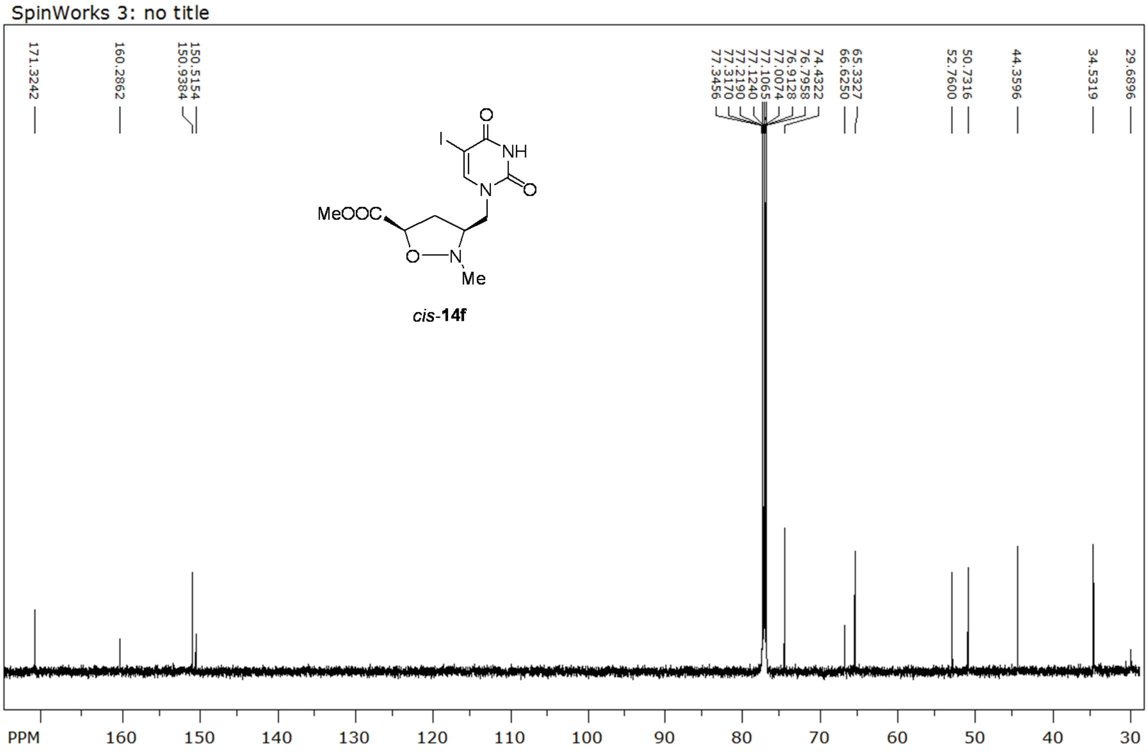

file: E:\IZOKSAZOLIDYNY\jrg-1736\10\fid expt: <zgpg30>
transmitter freq.: 150.950591 MHz
time domain size: 65536 points
width: 36057.69 Hz = 238.8708 ppm = 0.550197 Hz/pt
number of scans: 5000

freq. of 0 ppm: 150.935497 MHz
processed size: 32768 complex points
LB: 0.000 GF: 0.0000
Hz/cm: 885.864 ppm/cm: 5.86857

SpinWorks 3: no title

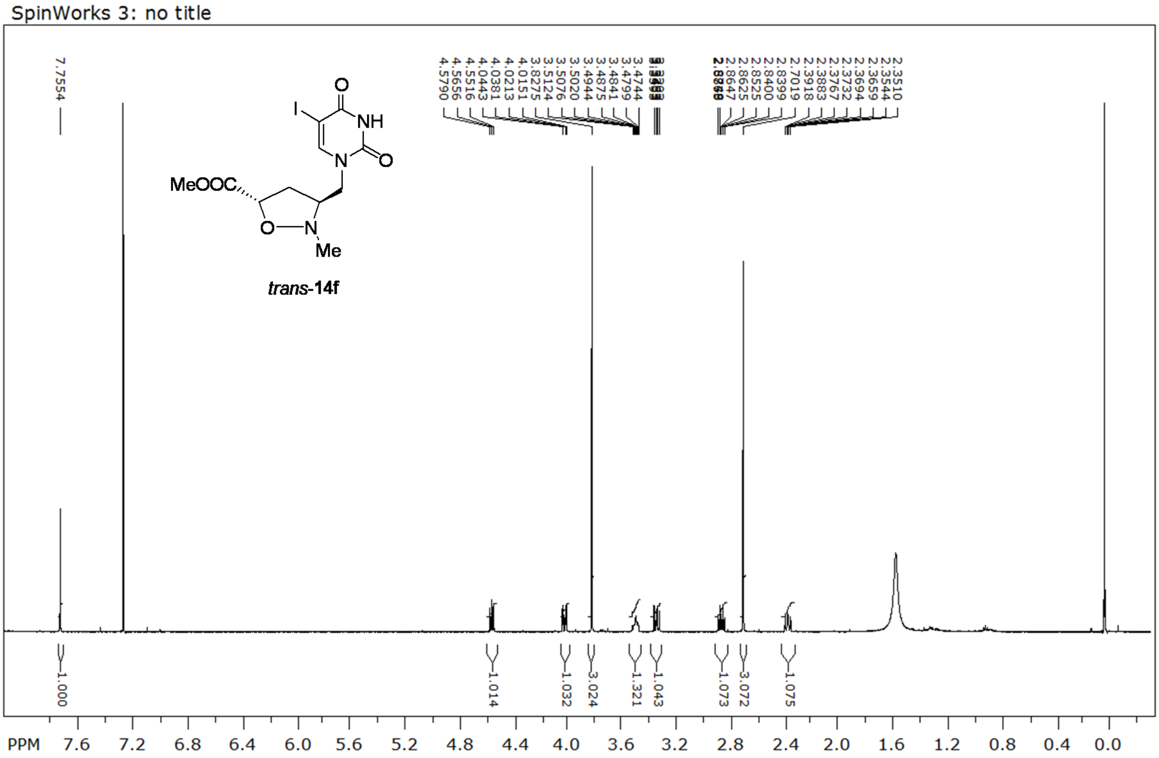

file: E:\IZOKSAZOLIDYNY\jrg-1726\10\fid expt: <zg30>
transmitter freq.: 600.263707 MHz
time domain size: 65536 points
width: 12335.53 Hz = 20.5502 ppm = 0.188225 Hz/pt
number of scans: 16

freq. of 0 ppm: 600.260000 MHz
processed size: 32768 complex points
LB: 0.000 GF: 0.0000
Hz/cm: 203.669 ppm/cm: 0.33930

SpinWorks 3: no title

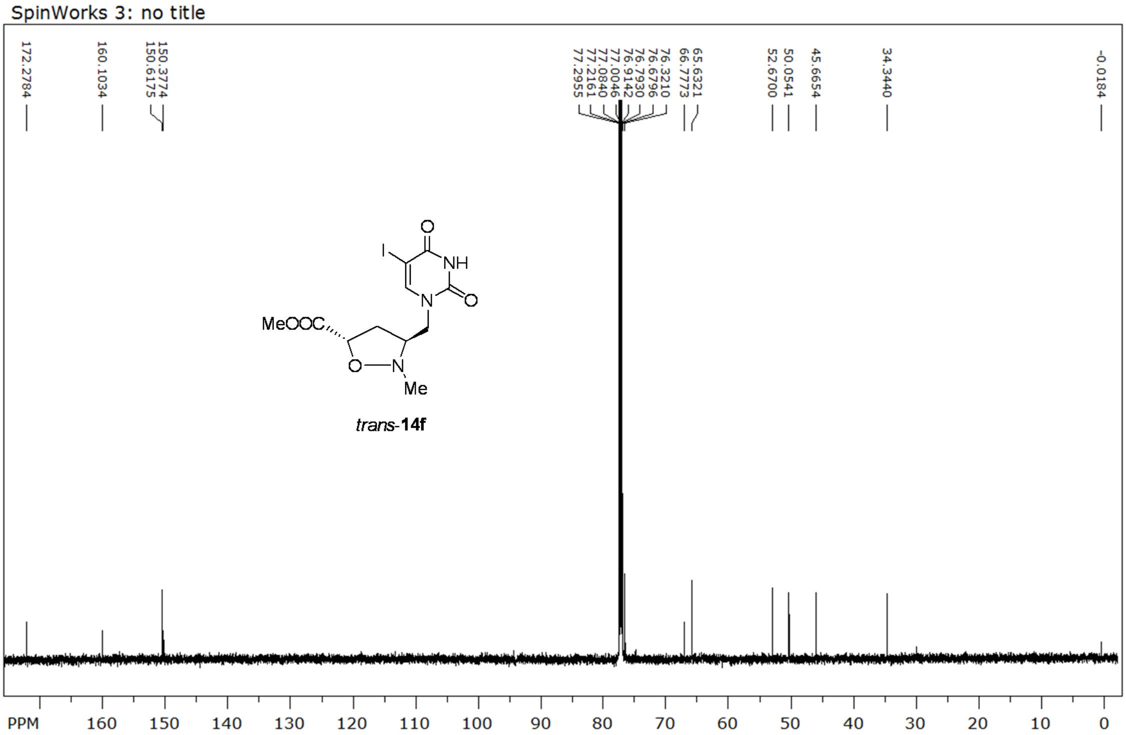

file: E:\IZOKSAZOLIDYNY\jrg-1735\10\fid expt: <zgpg30>
transmitter freq.: 150.950591 MHz
time domain size: 65536 points
width: 36057.69 Hz = 238.8708 ppm = 0.550197 Hz/pt
number of scans: 4000

freq. of 0 ppm: 150.935497 MHz
processed size: 32768 complex points
LB: 0.000 GF: 0.0000
Hz/cm: 1077.960 ppm/cm: 7.14115

SpinWorks 3: no title

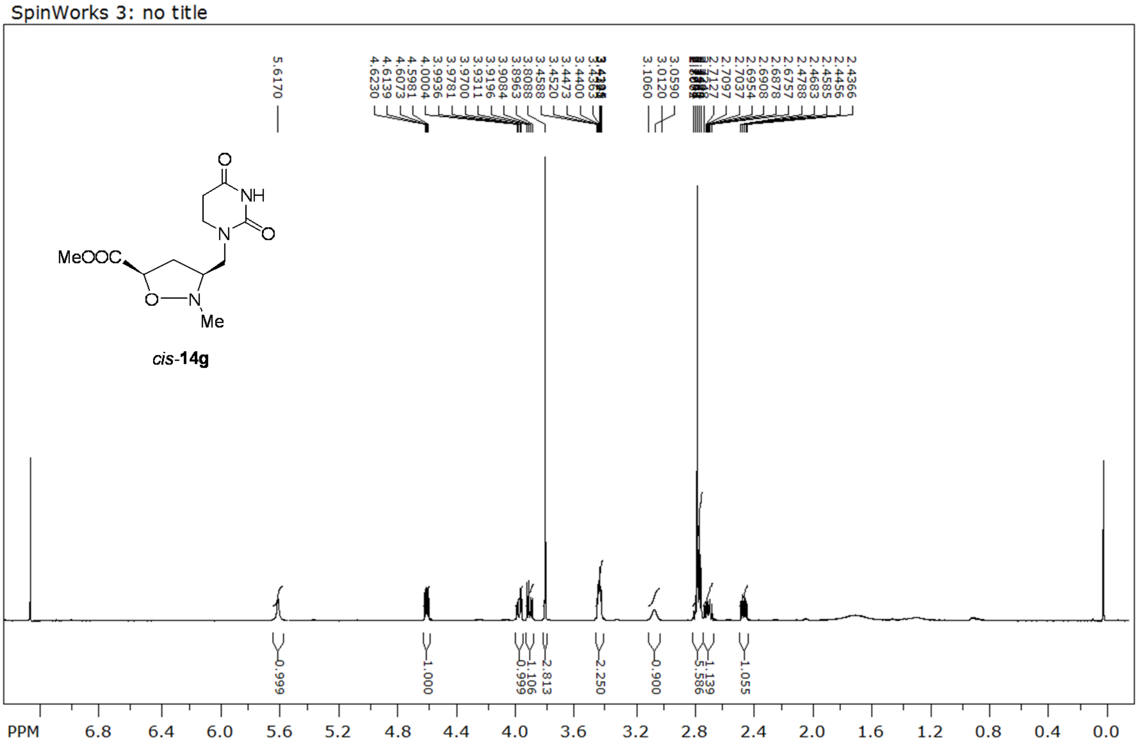

file: E:\IZOKSAZOLIDYNY\jrg-1758\10\fid expt: <zg30>
transmitter freq.: 600.263707 MHz
time domain size: 65536 points
width: 12335.53 Hz = 20.5502 ppm = 0.188225 Hz/pt
number of scans: 16

freq. of 0 ppm: 600.260000 MHz
processed size: 32768 complex points
LB: 0.000 GF: 0.0000
Hz/cm: 183.030 ppm/cm: 0.30492

SpinWorks 3: no title

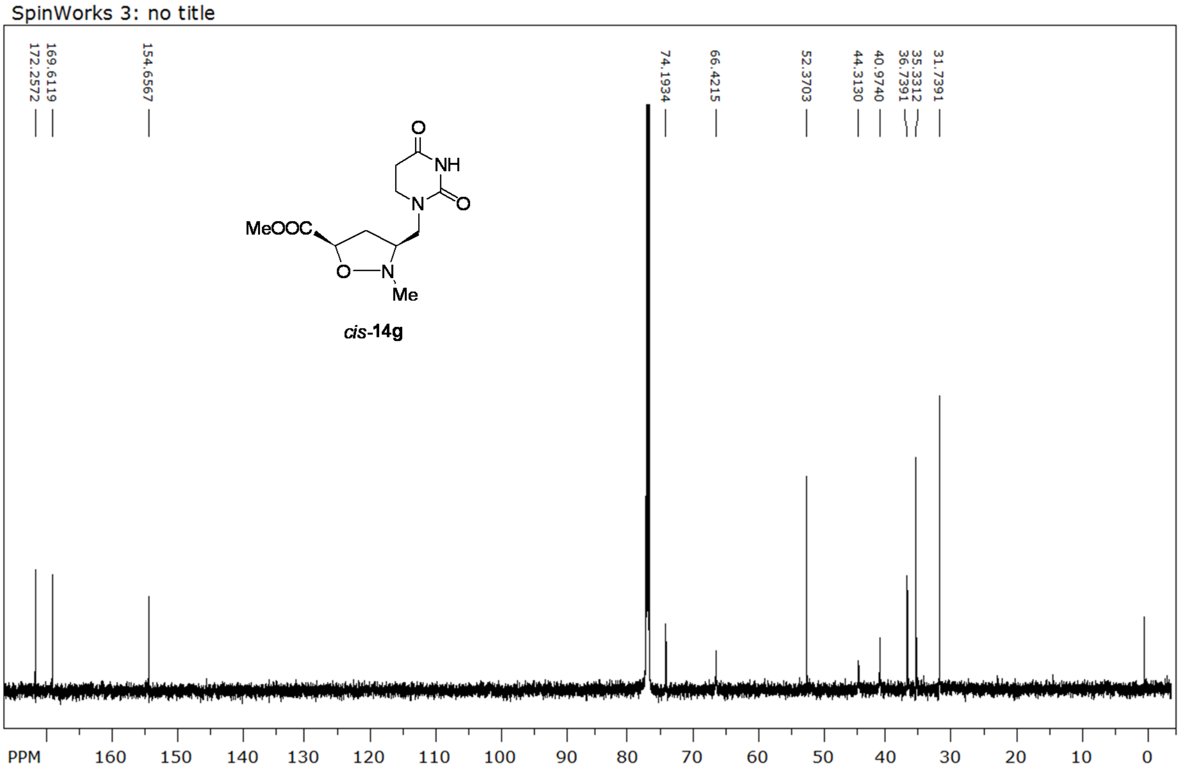

file: E:\IZOKSAZOLIDYNY\jrg-1758\11\fid expt: <zgpg30>
transmitter freq.: 150.950591 MHz
time domain size: 65536 points
width: 36057.69 Hz = 238.8708 ppm = 0.550197 Hz/pt
number of scans: 3072

freq. of 0 ppm: 150.935497 MHz
processed size: 32768 complex points
LB: 0.000 GF: 0.0000
Hz/cm: 1095.424 ppm/cm: 7.25684

SpinWorks 3: no title

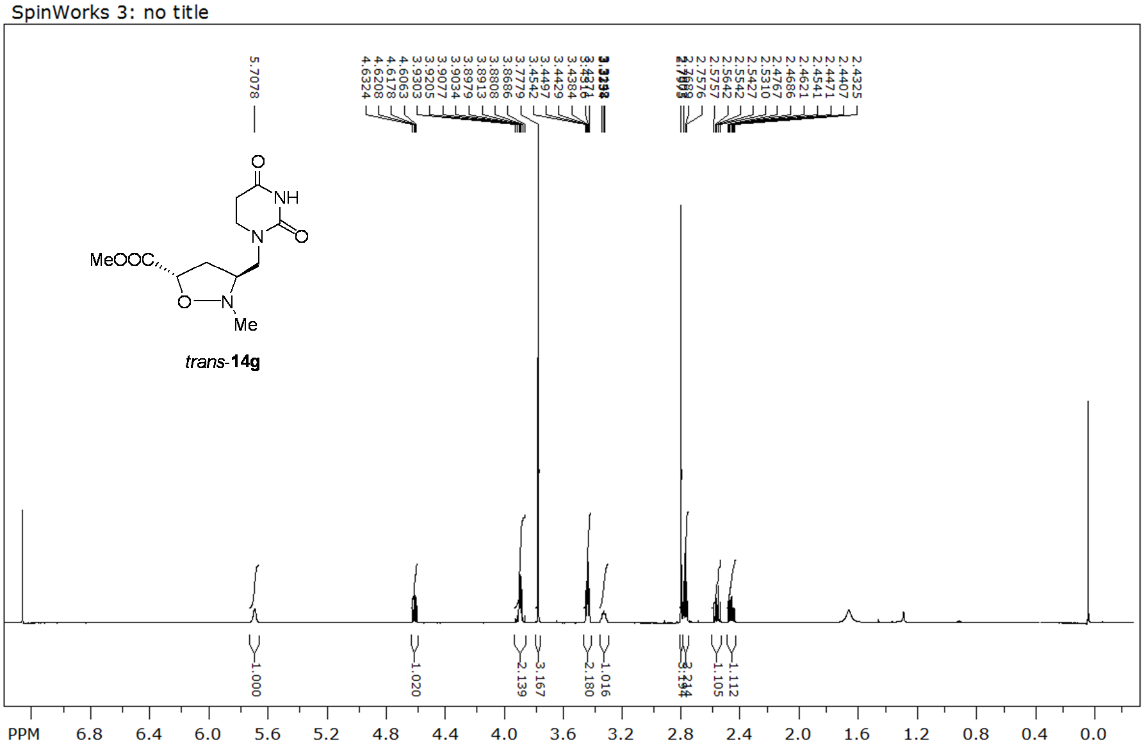

file: E:\IZOKSAZOLIDYNY\jrg-1715\10\fid expt: <zg30>
transmitter freq.: 600.263707 MHz
time domain size: 65536 points
width: 12335.53 Hz = 20.5502 ppm = 0.188225 Hz/pt
number of scans: 16

freq. of 0 ppm: 600.260000 MHz
processed size: 32768 complex points
LB: 0.000 GF: 0.0000
Hz/cm: 185.203 ppm/cm: 0.30854

SpinWorks 3: no title

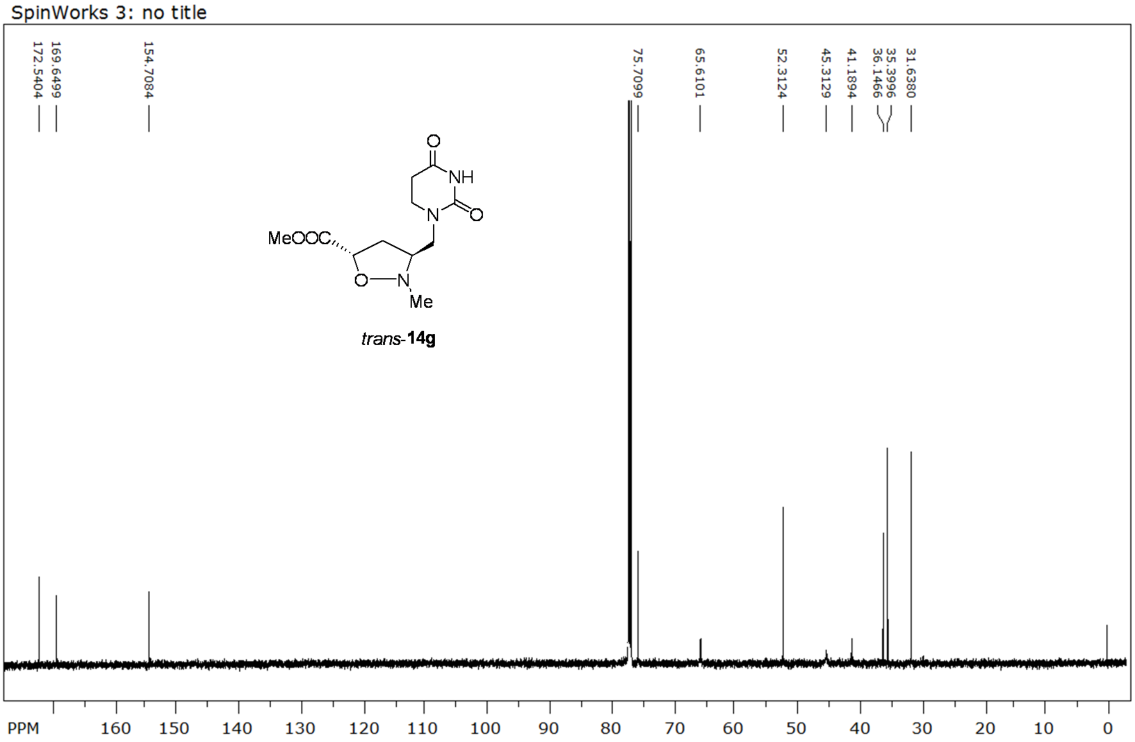

file: E:\IZOKSAZOLIDYNY\jrg-1715\11\fid expt: <zgpg30>
transmitter freq.: 150.950591 MHz
time domain size: 65536 points
width: 36057.69 Hz = 238.8708 ppm = 0.550197 Hz/pt
number of scans: 4000

freq. of 0 ppm: 150.935497 MHz
processed size: 32768 complex points
LB: 0.000 GF: 0.0000
Hz/cm: 1094.630 ppm/cm: 7.25158

SpinWorks 3: no title

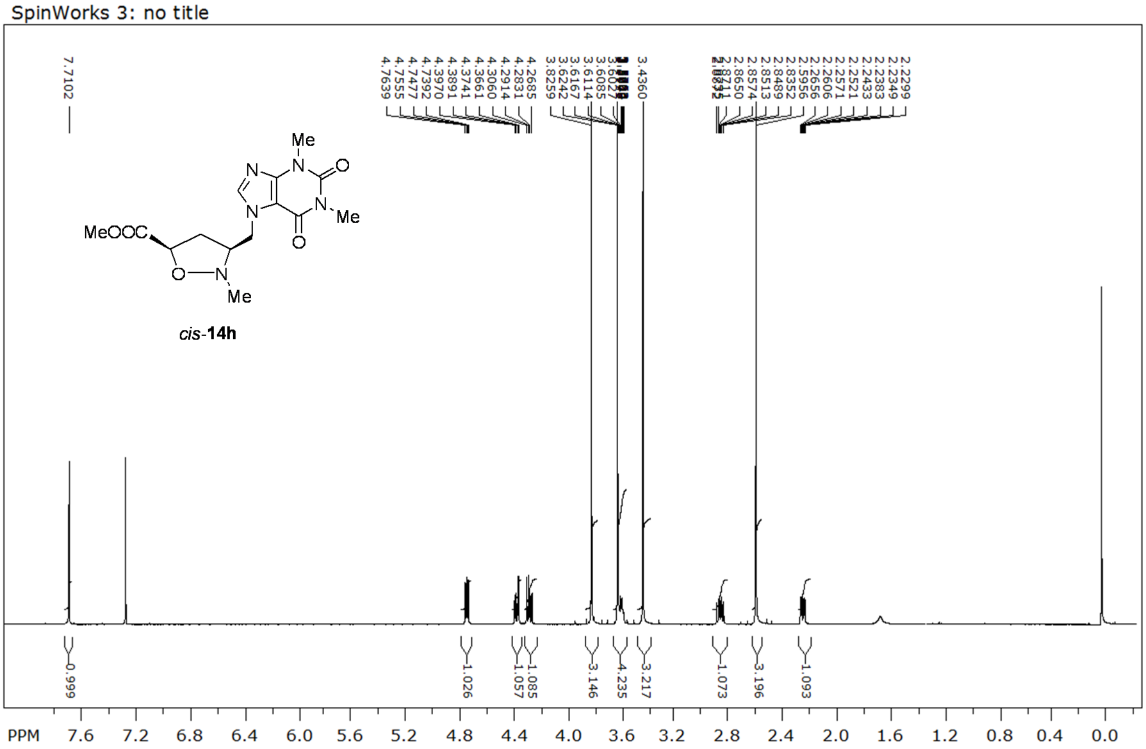

file: E:\IZOKSAZOLIDYNY\jrg-1475\10\fid expt: <zg30>
transmitter freq.: 600.263707 MHz
time domain size: 65536 points
width: 12335.53 Hz = 20.5502 ppm = 0.188225 Hz/pt
number of scans: 16

freq. of 0 ppm: 600.260000 MHz
processed size: 32768 complex points
LB: 0.000 GF: 0.0000
Hz/cm: 202.582 ppm/cm: 0.33749

SpinWorks 3: no title

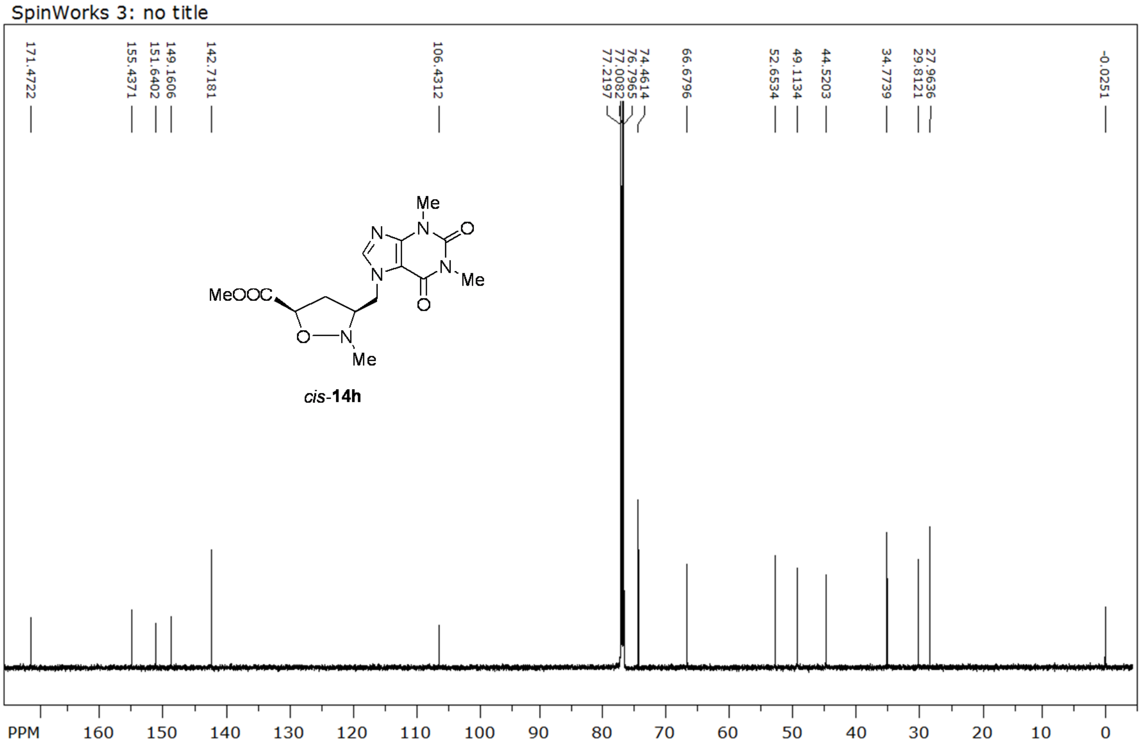

file: E:\IZOKSAZOLIDYNY\jrg-1475\11\fid expt: <zgpg30>
transmitter freq.: 150.950591 MHz
time domain size: 65536 points
width: 36057.69 Hz = 238.8708 ppm = 0.550197 Hz/pt

freq. of 0 ppm: 150.935497 MHz
processed size: 32768 complex points
LB: 0.000 GF: 0.0000
Hz/cm: 1088.279 ppm/cm: 7.20951

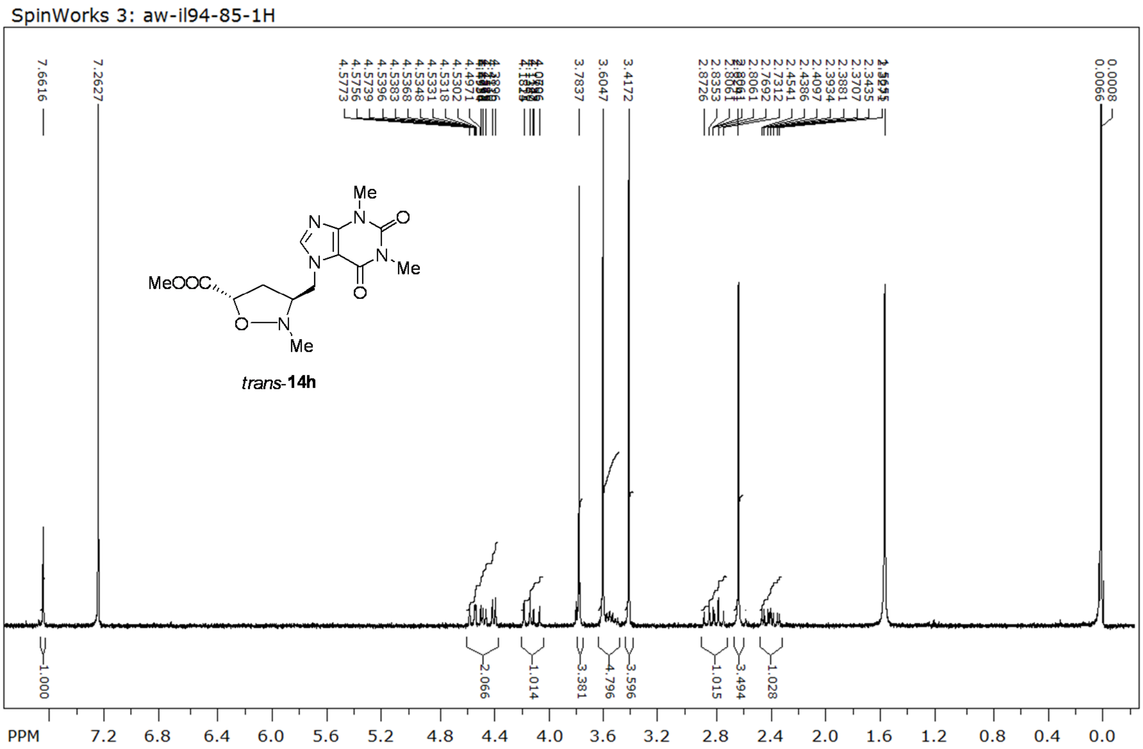

file: ...IZOKSAZOLIDYNY\jrg-1512-1H.fid\fid block# 1 expt: "s2pul"
transmitter freq.: 199.929728 MHz
time domain size: 32000 points
width: 4500.00 Hz = 22.5079 ppm = 0.140625 Hz/pt
number of scans: 16

freq. of 0 ppm: 199.928727 MHz
processed size: 65536 complex points
LB: 0.000 GF: 0.0000
Hz/cm: 65.482 ppm/cm: 0.32752

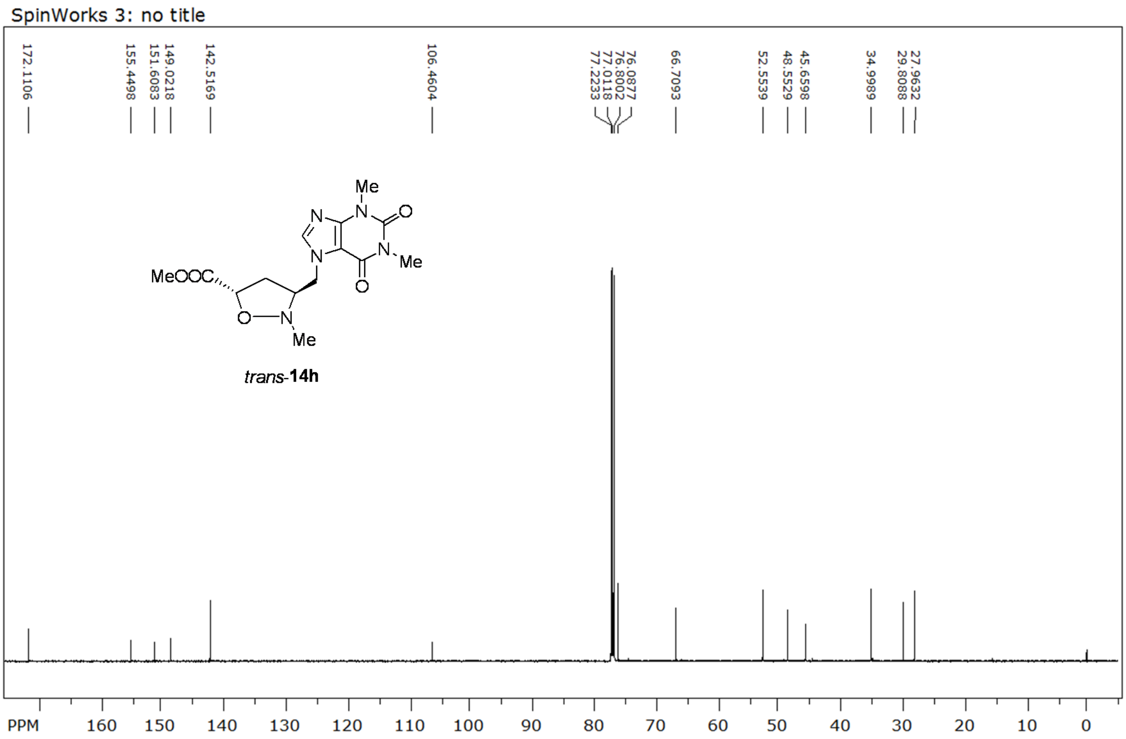

file: E:\IZOKSAZOLIDYNY\jrg-1514\10\fid expt: <zgpg30>
transmitter freq.: 150.950591 MHz
time domain size: 65536 points
width: 36057.69 Hz = 238.8708 ppm = 0.550197 Hz/pt
number of scans: 5000

freq. of 0 ppm: 150.935497 MHz
processed size: 32768 complex points
LB: 0.000 GF: 0.0000
Hz/cm: 1093.836 ppm/cm: 7.24632

SpinWorks 3: jrg0693h

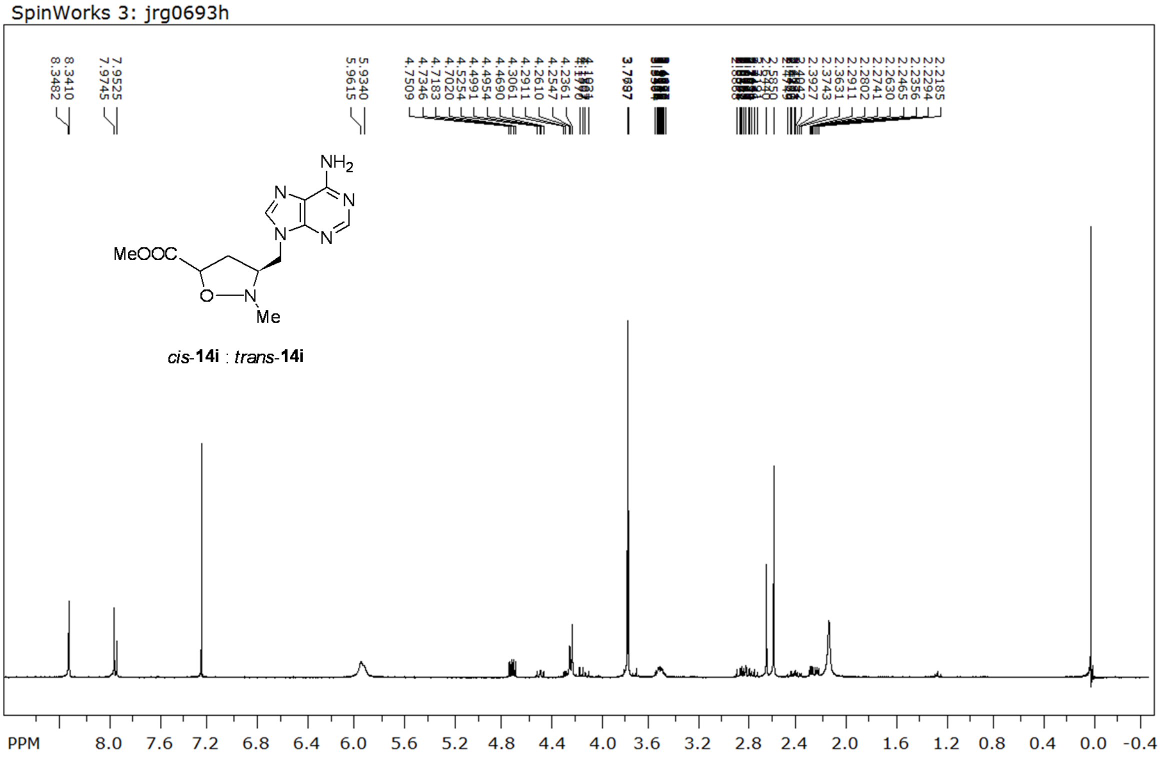

file: E:\IZOKSAZOLIDYNY\jrg0693h.fid\fid block# 1 expt: "s2pul"
transmitter freq.: 300.063051 MHz
time domain size: 52032 points
width: 6500.00 Hz = 21.6621 ppm = 0.124923 Hz/pt
number of scans: 16

freq. of 0 ppm: 300.060200 MHz
processed size: 65536 complex points
LB: 0.000 GF: 0.0000
Hz/cm: 112.328 ppm/cm: 0.37435

SpinWorks 3: no title

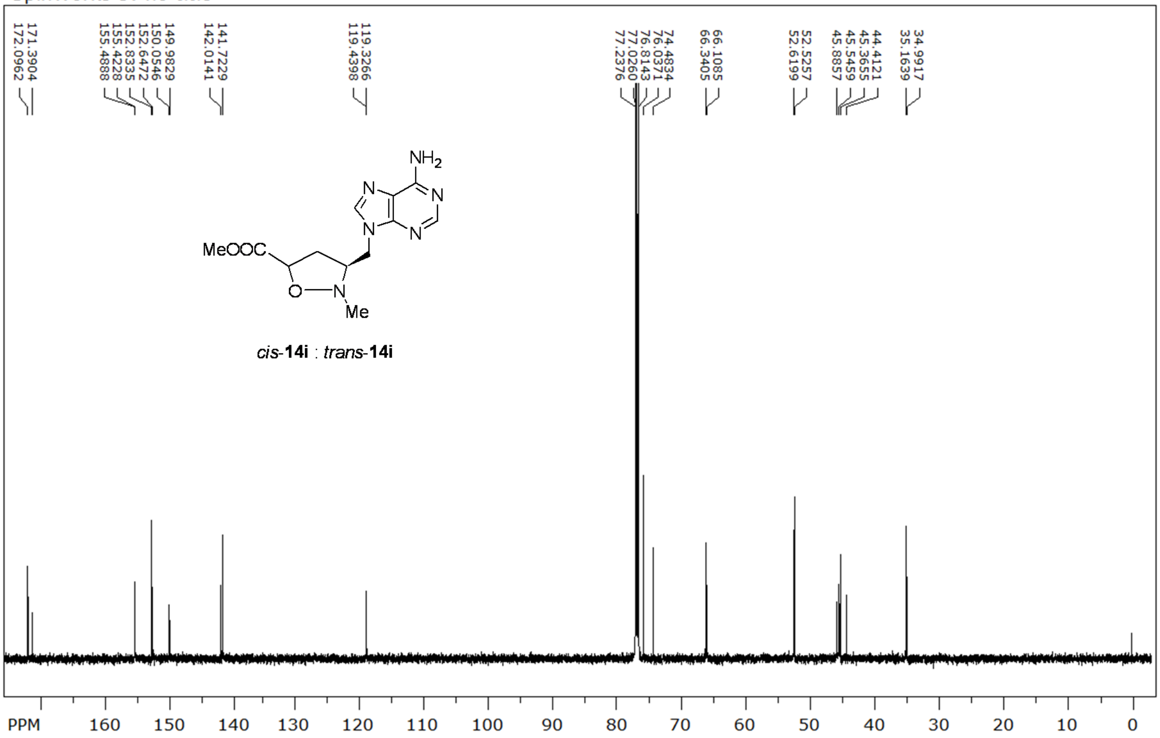

file: E:\IZOKSAZOLIDYNY\jrg-0871\10\fid expt: <zgpg30>
transmitter freq.: 150.950591 MHz
time domain size: 65536 points
width: 36057.69 Hz = 238.8708 ppm = 0.550197 Hz/pt
number of scans: 1024

freq. of 0 ppm: 150.935497 MHz
processed size: 32768 complex points
LB: 0.000 GF: 0.0000
Hz/cm: 1080.342 ppm/cm: 7.15692

SpinWorks 3: no title

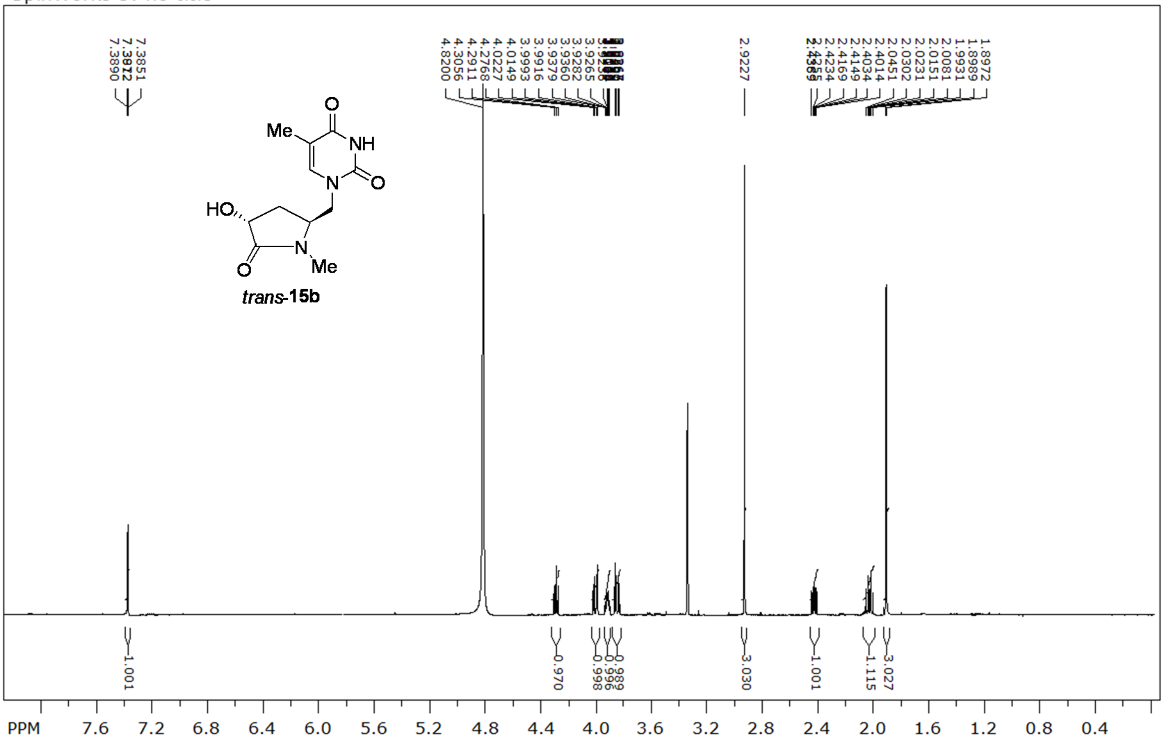

file: E:\gamma jrg\jrg-1714\10\fid expt: <zg30>
transmitter freq.: 600.263707 MHz
time domain size: 65536 points
width: 12335.53 Hz = 20.5502 ppm = 0.188225 Hz/pt
number of scans: 16

freq. of 0 ppm: 600.260000 MHz
processed size: 32768 complex points
LB: 0.000 GF: 0.0000
Hz/cm: 200.138 ppm/cm: 0.33342

SpinWorks 3: no title

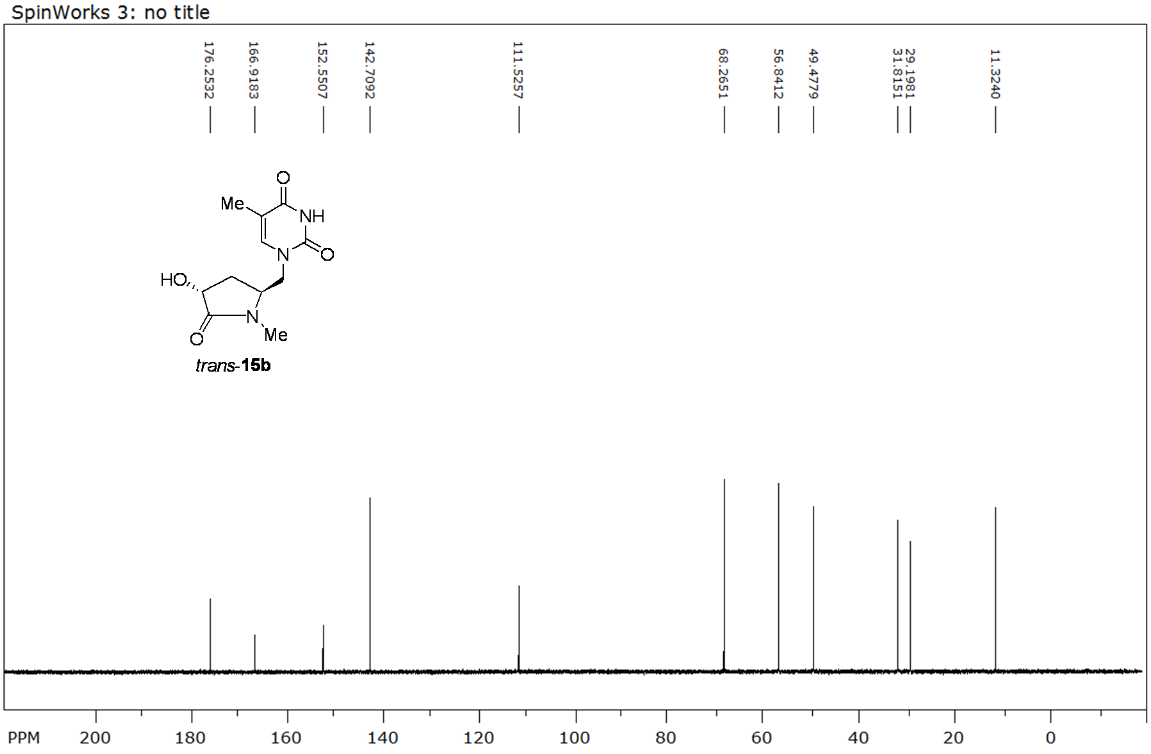

file: E:\gamma jrg\jrg-1759\12\fid expt: <zgpg30>
transmitter freq.: 150.950591 MHz
time domain size: 65536 points
width: 36057.69 Hz = 238.8708 ppm = 0.550197 Hz/pt
number of scans: 3072

freq. of 0 ppm: 150.935497 MHz
processed size: 32768 complex points
LB: 0.000 GF: 0.0000
Hz/cm: 1442.308 ppm/cm: 9.55483

SpinWorks 3:

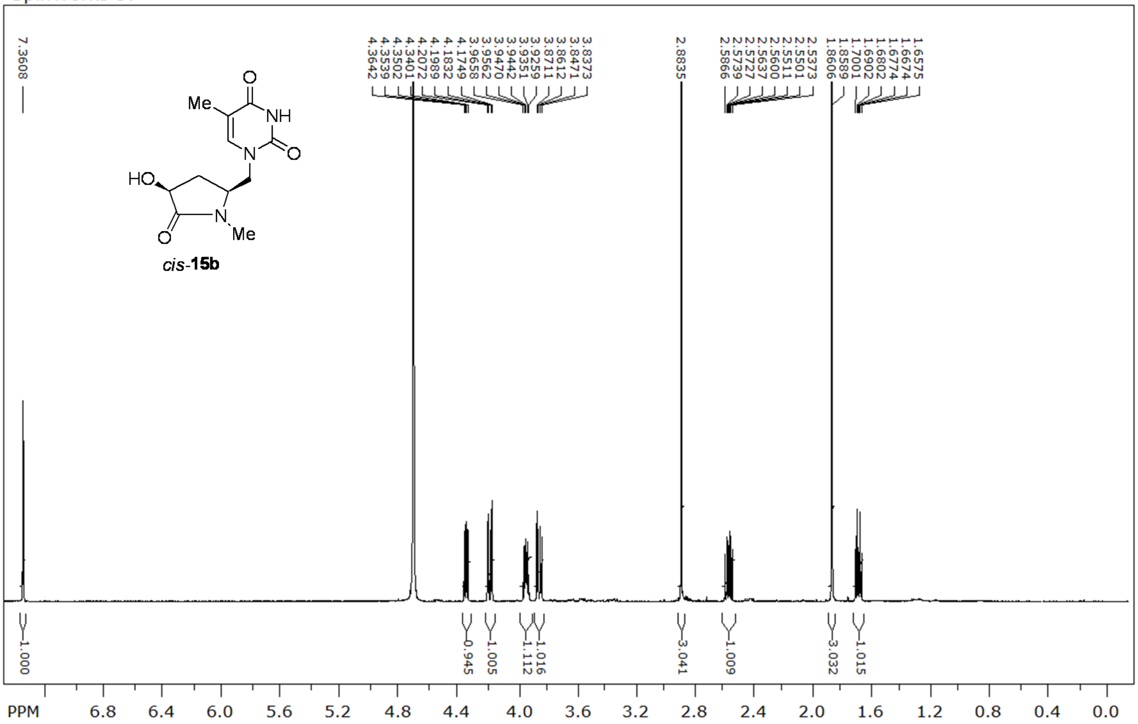

file: E:\gamma jrg\jrg-1751\1\fid expt: <zg30>
transmitter freq.: 600.263707 MHz
time domain size: 65536 points
width: 12335.53 Hz = 20.5502 ppm = 0.188225 Hz/pt
number of scans: 16

freq. of 0 ppm: 600.260000 MHz
processed size: 32768 complex points
LB: 0.000 GF: 0.0000
Hz/cm: 183.845 ppm/cm: 0.30627

SpinWorks 3: no title

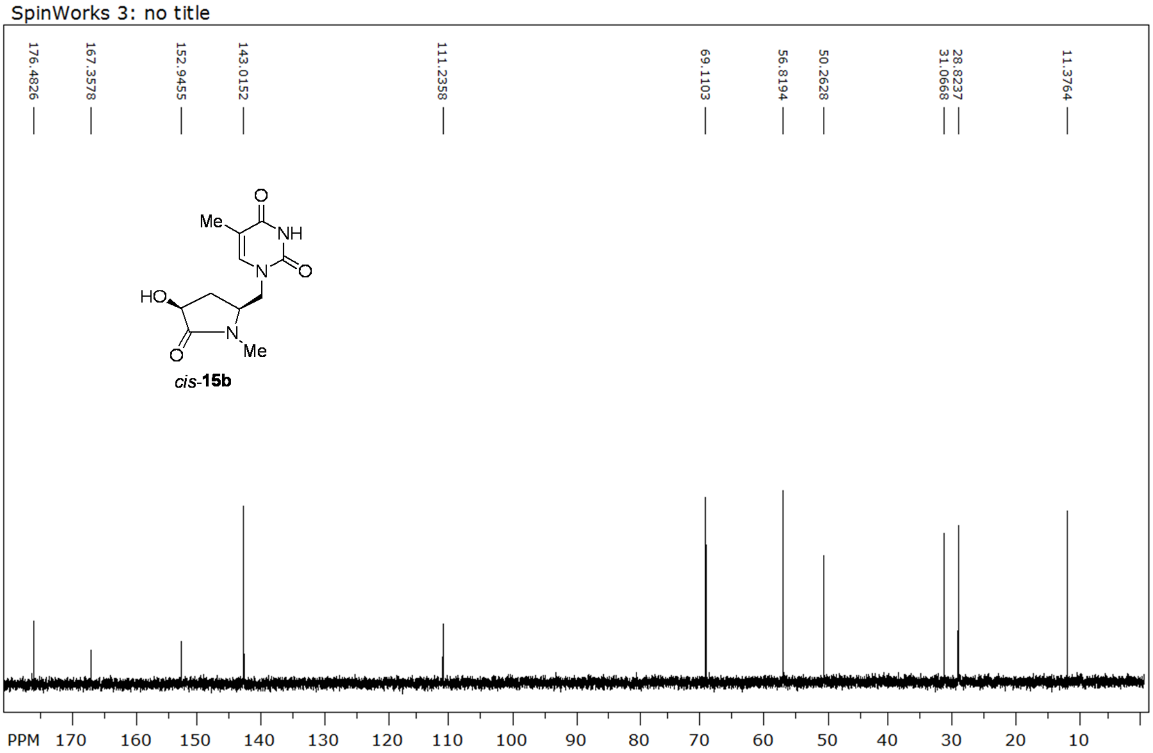

file: E:\gamma jrg\jrg-1760\12\fid expt: <zgpg30>
transmitter freq.: 150.950591 MHz
time domain size: 65536 points
width: 36057.69 Hz = 238.8708 ppm = 0.550197 Hz/pt
number of scans: 5000

freq. of 0 ppm: 150.935497 MHz
processed size: 32768 complex points
LB: 0.000 GF: 0.0000
Hz/cm: 1100.186 ppm/cm: 7.28839

SpinWorks 3: no title

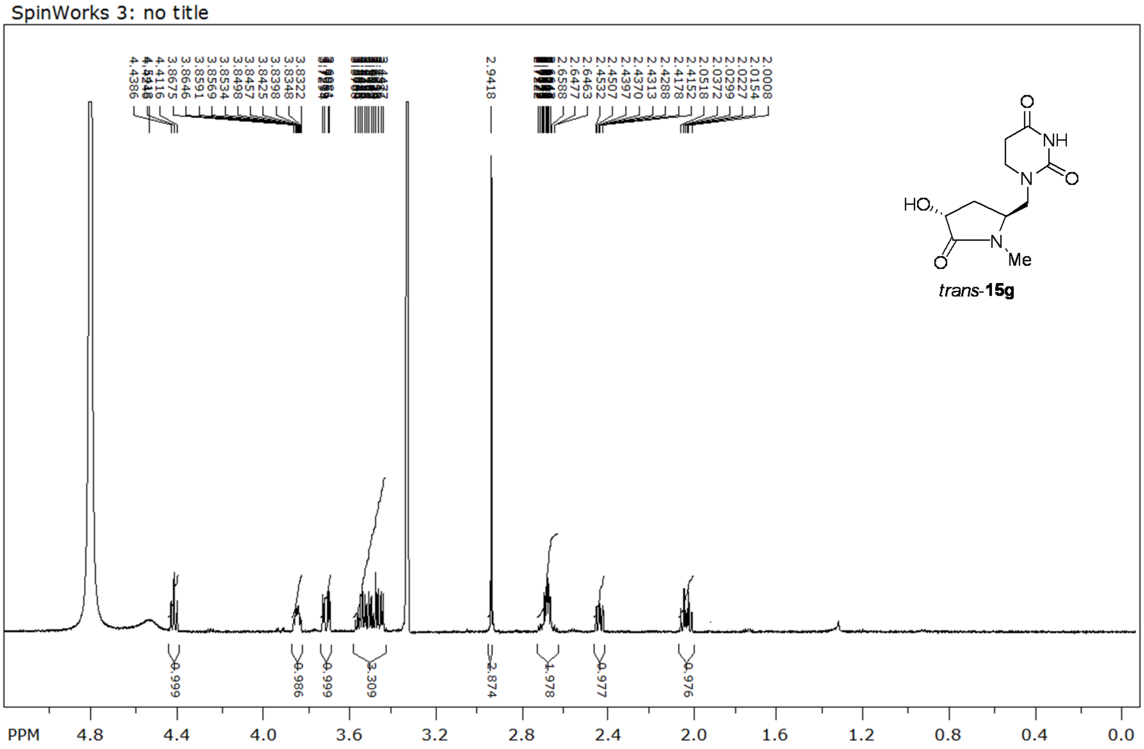

file: E:\gamma jrg\jrg-1762\10\fid expt: <zg30>
transmitter freq.: 600.263707 MHz
time domain size: 65536 points
width: 12335.53 Hz = 20.5502 ppm = 0.188225 Hz/pt
number of scans: 16

freq. of 0 ppm: 600.260000 MHz
processed size: 32768 complex points
LB: 0.000 GF: 0.0000
Hz/cm: 127.089 ppm/cm: 0.21172

SpinWorks 3: no title

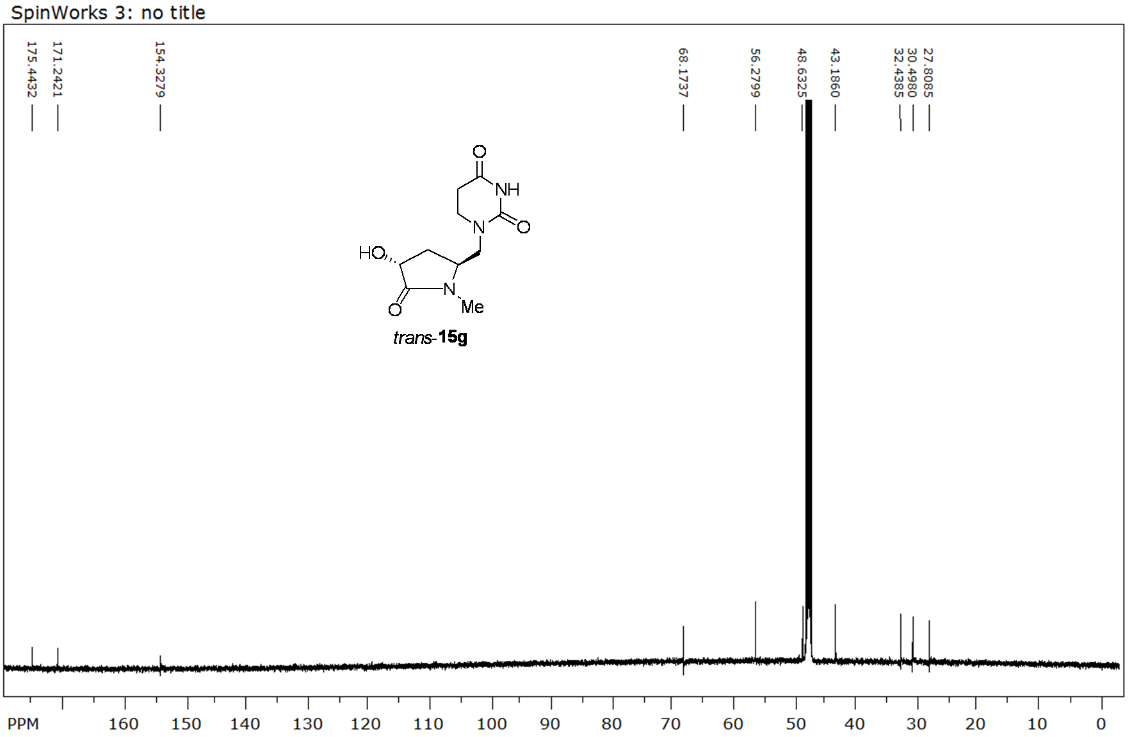

file: E:\gamma jrg\jrg-1525\11\fid expt: <zgpg30>
transmitter freq.: 150.950591 MHz
time domain size: 65536 points
width: 36057.69 Hz = 238.8708 ppm = 0.550197 Hz/pt
number of scans: 6000

freq. of 0 ppm: 150.935497 MHz
processed size: 32768 complex points
LB: 0.000 GF: 0.0000
Hz/cm: 1108.918 ppm/cm: 7.34623

SpinWorks 3: no title

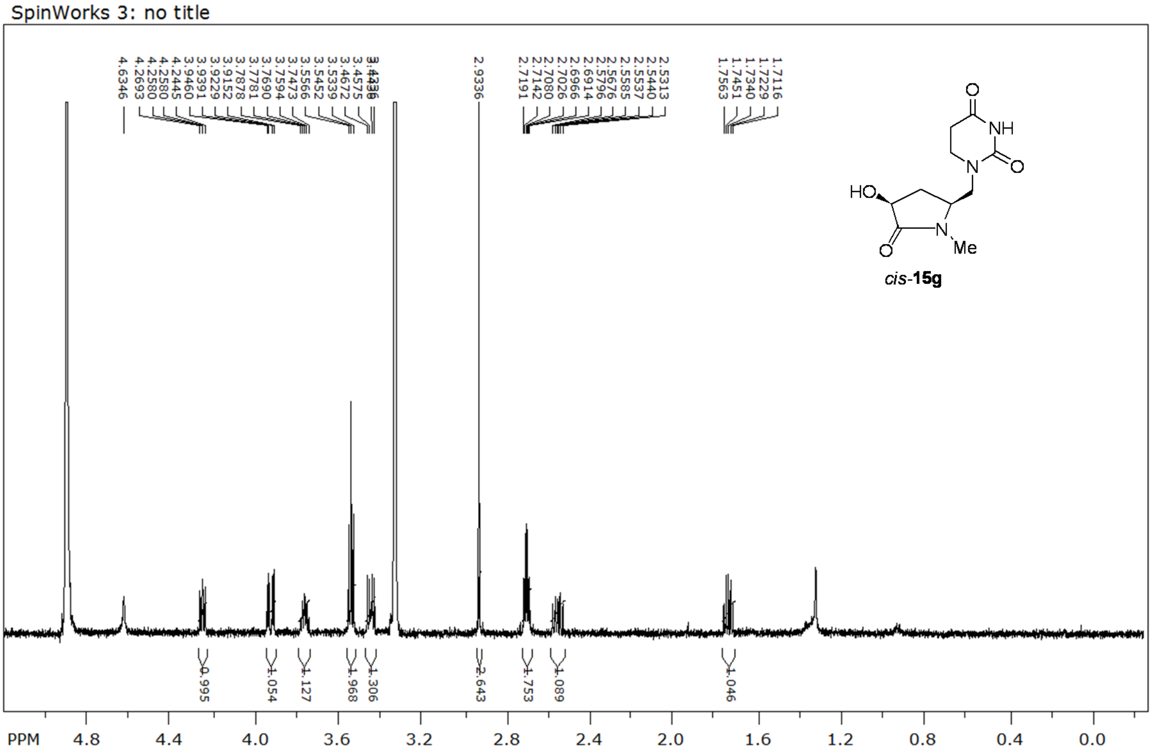

file: E:\gamma jrg\jrg-1965\10\fid expt: <zg30>
transmitter freq.: 600.263707 MHz
time domain size: 65536 points
width: 12335.53 Hz = 20.5502 ppm = 0.188225 Hz/pt
number of scans: 16

freq. of 0 ppm: 600.260000 MHz
processed size: 32768 complex points
LB: 0.000 GF: 0.0000
Hz/cm: 131.163 ppm/cm: 0.21851

SpinWorks 3: no title

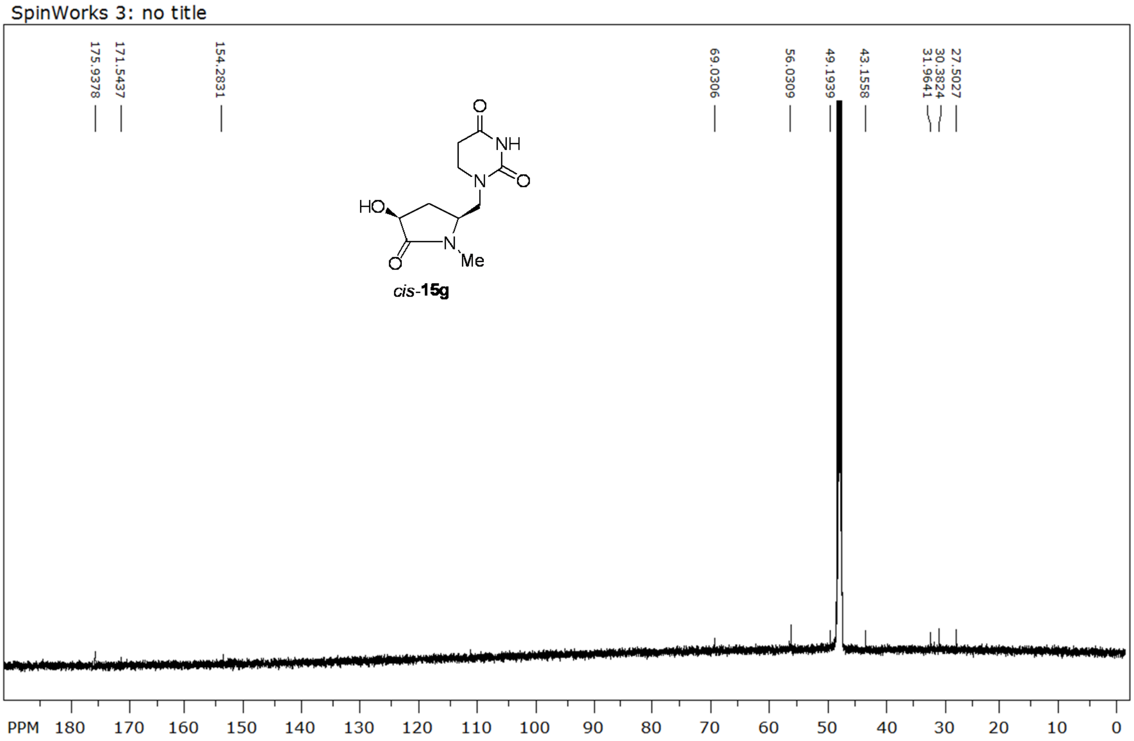

file: E:\gamma jrg\jrg-1965\11\fid expt: <zgpg30>
transmitter freq.: 150.950591 MHz
time domain size: 65536 points
width: 36057.69 Hz = 238.8708 ppm = 0.550197 Hz/pt
number of scans: 10240

freq. of 0 ppm: 150.935497 MHz
processed size: 32768 complex points
LB: 0.000 GF: 0.0000
Hz/cm: 1167.658 ppm/cm: 7.73537

SpinWorks 3: no title

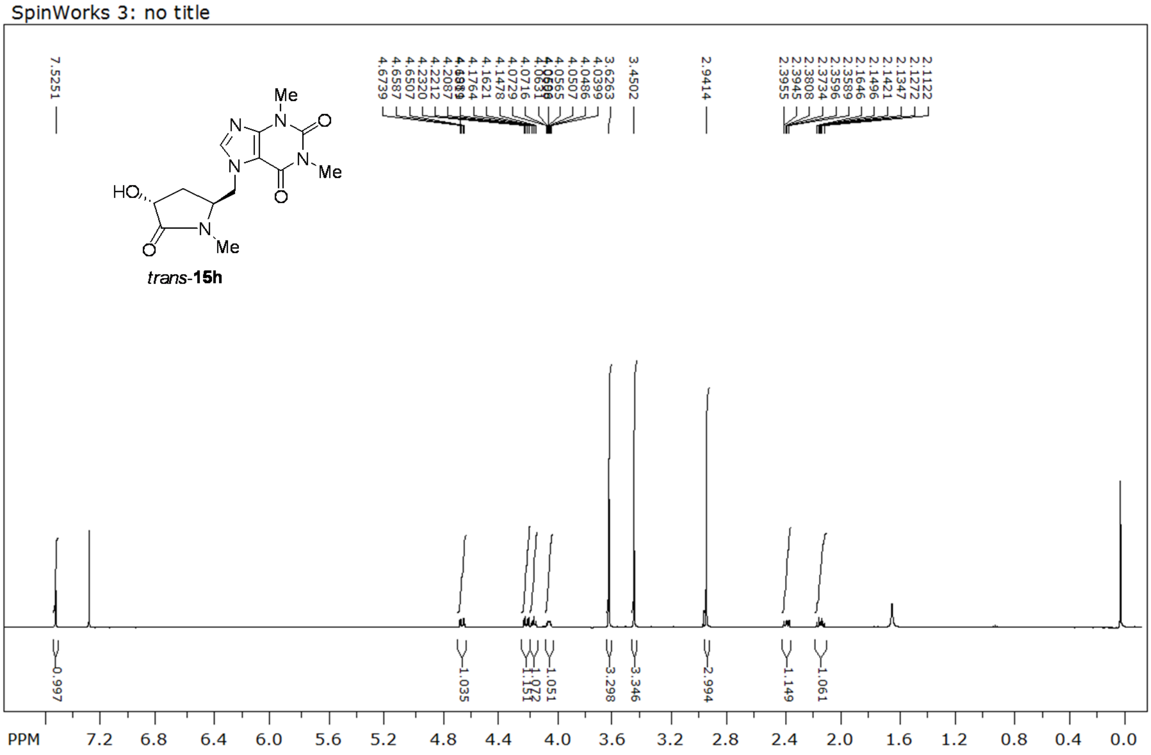

file: E:\gamma jrg\jrg-1501\10\fid expt: <zg30>
transmitter freq.: 600.263707 MHz
time domain size: 65536 points
width: 12335.53 Hz = 20.5502 ppm = 0.188225 Hz/pt
number of scans: 16

freq. of 0 ppm: 600.260000 MHz
processed size: 32768 complex points
LB: 0.000 GF: 0.0000
Hz/cm: 192.535 ppm/cm: 0.32075

SpinWorks 3: no title

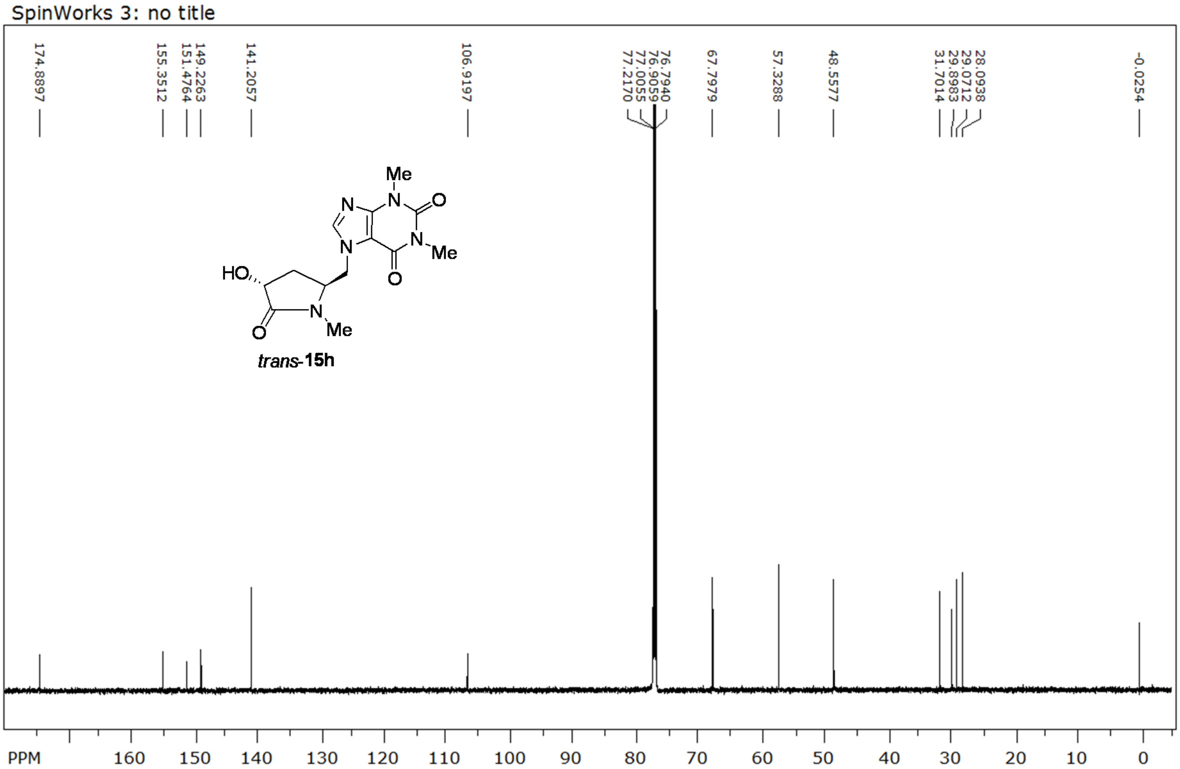

file: E:\gamma jrg\jrg-1501\11\fid expt: <zgpg30>
transmitter freq.: 150.950591 MHz
time domain size: 65536 points
width: 36057.69 Hz = 238.8708 ppm = 0.550197 Hz/pt
number of scans: 6000

freq. of 0 ppm: 150.935497 MHz
processed size: 32768 complex points
LB: 0.000 GF: 0.0000
Hz/cm: 1120.825 ppm/cm: 7.42511

SpinWorks 3: no title

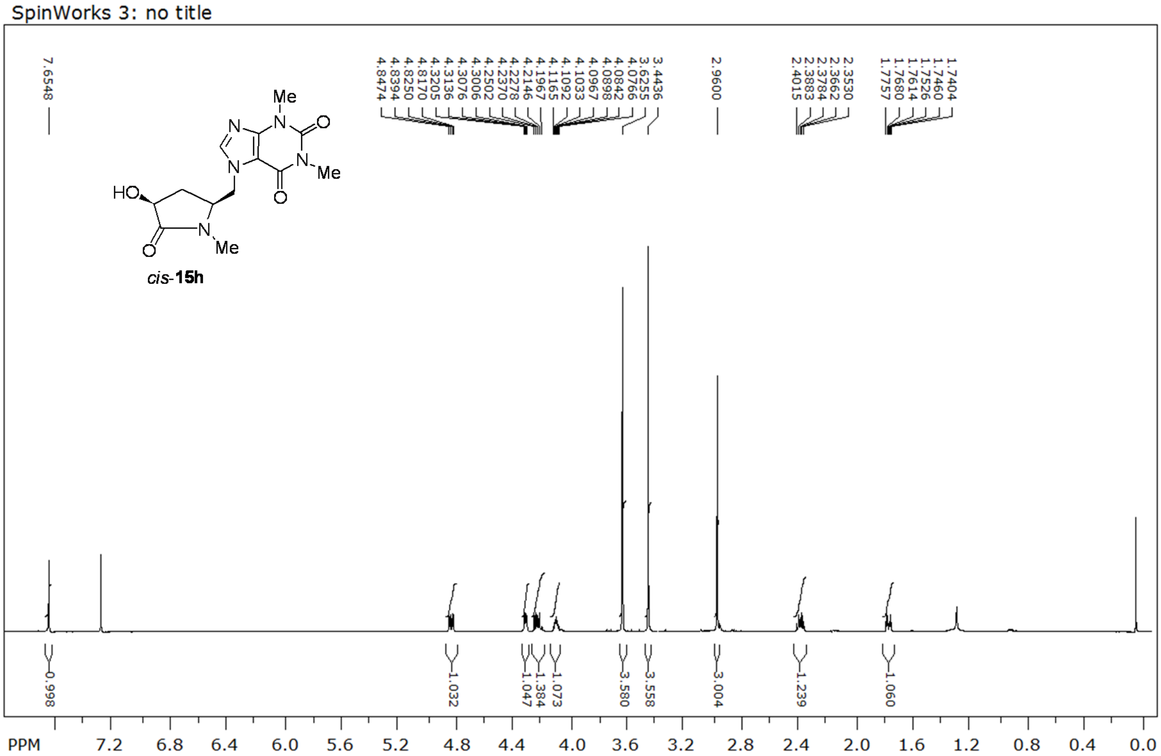

file: E:\gamma jrg\jrg-1506\10\fid expt: <zg30>
transmitter freq.: 600.263707 MHz
time domain size: 65536 points
width: 12335.53 Hz = 20.5502 ppm = 0.188225 Hz/pt
number of scans: 16

freq. of 0 ppm: 600.260000 MHz
processed size: 32768 complex points
LB: 0.000 GF: 0.0000
Hz/cm: 193.621 ppm/cm: 0.32256

SpinWorks 3: no title

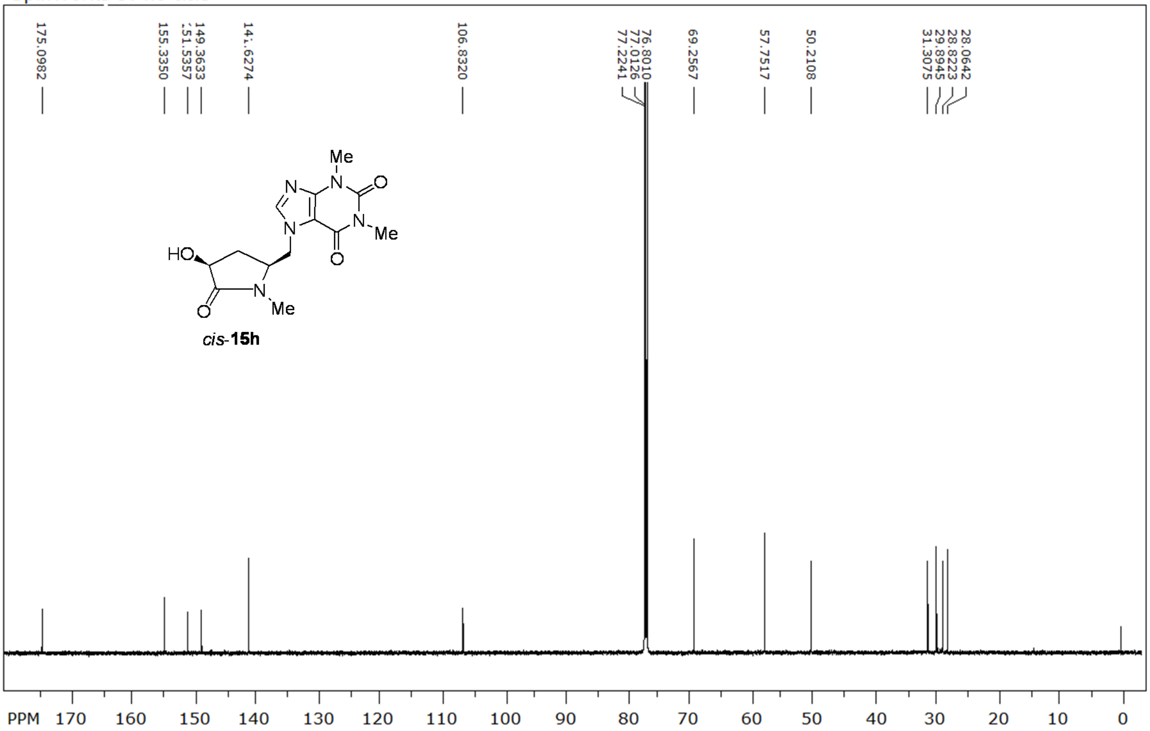

file: E:\gamma jrg\jrg-1506\11\fid expt: <zgpg30>
transmitter freq.: 150.950591 MHz
time domain size: 65536 points
width: 36057.69 Hz = 238.8708 ppm = 0.550197 Hz/pt
number of scans: 3072

freq. of 0 ppm: 150.935497 MHz
processed size: 32768 complex points
LB: 0.000 GF: 0.0000
Hz/cm: 1115.268 ppm/cm: 7.38830

SpinWorks 3:

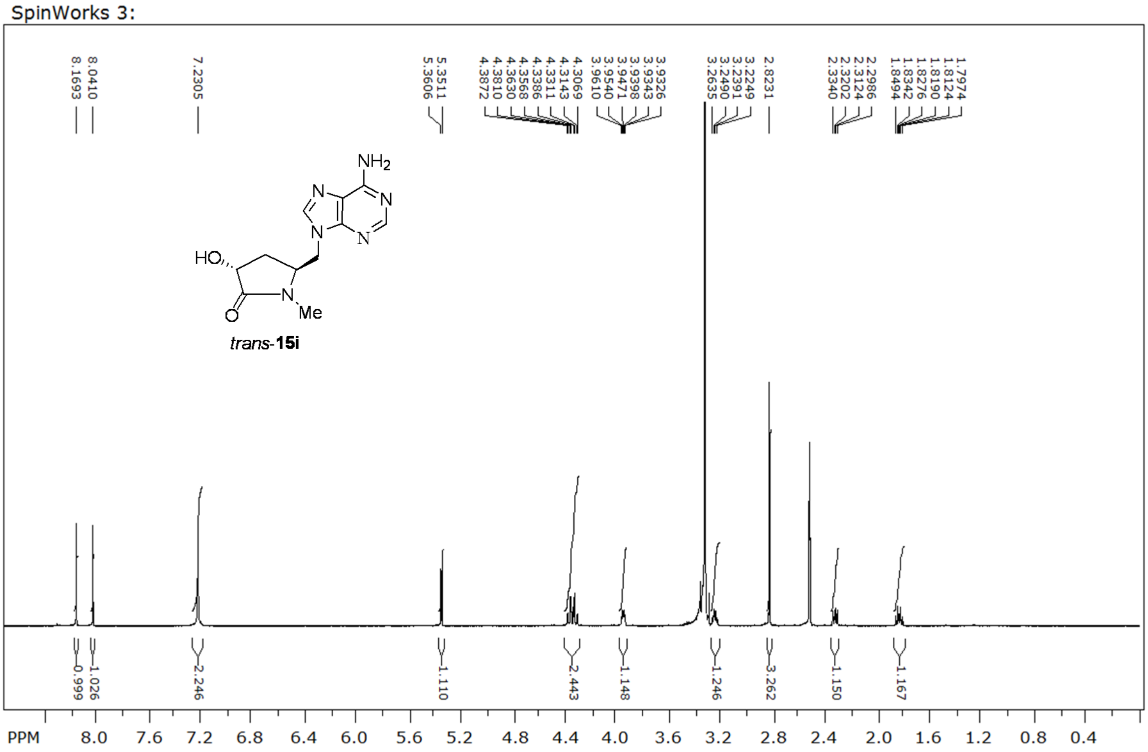

file: E:\gamma jrg\jrg-1262\1\fid expt: <zg30>
transmitter freq.: 600.261989 MHz
time domain size: 65536 points
width: 12335.53 Hz = 20.5502 ppm = 0.188225 Hz/pt
number of scans: 16

freq. of 0 ppm: 600.260000 MHz
processed size: 32768 complex points
LB: 0.000 GF: 0.0000
Hz/cm: 210.186 ppm/cm: 0.35016

SpinWorks 3: no title

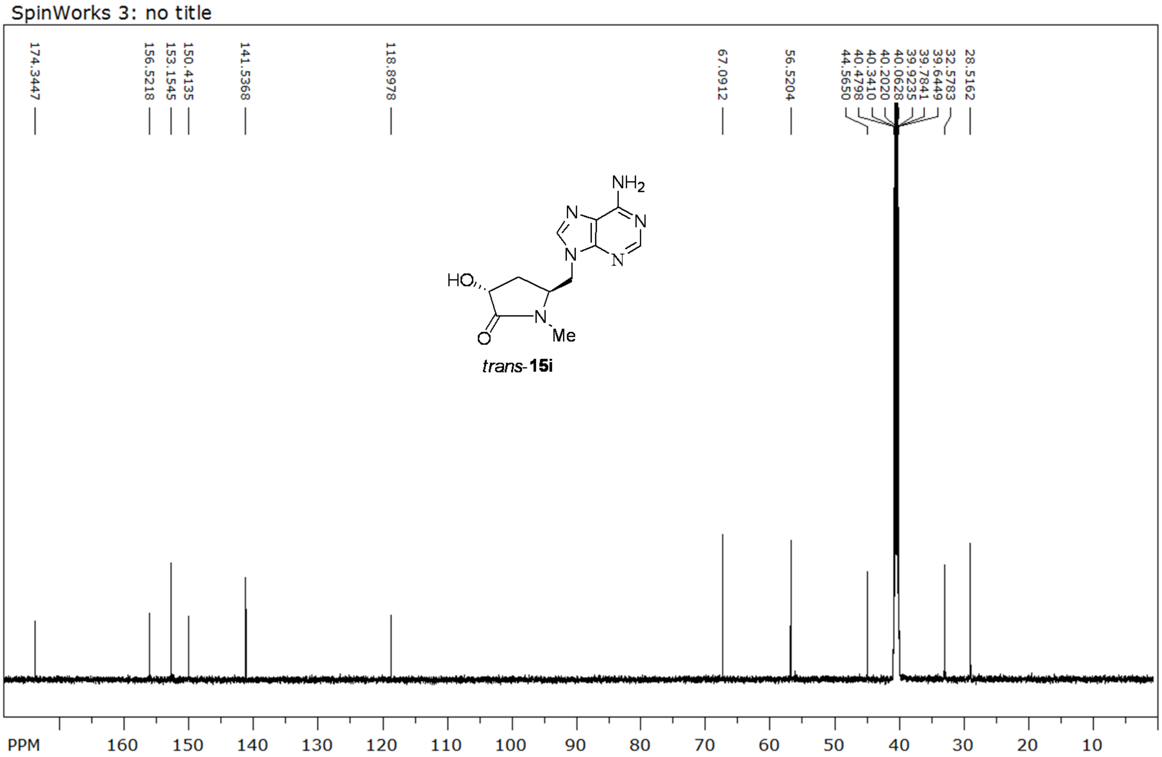

file: E:\gamma jrg\jrg-1272\10\fid expt: <zgpg30>
transmitter freq.: 150.950591 MHz
time domain size: 65536 points
width: 36057.69 Hz = 238.8708 ppm = 0.550197 Hz/pt
number of scans: 5000

freq. of 0 ppm: 150.935497 MHz
processed size: 32768 complex points
LB: 0.000 GF: 0.0000
Hz/cm: 1081.929 ppm/cm: 7.16744

SpinWorks 3:

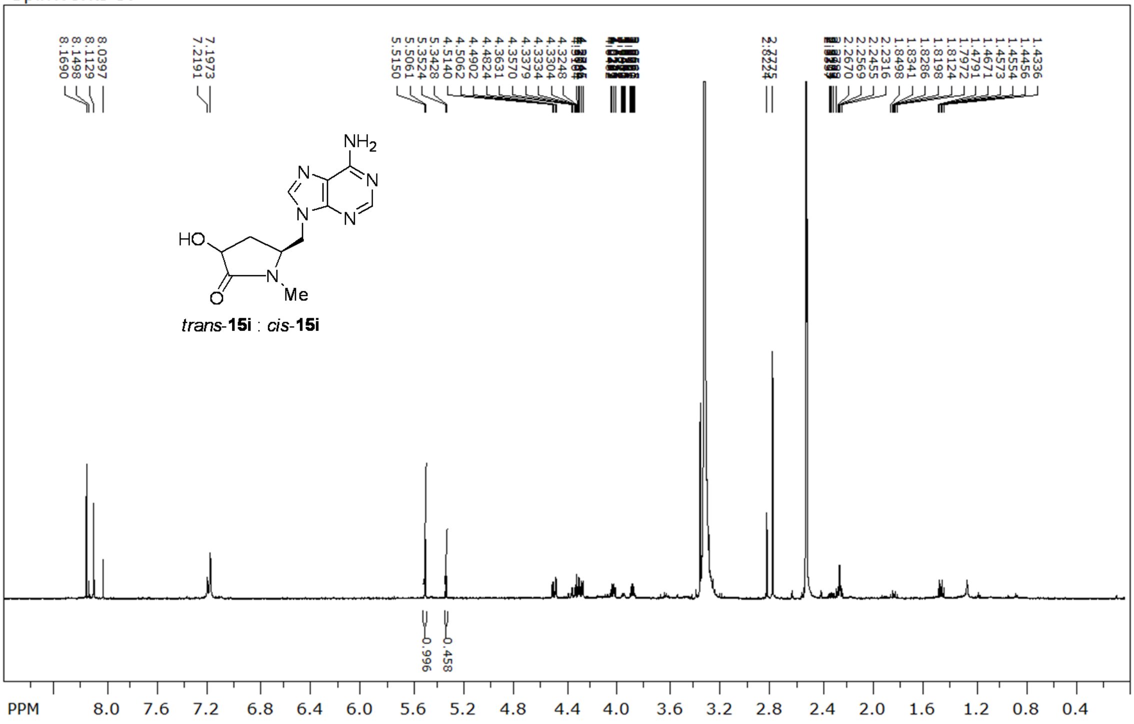

file: E:\gamma jrg\jrg-1311\1\fid expt: <zg30>
transmitter freq.: 600.263707 MHz
time domain size: 65536 points
width: 12335.53 Hz = 20.5502 ppm = 0.188225 Hz/pt
number of scans: 64

freq. of 0 ppm: 600.260000 MHz
processed size: 32768 complex points
LB: 0.000 GF: 0.0000
Hz/cm: 211.544 ppm/cm: 0.35242

jrg-1311 2 1 C:\NMR

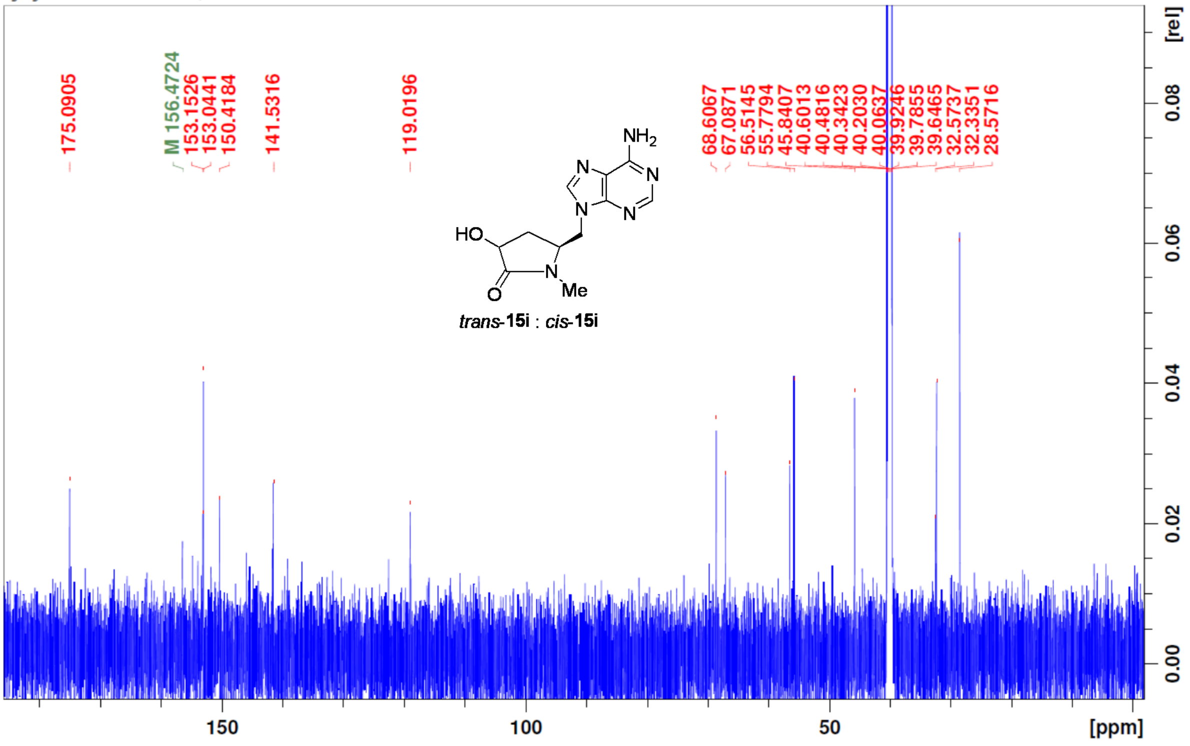

SpinWorks 3:

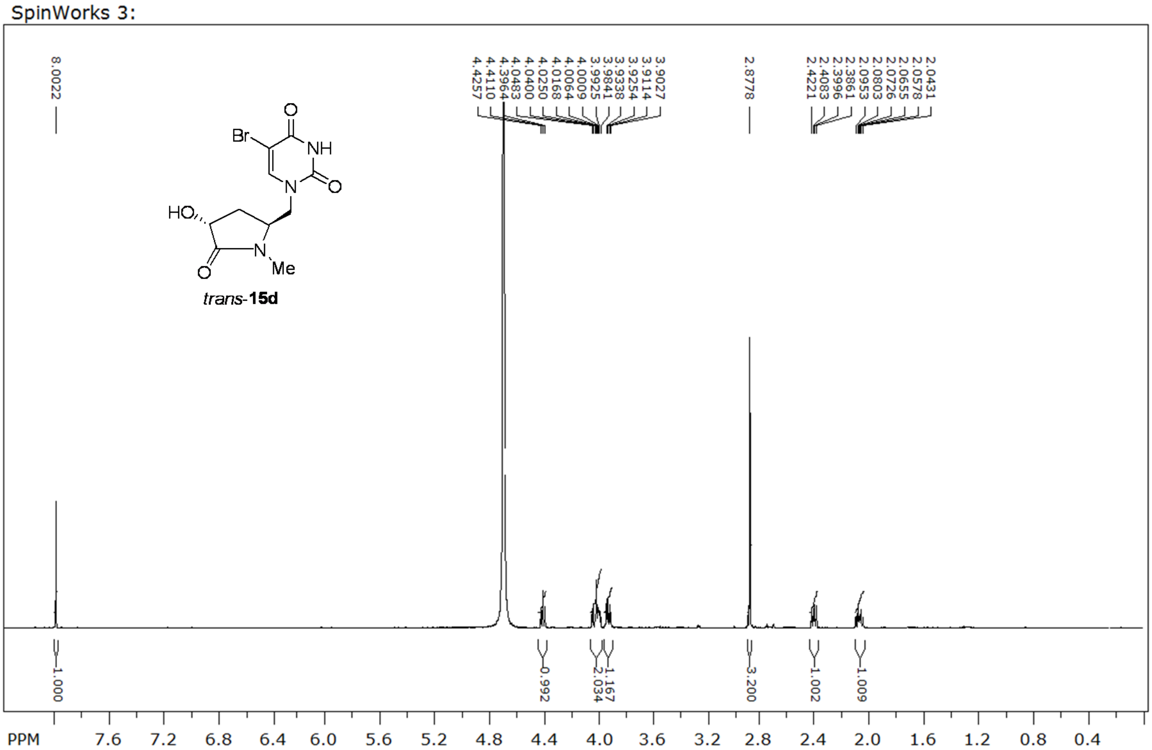

file: E:\gamma jrg\jrg-1740\1\fid expt: <zg30>
transmitter freq.: 600.263707 MHz
time domain size: 65536 points
width: 12335.53 Hz = 20.5502 ppm = 0.188225 Hz/pt
number of scans: 16

freq. of 0 ppm: 600.260000 MHz
processed size: 32768 complex points
LB: 0.000 GF: 0.0000
Hz/cm: 202.039 ppm/cm: 0.33658

SpinWorks 3: no title

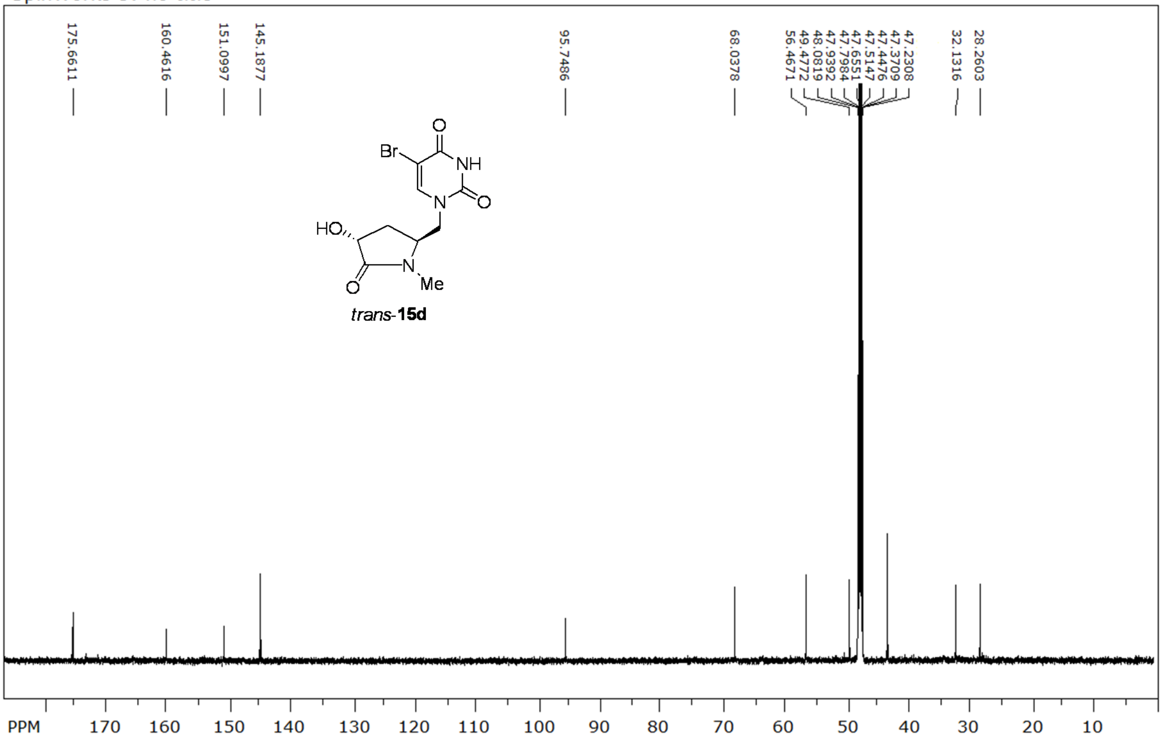

file: E:\gamma jrg\jrg-1370\11\fid expt: <zgpg30>
transmitter freq.: 150.950591 MHz
time domain size: 65536 points
width: 36057.69 Hz = 238.8708 ppm = 0.550197 Hz/pt
number of scans: 3072

freq. of 0 ppm: 150.935497 MHz
processed size: 32768 complex points
LB: 0.000 GF: 0.0000
Hz/cm: 1127.969 ppm/cm: 7.47244

SpinWorks 3:

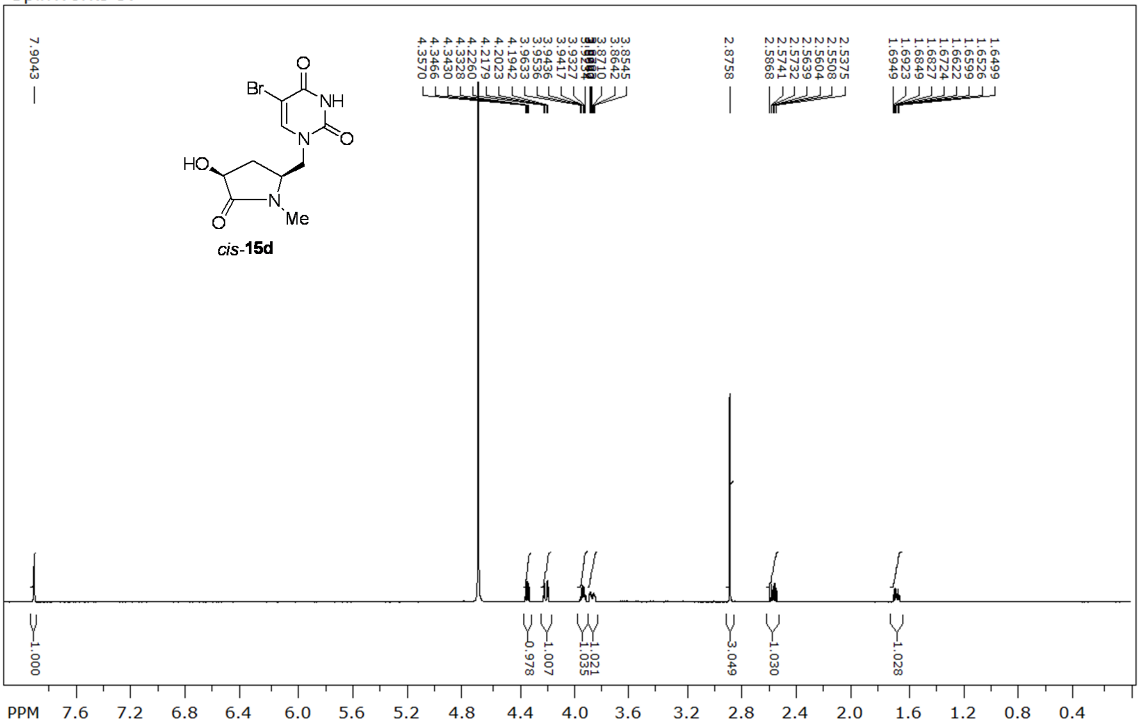

file: E:\gamma jrg\jrg-1748\1\fid expt: <zg30>
transmitter freq.: 600.263707 MHz
time domain size: 65536 points
width: 12335.53 Hz = 20.5502 ppm = 0.188225 Hz/pt
number of scans: 16

freq. of 0 ppm: 600.260000 MHz
processed size: 32768 complex points
LB: 0.000 GF: 0.0000
Hz/cm: 195.793 ppm/cm: 0.32618

SpinWorks 3: no title

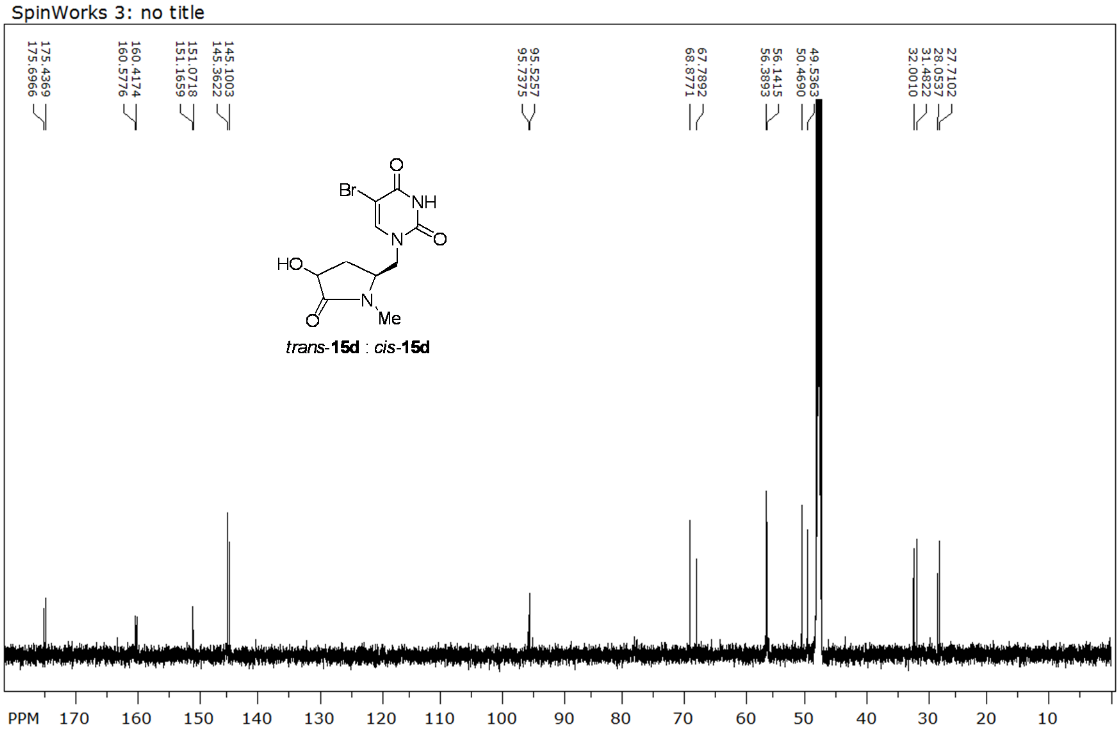

file: E:\gamma jrg\jrg-1369\11\fid expt: <zgpg30>
transmitter freq.: 150.950591 MHz
time domain size: 65536 points
width: 36057.69 Hz = 238.8708 ppm = 0.550197 Hz/pt
number of scans: 3072

freq. of 0 ppm: 150.935497 MHz
processed size: 32768 complex points
LB: 0.000 GF: 0.0000
Hz/cm: 1104.155 ppm/cm: 7.31468

SpinWorks 3: no title

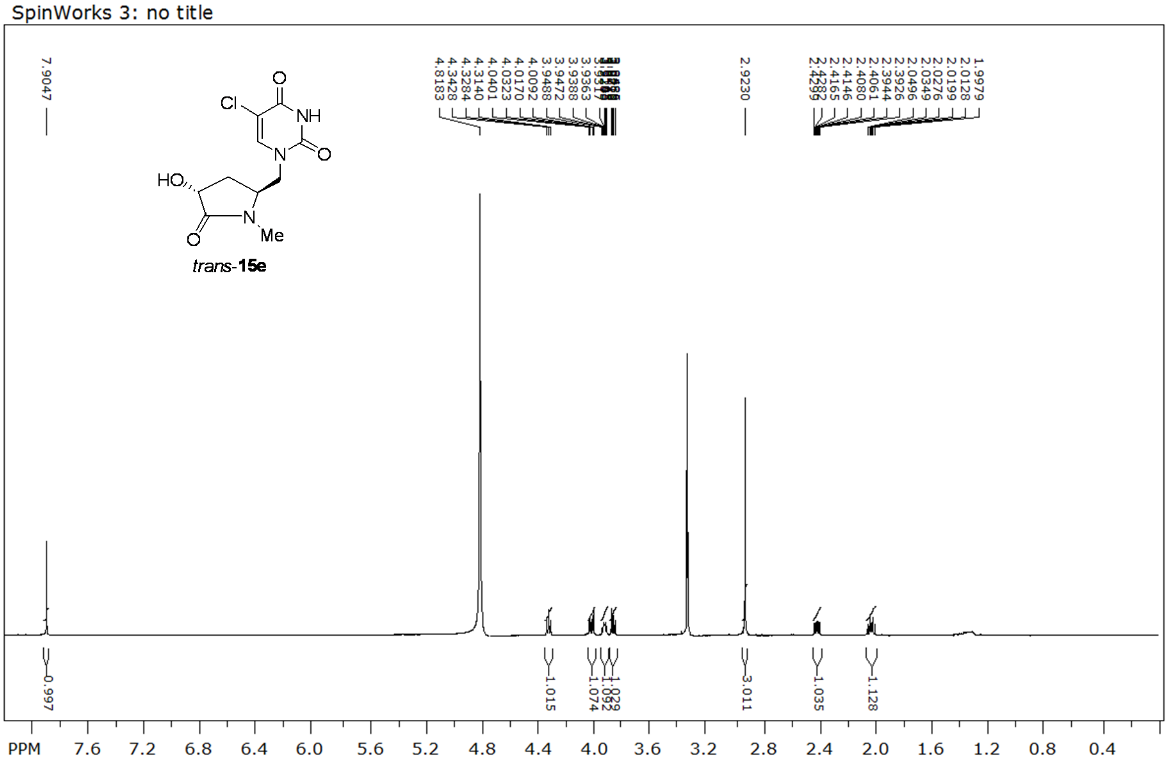

file: E:\gamma jrg\jrg-1647\10\fid expt: <zg30>
transmitter freq.: 600.263707 MHz
time domain size: 65536 points
width: 12335.53 Hz = 20.5502 ppm = 0.188225 Hz/pt
number of scans: 16

freq. of 0 ppm: 600.260000 MHz
processed size: 32768 complex points
LB: 0.000 GF: 0.0000
Hz/cm: 197.694 ppm/cm: 0.32935

SpinWorks 3: no title

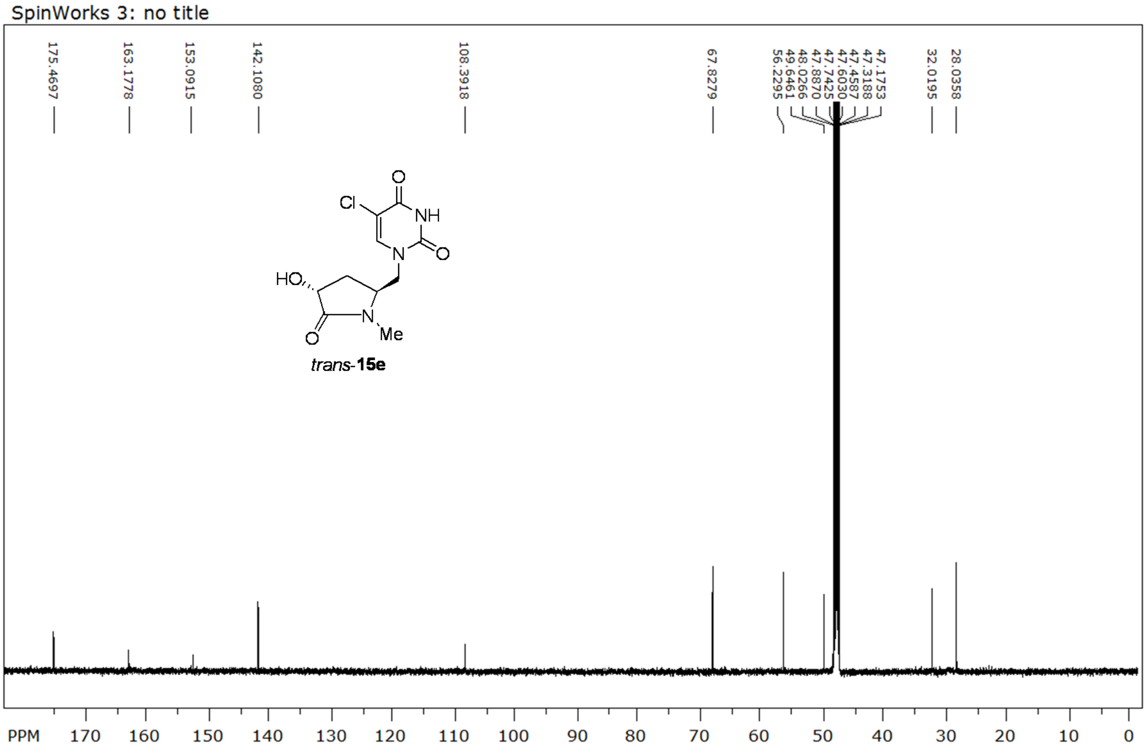

file: E:\gamma jrg\jrg-1647\11\fid expt: <zgpg30>
transmitter freq.: 150.950591 MHz
time domain size: 65536 points
width: 36057.69 Hz = 238.8708 ppm = 0.550197 Hz/pt
number of scans: 4000

freq. of 0 ppm: 150.935497 MHz
processed size: 32768 complex points
LB: 0.000 GF: 0.0000
Hz/cm: 1117.650 ppm/cm: 7.40408

SpinWorks 3: no title

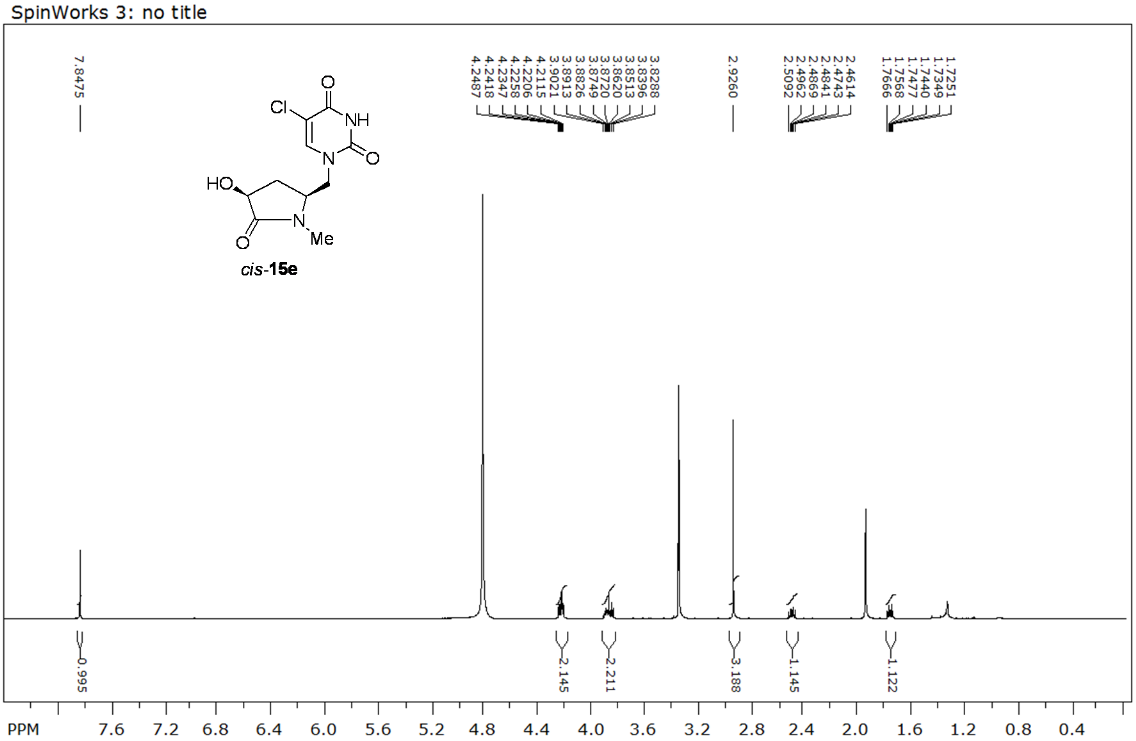

file: E:\gamma jrg\jrg-1648\10\fid expt: <zg30>
transmitter freq.: 600.263707 MHz
time domain size: 65536 points
width: 12335.53 Hz = 20.5502 ppm = 0.188225 Hz/pt
number of scans: 16

freq. of 0 ppm: 600.260000 MHz
processed size: 32768 complex points
LB: 0.000 GF: 0.0000
Hz/cm: 203.125 ppm/cm: 0.33839

SpinWorks 3: no title

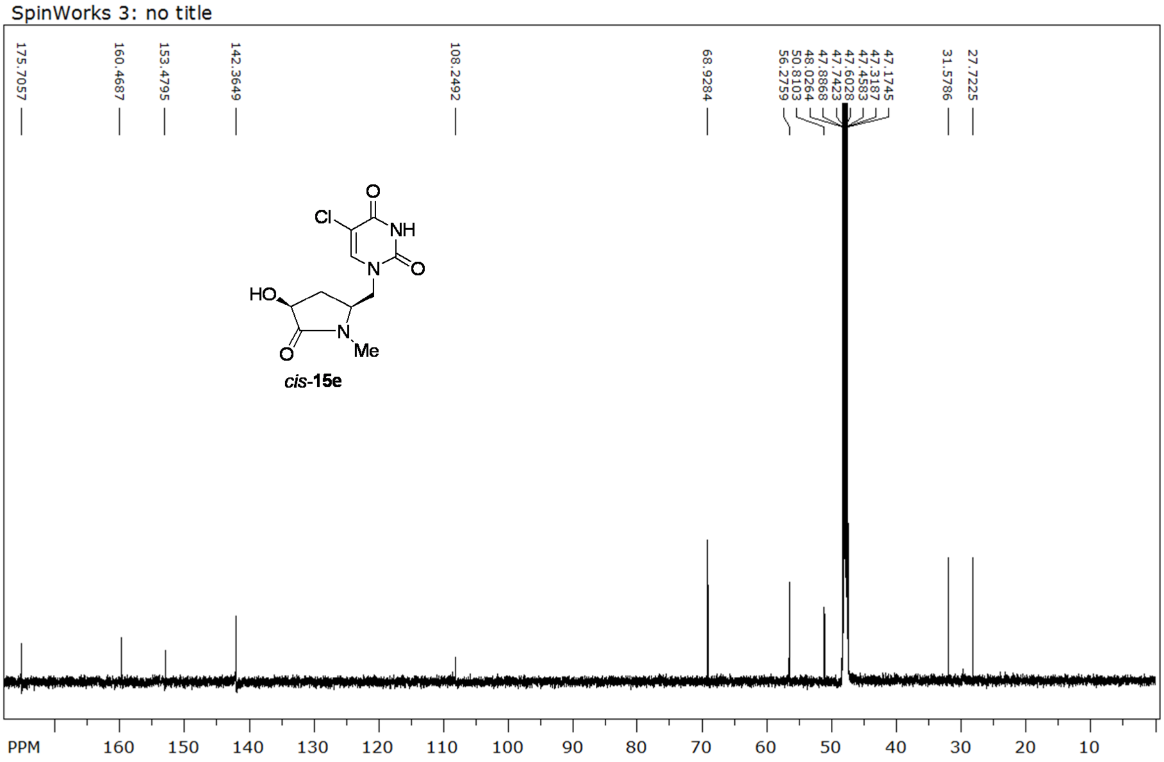

file: E:\gamma jrg\jrg-1648\11\fid expt: <zgpg30>
transmitter freq.: 150.950591 MHz
time domain size: 65536 points
width: 36057.69 Hz = 238.8708 ppm = 0.550197 Hz/pt
number of scans: 4000

freq. of 0 ppm: 150.935497 MHz
processed size: 32768 complex points
LB: 0.000 GF: 0.0000
Hz/cm: 1081.135 ppm/cm: 7.16218
